# Lineage-specific evolution, structural diversity, and activity of R2 retrotransposons in animals

**DOI:** 10.1186/s13059-026-04073-3

**Published:** 2026-04-14

**Authors:** Nozhat T. Hassan, Briana Van Treeck, Anthony Rodríguez-Vargas, Anna E. Sheppard, Kathleen Collins, David L. Adelson

**Affiliations:** 1 School of Biological Sciences, Adelaide University, Adelaide, Australia; 2 Department of Molecular and Cell Biology, University of California Berkeley, Berkeley, CA USA; 3 South Australian Museum, Adelaide, Australia

## Abstract

**Background:**

Retrotransposons play outsized roles in the evolution of gene regulation, genome function, and disease pathogenesis, and more recently they have sparked interest as instruments for new gene therapy approaches. R2 retrotransposons insert site-specifically into the multicopy genes encoding 28S ribosomal RNA at a target sequence conserved broadly across eukaryotic evolution. R2 retrotransposons have been detected in many animals, but previous surveys have been limited in scope and methodology.

**Results:**

Here, we substantially expand the known distribution of R2 retrotransposons from previously unrepresented or underrepresented taxonomic groups, ranging from ctenophores to amphibians and reptiles. We discover diverse R2 domain architectures and motifs and identify many new avian R2 candidates for genome engineering development. Overall, phylogenetic analyses reveal two highly successful R2 lineages. We describe lineage-distinctive features of the N-terminal DNA recognition domains and reverse-transcriptase domain signatures. Within a lineage, R2 protein sequences do not necessarily preserve the unifying configuration of N-terminal domains assumed in the current clade classification scheme. We show that recombinant R2 proteins with distinctive domain architectures and distribution across major animal classes support target-primed reverse transcription with conserved site specificity.

**Conclusions:**

Our analysis of the surprisingly varied domain architectures that support target-site specificity informs new R2 classification criteria and provides a greatly expanded foundation for additional structure/function insights about DNA binding selectivity. This expanded perspective on R2 evolution informs approaches for engineering therapeutic gene insertion technologies and offers an opportunity to investigate the conservation and diversification of retrotransposons.

**Supplementary Information:**

The online version contains supplementary material available at 10.1186/s13059-026-04073-3.

## Background

Non-long terminal repeat (non-LTR) retrotransposons are generally ubiquitous across eukaryotes, contributing to both genome evolution and disease through their mobility and other activities [1, 2]. Non-LTR retrotransposons are present across animals in different combinations of retrotransposon identities and abundances, suggestive of dramatically differential mobility and molecular evolution resulting from arms races with transposon repression mechanisms within the host genome [3]. Recent efforts have adapted non-LTR retrotransposon machinery into new tools for gene insertion, promising potential new approaches for disease therapy. Non-LTR retrotransposons with minimal target-site sequence requirements can be used to randomly place gene insertions or can be fused with targeting determinants [2, 4]. On the other hand, retrotransposons that have evolved very restrictive target-site specificity are useful for gene addition to specific loci, preferably to safe harbour regions of the human genome [5]. Precise RNA-mediated insertion of transgenes (PRINT) is one such technology that relies on the site-specificity of one of the evolutionarily most widespread non-LTR retrotransposons, R2 [6].

The R2 family of non-LTR retrotransposons inserts site-specifically into the 28S ribosomal RNA (rRNA) region of genes encoding the large rRNA precursor (rDNA loci), present in genomes as tandemly repeated rDNA arrays [7]. R2s lack an internal promoter and at least in some species, are dependent on the rDNA RNA polymerase I promoter for proliferation [7, 8]. The single R2-encoded protein (R2p) recognises and nicks the 28S target site and uses the nicked DNA as a primer for cDNA synthesis directly into the genome, a process termed target-primed reverse transcription (TPRT) [7]. This requires coordinated R2p reverse transcriptase (RT) and endonuclease (EN) activities [9]. The most N-terminal portion of R2p contains the majority of the DNA-binding surface for the target site [10]. Different domain architectures of the N-terminal region, with a variable 1–3 zinc-fingers (ZnFs) preceding an adjacent Myb domain, have been the basis of classifying R2 elements into 4 clades further divided into 11 subclades [6, 11]. ZnFs are numbered with the conserved ZnF1 sitting N-terminal to the Myb domain and subsequent ZnFs progressing towards the N-terminus, with ZnF2 differing from ZnF1 and ZnF3 in a cysteine (C) and histidine (H) pattern of CCHC instead of CCHH. Current R2 classification thus has clade A (ZnF3 + ZnF2 + ZnF1), clades B and C (ZnF2 + ZnF1 and ZnF3 + ZnF1, respectively), and clade D (ZnF1 only), with A-clade and D-clade R2p appearing most common [12, 13].

R2s were discovered within the genome of *Drosophila melanogaster* (fruit fly) and were initially thought to be intronic sequences [14]. R2s have since been found in a variety of organisms through the generation of genomic sequence data. They are notably found in Ctenophora and extend to Reptilia and Aves but not mammals [12]. This indicates that R2s have persisted since near the beginning of multicellular eukaryotic life approximately 800 million years ago [7, 11, 15]. However, too few sequences have been identified to date to understand the phylogenetic scope of full-length R2 persistence and whether particular R2 N-terminal domain configurations occur only in specific phylogenetic groups. Prior to this work, the known distribution of R2s from RepBase [16–18] consisted of 185 sequences from 144 species, with the majority from arthropods; just under 60 were full-length R2s with an entire, unambiguous R2p open reading frame (ORF) with flanking 5’ and 3’ untranslated regions (UTRs) embedded in 28S rDNA.

Here, we created an R2 discovery workflow that used long-read sequencing data and genome assemblies to curate over 330 new R2 sequences from major classes of Metazoan organisms. We identified the first R2s in Amphibia and Chondrichthyes and significantly expanded known R2s in reptiles, particularly in snakes. We discovered a surprising diversity of R2 N-terminal domain structures that does not fall within the current classification scheme [7, 12]. This diversification is overlaid on the propagation of two anciently diverged and highly successful lineages, each with co-evolving domains, and one lineage with a co-folded zinc finger pair of nucleic acid interaction domains that appears unique to R2. Our biochemical and cellular assays of a broad sampling of recombinant R2p expressed in human cells confirm the conservation of target site specificity despite differences in N-terminal domains and large differences in relative endonuclease and TPRT activities. This thorough expansion of R2 phylogeny annotation and functional testing enables deeper insights into retrotransposon evolution as well as R2p structure/function and engineering.

## Results

### Significant expansion of R2 retrotransposons across Metazoa

We aimed to sample R2 retrotransposon phylogeny more broadly than previously undertaken to gain a deeper insight into how R2s have diversified during Metazoan evolution. Many sequences of R2s reported to date are incomplete, with only a partial ORF or truncated UTRs [16–18]. Furthermore, because genome sequencing in the past largely relied on short-read sequencing, R2 consensus sequences derived from alignments of multiple reads may not accurately represent an actual full-length R2 ORF. With the increasing availability of long-read data and improved genome assemblies, we sought to detect potentially functional R2s more comprehensively across major animal groups.

Initially, our strategy to find R2s in genomes of interest mirrored past studies: we used BLASTN + to search the genome of interest with R2 query sequences from each previously defined clade (A, B, C, and D). The top hits were extended in both the 5’ and 3’ directions to find flanking 28S rDNA sequences [12]. Depending on whether both sides of the target site were located, sequences were annotated as complete or incomplete/truncated. However, this strategy often failed to return full-length R2s. Genome assemblies are not necessarily reliable regarding repetitive structures, especially of R2s, which are repeats nested within the larger repeats of an rDNA array [12, 19]. We hypothesised that some missing motifs could be due to misassembly rather than the absence of a full-length R2 in the organism. To address this limitation, we parsed long-read genome sequencing data with R2 query sequences when genome assemblies returned truncated R2 ORFs (Table S1).

**Fig. 1:**
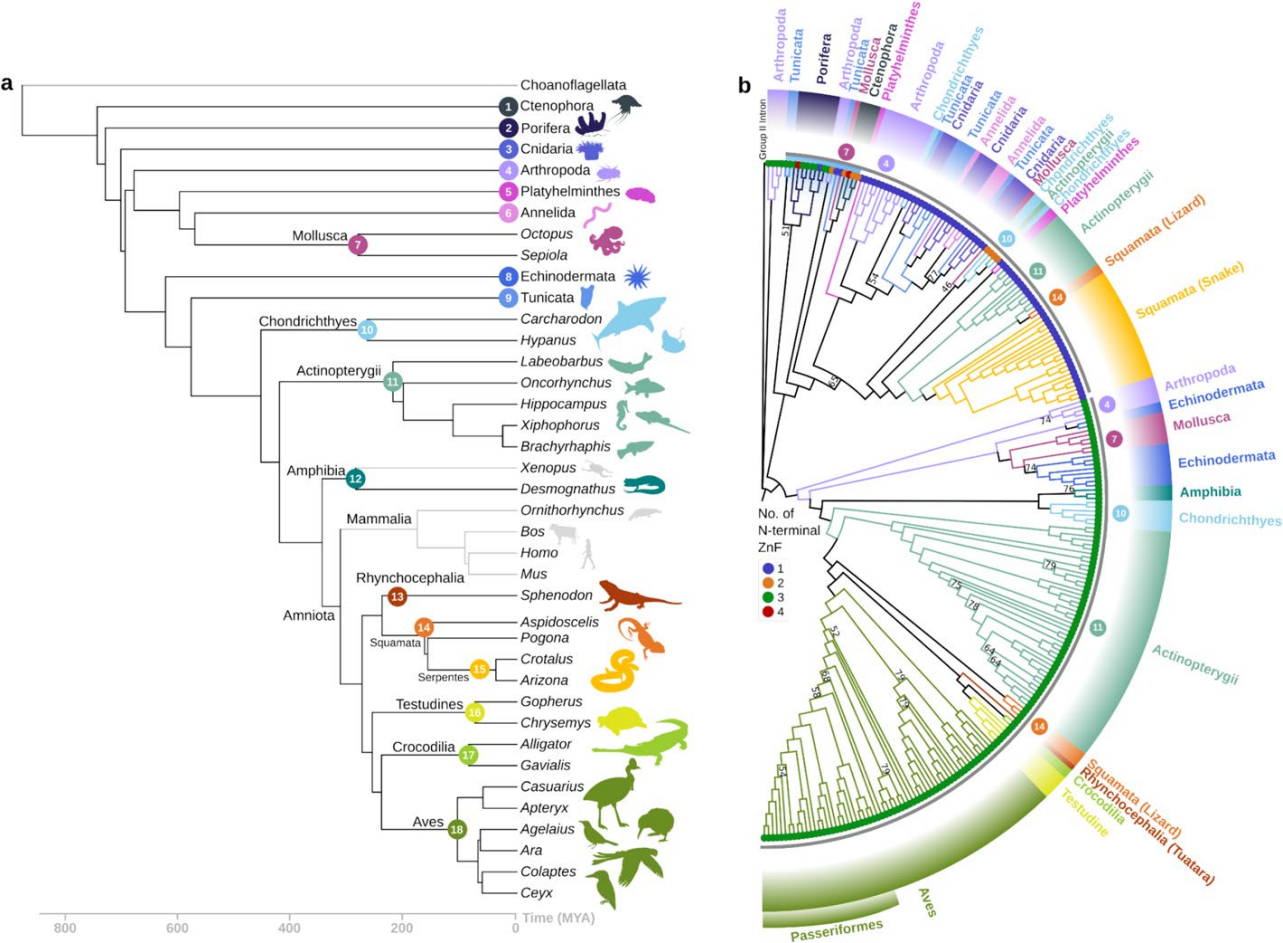
R2 retrotransposons are widely distributed across Metazoa but do not conform to the host species phylogeny. **a** Simplified divergence of major classes of animals relevant to this work, with Ctenophora as the sister group to all other animals, made with TimeTree. Time is shown as millions of years ago (MYA) on the bottom axis. Groups with no known R2s are shown in grey. The coloured numbers at some nodes of the host tree are to number major phyla for relation to (**b**). **b** Phylogenetic tree of R2 ORF amino acid sequences. Tree topology is illustrated with branch lengths that are arbitrary. Each tip is appended with a coloured circle symbol (blue—1 ZnF, orange—2 ZnF, green—3 ZnF, and red—4 ZnF), designating the number of ZnFs in the N-terminus of the R2p sequence. Working outwards: a small group of early-branching R2s with diverse ZnF arrangements at the top of the panel are highlighted with a light blue arc, and on the same arc trajectory, the grey arcs show the two major R2 lineages. Outer arcs denote phylogenetic groups that the R2s were curated from, with the colour code from (**a**). Coloured numbers at branches indicate instances where there is a major discordance from the host tree in (**a**). The order Passeriformes in Aves is annotated. Group II intron from *T. vestitus* was used as the outgroup. IQTree was used for tree building with 1000 replicates, and branch support values under 80 are shown at the nodes

Overall, after querying 360 genomes, we retrieved sequences from 300 total species in Actinopterygii, Amphibia, Arthropoda, Chondrichthyes, Cnidaria, Ctenophora, Echinodermata, Mollusca, Platyhelminthes, Porifera, Reptilia (Aves, Rhynchocephalia, Squamata, Testudines), and Tunicates (Table S2). Some species had more than one type of R2 (see below), bringing the total number of sequences to 330. Our focus was on phylogeny beyond the taxonomic groups most frequently sampled in past R2 studies [12, 20], thereby revealing a more widespread distribution of R2s than previously recognised (Fig. 1a). This includes the discovery of an expansion of R2s in Chondrichthyes (cartilaginous fish), Actinopterygii (ray-finned fish), and Reptilia, and the first reported amphibian R2s from salamanders (Fig. 1a, b). Full-length R2s in Reptilia persist across the wide phylogenetic diversity of Sphenodon (Tuatara), Squamata (snakes and lizards), Crocodilia, and Testudines (turtles) (Fig. 1a). Previously, the few identified snake and lizard R2s were truncated [12]; nonetheless, we recovered full-length R2 from many species in these groups.

**Fig. 2:**
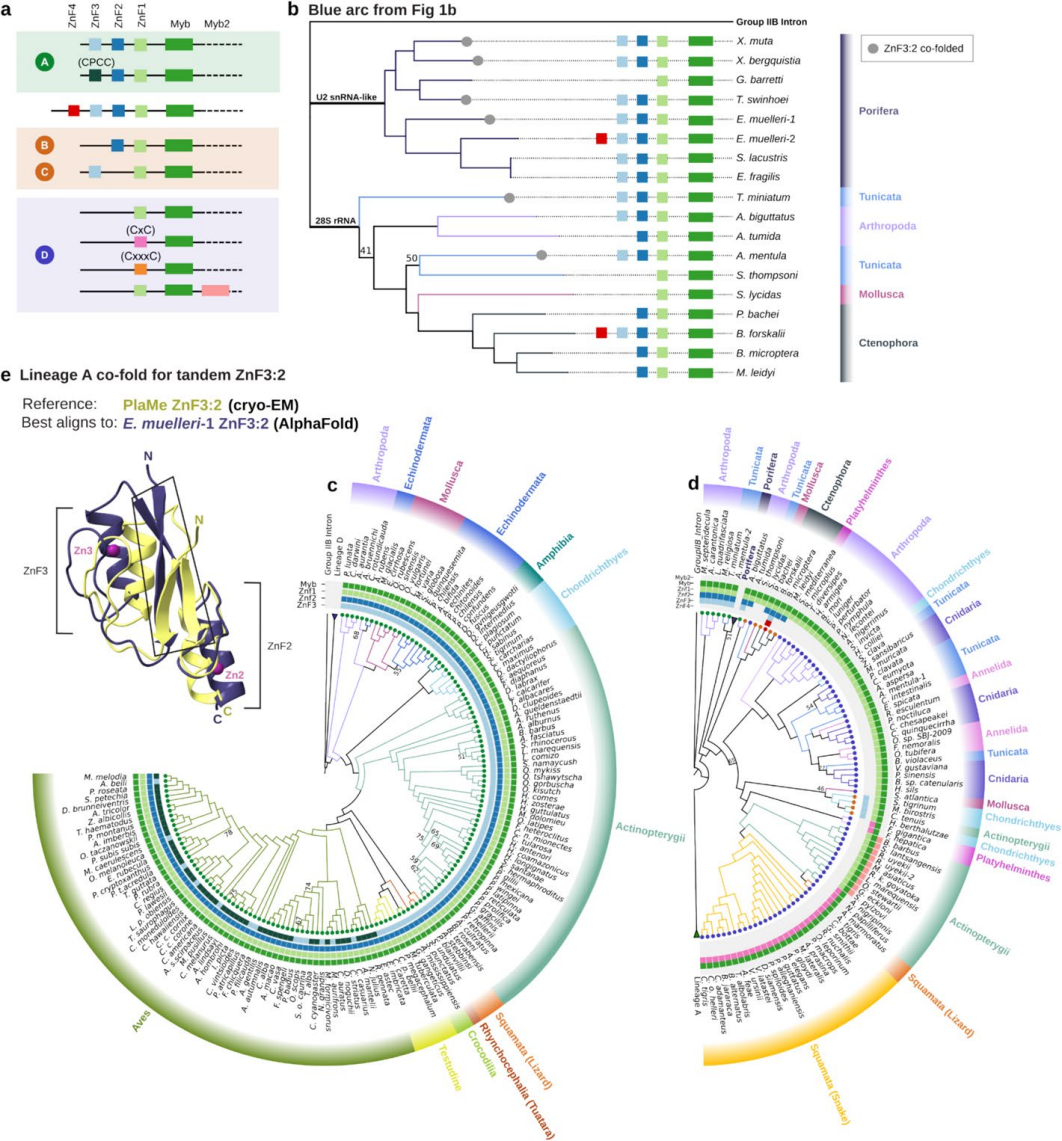
Structural changes in the N-terminus of R2p, and lineage D and A success. **a** Diversification of ZnF and Myb domain numbers and motif signatures. A—D represents the classification of R2s as clades based on previous studies. ZnF4, ZnF3, ZnF2, and ZnF1 are shown in red, light blue, blue, and light green boxes, respectively. Myb and Myb2 are shown in green and peach boxes. Alternative ZnF3 and ZnF1 motif sequences are annotated as dark green (CxxC to CPCC), pink (CxxC to CxC) and orange (CxxC to CxxxC). **b** Phylogenetic tree of R2 proteins and non-rDNA R2-homologous proteins re-built from the light blue arc in Fig. 1b shows the presence or absence of a co-folded ZnF3:2 based on AlphaFold3 predictions. **c**, **d** Phylogenetic trees with each tip appended with a coloured circle symbol designating the number of zinc fingers in the N-terminus of the R2 amino acid sequence (same as Fig. 1b). Coloured boxes for the arc representing each domain match the key in (**a**). In (**c**), the tree shows lineage A R2p with exclusively 3 ZnF. In (**d**), the tree shows lineage D R2p with predominantly D and some A, B and C domain architectures. Porifera R2-homologous proteins are collapsed, with their domain architectures shown in (**b**). Arthropod R2s from *M. septendecula*, *A. carantonica*, *L. quadrifasciata*, and *M. religiosa* sit outside the major lineage D branch. **e** AlphaFold-predicted structure of ZnF3:2 from *E. muelleri*−1 (purple) superimposed with cryo-EM structure of PlaMe (yellow) ZnF3:2 [22]. Zinc atoms are shown in magenta. ZnFs in *E. muelleri-1* are all CCHH type, in contrast to PlaMe CCHH and CCHC. Boxed in the aligned structures is the region where most non-covalent, intramolecular interactions concentrate between ZnFs. IQTree was used for tree building (**b**-**d**) with 1000 replicates and branch support values under 80 are shown at the nodes

While failure to detect R2 is not an absolute indicator of their absence, we suggest that the lack of identifiable R2 in several phylogenetic groups indicates stochastic evolutionary loss (Fig. 1a, tree lines and organism schematics in light grey). We also did not detect R2s in unicellular organisms, including Choanoflagellates (Table S3). As in previous studies, we defined sequences as R2 based on their homology to other known R2 retrotransposons and their insertion into the conserved 28S rDNA target site. We discovered that R2-homologous retrotransposon sequences in Porifera (sponges) did not insert into the R2 target site. Some of these proteins lacked a Myb domain, and these had a high sequence homology to Utopia non-LTR retrotransposon proteins (Table S4), which are often site-specific for U2 small nuclear RNA (snRNA) genes [21]. We classified retrotransposons as Utopia rather than R2 if the predicted protein lacked the hallmark R2 Myb domain (Fig. 2a, Additional file 1: Fig. S1a, Table S4). The subset of R2-homologous Porifera retrotransposon proteins that harbour a predicted Myb domain and therefore are included in Fig. 1b have variable flanking sequences that are not the canonical R2 28S rDNA insertion site (Fig. 2b, top branch). We classified these as non-site-specific R2-homologous retrotransposons (Tables S4-5).

### The number of N-terminus ZnF is not a defining characteristic of evolutionary lineage

For additional analysis, we translated our newly curated R2 ORFs. R2 ORFs are non-canonically translated and can lack an initiating methionine [8, 23]. We narrowed down our R2 dataset by filtering out any R2 with an ORF less than 900 amino acids, as this is below the typical length of even the shortest previously identified R2p. This reduced the generated dataset to 180 R2 sequences. We trimmed each ORF to include 10 amino acids N-terminal to the most N-terminal ZnF and used this amino acid sequence for tree-building. Several observations emerged from this analysis.

First, R2 clustering was discordant with species phylogeny overall (Fig. 1a) when the tree is rooted with a Group II intron protein (Fig. 1b). The R2 sequences fall predominantly into two major branches (Fig. 1b, ring with two grey arcs). Within the 3-ZnF branch (Fig. 1b, lower grey arc), for the most part, R2 phylogeny conforms to host phylogeny. The exception is the 2 groups of Echinodermata R2 split by Mollusca R2s. In the second major branch, there are R2s with 4 to 1 ZnF (Fig. 1b, upper grey arc), with the majority having 1 ZnF. The early-branching R2 from this group occur in species of Ctenophora, Mollusca, Tunicata, and Arthropoda, and the Porifera R2-homologous retrotransposons fall within this cluster. The ctenophore R2 from *B. forskalii* encodes a protein with possibly 4 N-terminal ZnFs (Fig. 1b, inner ring red colour, Additional file 2: Fig. S2) and deviates also in that the flanking target-site rDNA was upstream of the canonical R2 nick site by 11 base-pairs (Additional file 3: Fig. S3). In a phylogenetic tree with all partial R2 ORFs in addition to full-length ORFs, the overall R2 phylogeny remains the same (Additional file 4: Fig. S4.1 rooted with ORF2 protein from human non-LTR long interspersed element 1 (L1)). The same result is observed when constructing the tree with sequence from the RT domain only versus ORFs greater than 900 amino acids (Additional file 4: Fig. S4.2–3, rooted with human L1). Curiously, the ctenophore R2s with 2–4 ZnF consistently clustered with 1 ZnF R2, and by AlphaFold prediction, the ZF1-Myb makes DNA contacts more similar to 1-ZnF R2p than to other 3-ZnF R2p (Additional file 1: Fig. S1b).

Despite greatly expanding the number and species distribution of R2s, we found only a few with two ZnFs (Fig. 1b, inner ring orange colour). By previous categorisation, these would be designated as B or C clade. Previously, based on phylogenetic clustering, B clade was assigned to R2s in Arthropoda, and C clade was assigned to R2s in Platyhelminthes, a bitterling fish, and a snake [7, 12, 13]. In our expanded R2 dataset, several R2s from Chondrichthyes (sharks and stingrays) with ZnFs that match C clade are clustered with 1 ZnF R2s (Fig. 1b) and with previously annotated C clade R2s, but not B clade (Additional files 5–6: Figs. S5-S6) [7, 12]. Also surprisingly, most identified Ctenophora R2s have a ZnF configuration that matches the B clade (Additional file 7: Fig. S7), but they do not cluster strongly with previously annotated B clade R2s (Additional file 6: Fig. S6). The scattered distribution of R2s with 2 ZnF suggests that these domain architectures can potentially arise independently of lineage ancestry rather than as vertically inherited clades of R2.

The distinction between two successful R2 lineages was supported by constructing phylogenetic trees with sequences from individual domains of R2p beyond the RT domain: trees based on ZnF1 and Myb, RLE (endonuclease), or Thumb-zinc knuckle domain sequences all resulted in a similar tree topology (Additional file 8: Fig S8.1–3). Because N-terminal domain architecture does not define evolutionary proximity, we henceforth refer to the two major phylogenetic branches of R2 as lineages A (Fig. 2c) and D (Fig. 2d) to match the most common number of N-terminus ZnFs to their previous clade A and D designations: lineage A corresponds to clade A R2s, whereas lineage D encompasses previously identified R2s of clades B, C and D. We observe a third, more ambiguous branch near the root of the lineage D tree comprising Arthropoda R2s with 3 ZnFs (Fig. 2d: *M. septendecula*, *A. carantonica*, *L. quadrifasciata*, and *M. religiosa*). This branch of R2s appears to cluster variably with lineage D or A when using different protein domains for tree building (Additional file 8: Fig S8.1–3). The variability persists even when the tree is rooted with human L1 using either the R2 RT domain or the full R2 ORFs (Additional file 4: Fig. S4.2–3).

Continuing clockwise starting from *T. miniatum* in Fig. 2d, additional Arthropoda and some Tunicata R2p group in the lineage D branch with high support values despite structural predictions suggesting that some of these R2p have a co-folded ZnF3:2 (Fig. 2b), a structural property of lineage A R2p (see below). The remainder of the cluster has unambiguously lineage D R2p, including several with domain architectures that would place them in previous B or C clade (Fig. 2d) but are more accurately described as B or C domain architecture. Independent of the number of ZnFs in an ancestral R2p, phylogenetic relationships amongst our expanded inventory of R2s suggest that ZnFs can be lost and gained more dynamically than had been anticipated.

### Structural variations in N-terminus ZnFs of lineage D and A R2p

AlphaFold prediction of 3D structures of newly annotated R2p revealed a consistent lineage A feature: interdependently folded ZnF3 and ZnF2 (henceforth, co-folded ZnF3:2) (Fig. 2e). We noticed a predicted presence of this structural feature, although inconsistently, in the non-site-specific R2-homologous Porifera retrotransposon proteins (Fig. 2b, Additional file 1: Fig. S1c). Nonetheless, when a ZnF3:2 co-fold is predicted, for example in the case of the *E. muelleri*−1 non-site-specific R2-homologous protein, the fold is superimposable with ZnF3:2 from a lineage A R2p such as from the turtle *P. megacephalum* (Fig. 2e). The *P. megacephalum* (PlaMe) R2p ZnF3:2 co-fold has been confirmed by cryo-electron microscopy [22]. Intriguingly, we did not find similarly co-folded ZnFs in other proteins by protein structure database searches (see Methods). However, we found that AlphaFold predicted similar co-folded ZnFs in some Utopia proteins (see Utopia ZnF2:1 compared to R2 ZnF3:2 in Additional file 9: Fig. S9), suggestive of shared phylogeny (see Discussion). The long-branch Arthropoda and Tunicata R2p that cluster near Porifera R2-homologous proteins and share ambiguous tree placement (adjacent to lineage D in Fig. 1b and Additional file 4: Fig. S4.1 or lineage A in Additional file 4: Figs. S2-3) either are predicted to have co-folded ZnF3:2 (the tunicates *T. miniatum* and *A. mentula*, Fig. 2b) or not (the arthropod *A. biguttatus,* and 4 additional arthropods; Table S6), the latter 5 possibly due to gain of an atypically long linker between ZnF3 and ZnF2. The ctenophore *B. forskalii* R2p that harbours 4 putative N-terminal ZnFs also lacks the ZnF3:2 co-fold; instead, it has distinctively short linkers between the 3 most N-terminal ZnFs (Additional file 1: Fig. S1.d). These variations complicate the definition of an evolutionary origin for lineage A (see Discussion).

Beyond differences in the number and folding of ZnFs, R2p have additional structural variations in their N-terminal domains (see Fig. 2a colour coding summary). In lineage A R2p, we observed differences in the ZnF3 sequence. Aves R2 ZnF3 often starts with CPCC, which still can form a typical ZnF. However, R2s from some avian species do not have this feature (Fig. 2c, see ZnF3 colour code). Strigiformes (owls) in particular (*P. badius, O. scops, S. o. caurina*) do not have the CPCC ZnF3. Even more structural distinction was evident in lineage D R2p (Fig. 2d; see Fig. 2a colour coding summary). Unusual spacing of cysteines occurred at the start of ZnF1, with CxC instead of CxxC. CxC was detected in snakes, fishes, and flatworms, and CxxxC was detected in arthropods (Fig. 2d, see ZnF1 colour code). Most remarkably, a Myb domain duplication (Fig. 2a, annotated as Myb2) was discovered in a cluster of Actinopterygii fish species from the family Cyprinidae (Fig. 2d). This diversification parallels the greater number of sub-lineages in lineage D than lineage A, as visualised in the greater lineage D tree departure from expectation based on host organism phylogeny (compare the outer arcs in Fig. 2c, d).

### Different RT motif adaptations in lineage D versus A

**Fig. 3:**
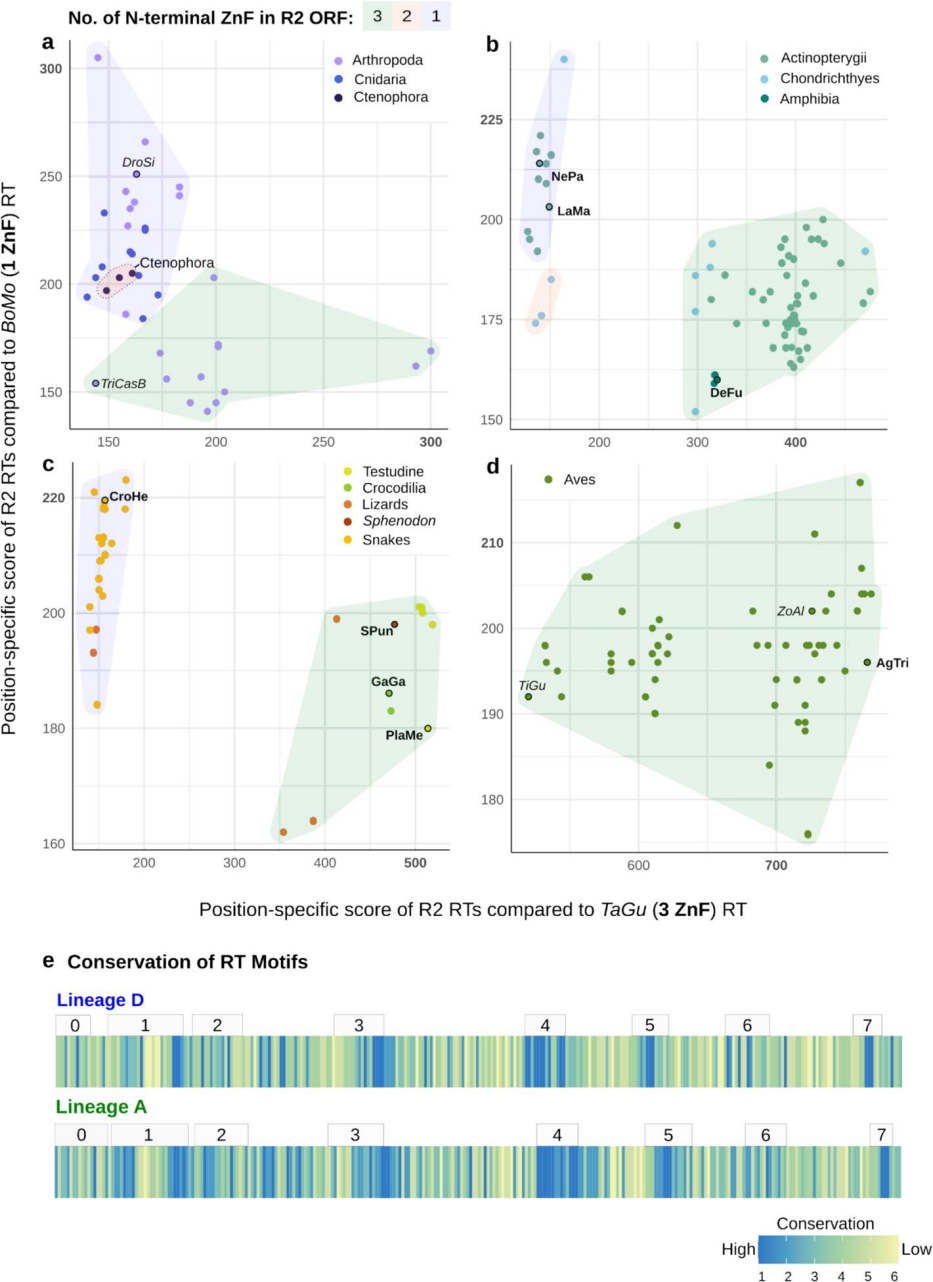
R2p RT-domain differences within and between lineages. PSI-BLAST derived position-specific scoring matrix scores of rDNA-specific R2 RTs compared to BoMo (lineage D) and TaGu (lineage A) RT domains. R2p selected for activity testing are circled in black and in bold font. R2p tested in previous studies are in italics: DroSi (*D. simulans*), TriCasB (*T. castaneum*), TiGu (*T. guttatus*), and ZoAl (*Z. albicollis*). ZoAl R2 sequence was reconstructed from short-reads (see Methods). DroSi, TriCasB, and TiGu are previously characterised sequences [6, 27]. Shading in each panel corresponds to the number of N-terminus ZnFs in the complete R2p sequence. R2 RTs from major classes of animals are shown: (**a**) Arthropoda, Cnidaria, and Ctenophora; (**b**) Amphibia, Actinopterygii, and Chondrichthyes; (**c**) Reptilia; and (**d**) Aves. R2p with an atypical number of N-terminus ZnFs (> 3) were excluded. **e** Conservation of individual residues in the RT domain for lineage A and D R2p. Conservation of residues is denoted with a lower score (1–3), and more rapidly evolving residues are denoted with a high score (4–6). RT motifs from 0 through 7 are annotated

As an additional approach to explore R2 evolution, we used position-specific scoring matrices (PSSM) to compare translated RT domains spanning the newly identified R2p RT motifs from 0–7 [24, 25] to representative R2p known to be biochemically and biologically active: lineage D R2p RT from the silk moth *B. mori* and lineage A R2p RT from the zebra finch *T. guttata* [6, 7]. PSSM scores (Fig. 3a-d; *B. mori* as BoMo on each y-axis and *T. guttata* as TaGu on each x-axis; see also Additional file 10: Fig. S10.1–5 for detailed annotations) reflect common functionality rather than only sequence identity [26]. A PSSM calculates the match score of an alignment based on the likelihood of each amino acid at each alignment position. The score shows how likely a residue is to be functional based on the alignment. Residues that are conserved and align with the R2p from *B. mori* or *T. guttata* get higher scores. Thus, we would expect a lineage D RT to cluster with BoMo, and vice versa with Lineage A RTs and TaGu. Comparing R2p from early-branching phylogenetic groups (ctenophores, cnidarians, and arthropods), there is as much divergence within grouping by ZnF number as between R2p with different numbers of ZnF (Fig. 3a, see ZnF colour key for background shading and symbol colour key for species). In contrast, amphibian and fish R2p are clustered in correlation with the number of ZnF (Fig. 3b) as are reptilian R2p (Fig. 3c). Newly identified avian R2p [12] are only from lineage A, as was true for previously reported avian R2p, but two subclusters are observed (Fig. 3d), which are largely delineated by whether the R2 is from a passerine (Additional file 10: Fig. S10.1). Passerine R2p include ZoAl from *Z. albicollis* and TaGu, both of which support PRINT transgene insertions in human cells. Non-passerines include R2p from tinamou *T. guttatus*, TiGu, which does not support efficient transgene insertion in human cells [6]. We note that PSSM-based analysis is intended to support relative functional comparisons among characterised R2p but may underrepresent divergent and/or early-branching RT architectures.

We also compared R2p RT domain sequences using a site-specific rate algorithm [26], to infer the evolutionary rate of change of individual amino acid side chains in lineage D versus lineage A (Fig. 3e; Additional file 11: Fig. S11). A highly conserved site is given a lower score (1–3, blue), whereas a more variable site is given a high score (4–6, yellow). Within lineage A or D, different patterns of sequence conservation were evident (Fig. 3e). We speculate that the distinct pattern of conserved residues in lineage A compared to lineage D R2p RT domains indicates that RT domain sequence co-evolved with changes in N-terminal domain architecture (see Discussion).

### Co-existence of R2 lineages in phylogenetic groups and individual genomes

In addition to expanding the known phylogenetic breadth of R2 perpetuation, the new R2 inventory also expanded the known co-occurrence of lineage A and lineage D R2s within phylogenetic groups. For example, in addition to arthropods [11, 12], other species also have R2 from both lineage D and A: Squamata (lizards), Actinopterygii, Chondrichthyes, and Mollusca (Fig. 1b, pairs of circled numbers show these instances of lineage A and lineage D phylogenetic co-occurrence). Surprisingly, within these phylogenetic groups, we found several species with full-length R2s from different lineages, as previously described for arthropods [11, 12]. These include species of Actinopterygii fish (*L. marequensis*, *B. barbus*), Chondrichthyes sharks (*S. tigrinum*), and Tunicata sea squirts (*A. mentula)* (Table S7). Therefore, it is not as rare as we had anticipated that R2s from multiple lineages co-exist in an organism despite competition for the same target sites.

### Survey of R2p endonuclease and TPRT activities across diverse species

**Fig. 4:**
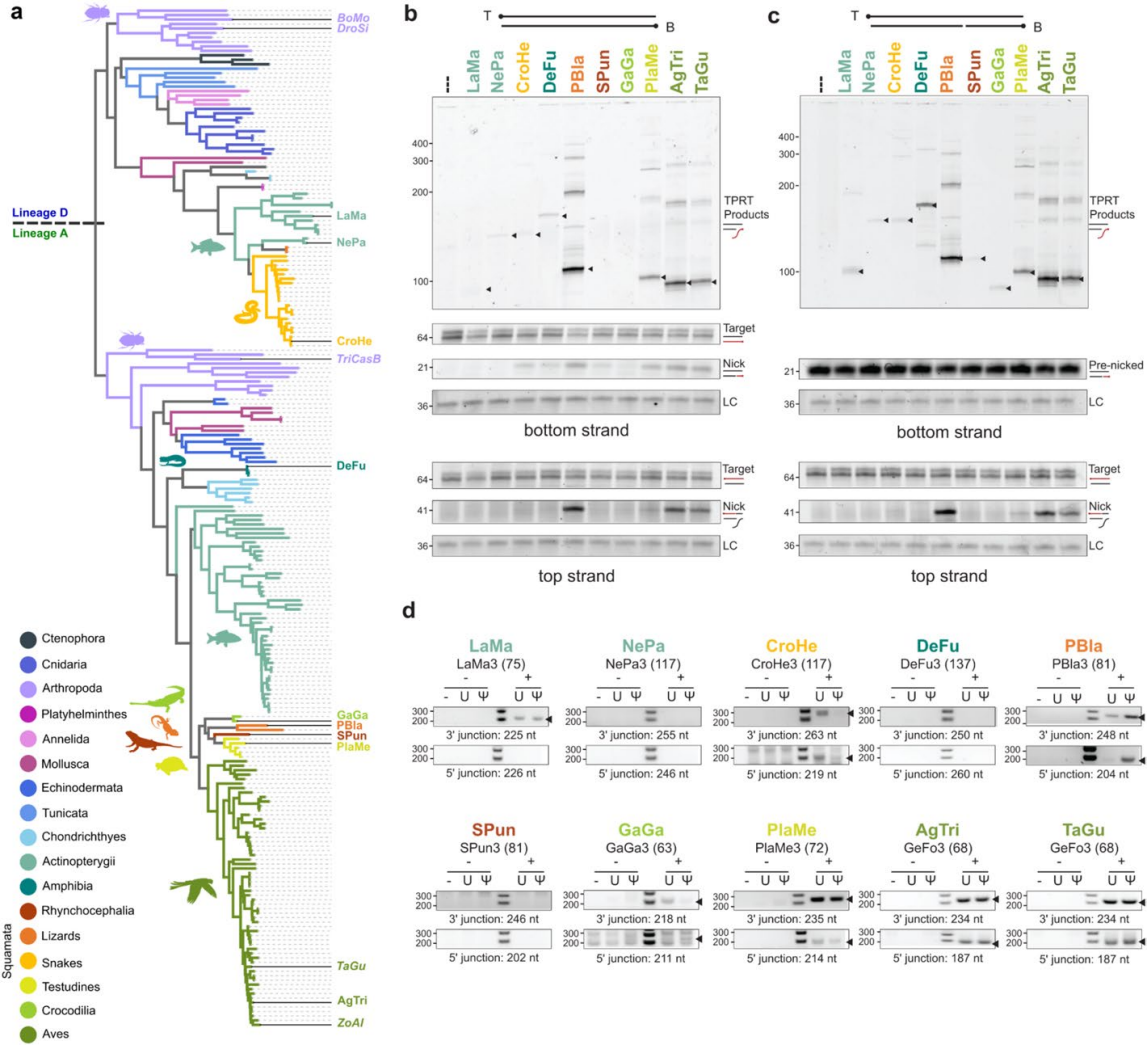
Activity across diverse R2p. **a** Phylogenetic tree of R2p RT domains. R2p annotated at their corresponding phylogeny branch position were selected for activity testing. R2p in italics have been tested in previous studies [6, 27]. **b**, **c** In vitro assays using either intact (**b**) or pre-cleaved (**c**) target-site oligonucleotide substrates. Protein expression was confirmed by immunoblot (SI Fig. 13). Expected TPRT product sizes for initial cDNA synthesis products are denoted with a black triangle; note that longer products can form by template-jumping, due to excess RNA in the reactions [6]. Bottom-strand and top-strand visualisation gels were run separately. All panels for top or bottom strand were cropped from the same gel, but target-site DNA intensity was adjusted independently of product intensities. LC indicates the loading control for product recovery with precipitation and gel loading. **d** Detection of 3' and 5' rDNA-insertion junctions by PCR. Detection of an rDNA-inserted transgene 3’ junction indicates successful cellular TPRT, while detection of a transgene 5’ junction indicates stable insertion. Sizes of truncated 3’UTRs used for this assay are in parentheses. Expected product sizes are listed below each gel and those expected products are denoted with a black arrowhead

Genome-embedded copies of retrotransposons are often incapable of mobility, which brings into question whether divergent R2p from our expanded phylogeny retain the biochemical activities essential for mobility. We selected newly identified R2p with apparently intact RT motifs that had different architectures of N-terminal domains to sample for activity, focused on phylogenetic groups beyond the Arthropoda R2s that have been best studied (Fig. 4a, Additional file 12: Fig. S12). Our final choices included two lineage D fish R2p: LaMa (*L. marequensis*, Largescale yellowfish) with a Myb domain duplication, and NePa (*N. papilliferus*, killifish) with CXC ZnF1. We chose lineage A amphibian R2p DeFu (*D. fucus*, Northern dusky salamander) as a representative for the first identification of amphibian R2s, and among reptilian R2s we included a lineage D snake R2p CroHe (*C. o. helleri*, Southern Pacific Rattlesnake) and several lineage A R2p: PBla (*P. blainvillii*, Blainville's Horned Lizard), SPun (*S. punctatus*, Tuatara), GaGa (*G. gangeticus*, Gharial crocodile), and also PlaMe (*P. megacephalum*, Big-headed turtle) subsequently used for structure determination [6, 22]. As another avian R2p representative, we tested AgTri (*A. tricolor*, Tricoloured blackbird).

The first functional test for our selected R2p used purified recombinant proteins for in vitro assays of 28S rDNA target-site nicking and TPRT. As a standard for comparison, we used the relatively well-characterised TaGu protein with robust TPRT and site-specific gene insertion capability in human cells [6, 22]. As done previously, R2p was expressed from a plasmid synthetic ORF by transient transfection of human HEK293T cells and purified by FLAG antibody resin [6]. Also as done previously, a template RNA was in vitro transcribed that harbours the species-matched R2 3’ UTR 3’ end and a 3’ tail complementary to the nicked primer (Table S8) [6]. Annealed target-site oligonucleotides had 5’-end dye labels to detect first-strand (bottom strand) nicking and cDNA synthesis or second-strand (top strand) nicking (Fig. 4b, c, schematics at top). We tested R2p activities both with intact target-site duplex (Fig. 4b) and also with bottom-strand “pre-nicked” target-site duplex (Fig. 4c) to evaluate TPRT with or without the requirement for first-strand nicking, respectively.

Several recombinant R2p demonstrated robust first-strand nicking and TPRT comparable to the positive-control TaGu R2p, including lizard PBla, turtle PlaMe, and avian AgTri (Fig. 4b). Fish LaMa and NePa, snake CroHe, and salamander DeFu had weak activity (Fig. 4b). LaMa and DeFu TPRT activities were improved using pre-nicked target site DNA, and weak TPRT by gharial GaGa and Tuatara SPun became detectable (Fig. 4c). Second-strand nicking activity tracked with first-strand nicking activity (Fig. 4b, c, lower panels). Overall, within this sampling, we conclude that most tested R2p have nicking and cDNA synthesis activities. However, weak first-strand nicking limits TPRT by LaMa, DeFu, GaGa, and SPun, evidenced by increased TPRT with pre-nicked target-site duplex.

Our second functional test used a version of the PRINT protocol for R2p-mediated transgene insertion [6, 28]. In brief, a proliferatively immortalised RPE-1 human primary cell line was co-transfected with mRNA encoding an R2p and a template RNA with the species-matched R2 3’ UTR 3’ end and primer-complementary 3’ tail. R2p expression levels were compared by immunoblot (Additional file 13: Fig. S13). We detected successful genome insertion at the R2 28S rDNA target site by PCR. Again we used TaGu as a positive control, and we compared template RNAs with native uridine or the 100% pseudouridine substitution that enhances TaGu-mediated genome insertions [6, 28]. Template RNA transfection without R2p mRNA was the negative control.

Across the R2p panel, successful cellular TPRT was detected by transgene 3’ junction formation when R2p mRNA and template RNA were co-transfected (Fig. 4d, top panel of each set; first sample in each panel is parental cells alone, and the next two are template RNA without R2p mRNA as negative controls). Efficiency of transgene insertion generally mirrored in vitro TPRT activity on intact target-site duplex (Fig. 4b). PBla, PlaMe, and AgTri were most active; NePa, DeFu, and SPun appeared not active in cells, while LaMa, CroHe, and GaGa were weakly active (Fig. 4d). We note that R2p without function detected using PRINT in human cells may nonetheless be functional in native organism context. Curiously, CroHe and GaGa preferred template RNA with uridine rather than pseudouridine, which we speculate could result from a modified-nucleotide influence on RNA folding or binding to R2p. The RNA structure bound by R2p has changed over evolution, as inferred from divergence of R2 3’ UTR sequence and predicted folding (Table S8). This divergence could underlie different levels of 3’UTR RNA structural disruption by substitution of uridine with pseudouridine. We also detected rDNA-insertion 5’ junctions (Fig. 4d, bottom panel of each set) that compared to the TaGu positive control were robust for PBla and AgTri, with PlaMe, CroHe, and GaGa also showing detectable levels of stable transgene insertion compared to the negative controls (first 3 samples of each panel). We conclude that when assayed in human cells, many tested R2p retained the functionality for gene insertion that is essential to native retrotransposon mobility.

## Discussion

**Fig. 5:**
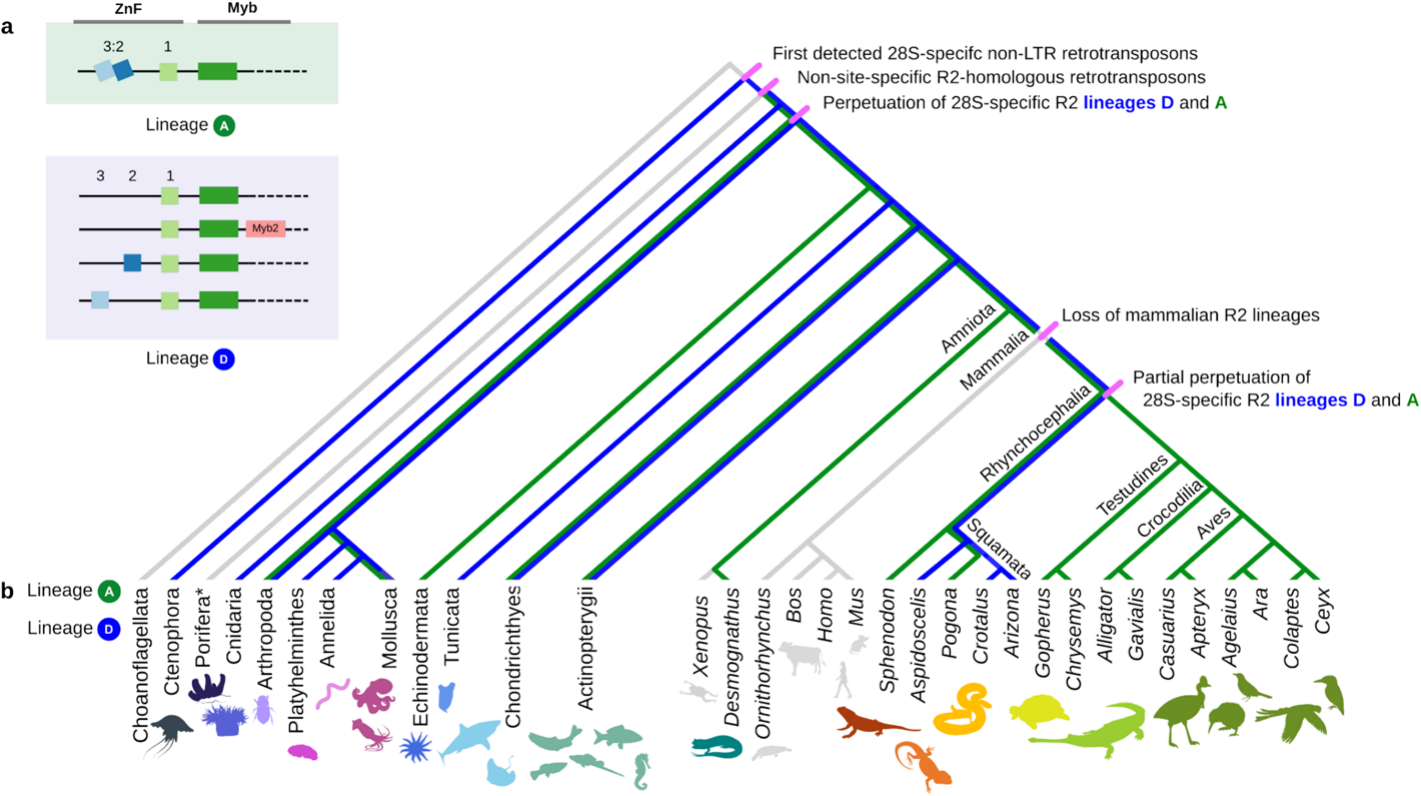
Summarising observed R2 inheritance in Metazoa. **a** Key outlining different ZnF and Myb configurations present in R2 lineages. Lineage A has co-folded ZnF3:2, while lineage D generally does not. **b** Superimposition of persistence of R2 lineages D and A in key phyla across Metazoa. Branch lengths are not on an evolutionary time scale. (*) Porifera non-site-specific R2-homologous retrotransposons are not lineage D or A

In this study, we curated R2 retrotransposons from 300 species across 12 phyla. The wealth of genome sequence compiled in long reads, short reads, and genome assemblies enabled our goal of expanding the curation of full-length R2s. The R2 inventory from this work broadens the appreciation of R2 diversity. We demonstrate that R2s have persisted steadily over Metazoan evolution in two major lineages, D and A (Fig. 5 summarises the overall inheritance patterns of lineages D and A across animals). The sheer diversity in terms of species where R2s are found, as well as the diversity of R2p structural variation, were underappreciated from previous studies and even by our expectations at the launch of this work.

We used biochemical assays to evaluate the activities of R2p across a spectrum of phylogenetic groups and domain architectures, focusing on groups other than those previously sampled. The parallel assay conditions for each R2p do not attempt to capture physiological differences in the organisms, and therefore, negative findings do not necessarily indicate a lack of biological function. Despite this departure from the physiological context, we demonstrate that the fish LaMa lineage D R2p with an entirely unanticipated Myb domain duplication retains function for 28S rDNA insertion. How the evolutionary innovations of N-terminal domain architectures and ZnF sequence influence insertion efficiency and the fidelity of site-specific insertion remain to be investigated with more directed structure/function assays.

In agreement with previous studies, we observed that the host tree and R2 tree are incongruent. In the course of our analyses it became clear that R2 clade designation by number of ZnF does not sufficiently classify the sub-groups of R2. Overall, we postulate that two main branches of R2 emerged and diverged early in Metazoan evolution: lineages D and A. Earliest emergence of lineage D is consistent with clustering of ctenophore R2s with lineage D (Fig. 1). Also, ctenophore R2p are predicted to share the BoMo lineage D configuration of ZnF1-Myb contacts with target site (Additional file 1: Fig. S1) that have been visualized at atomic resolution [29]. We propose that widespread lineage D survival was founded on an ancestral R2p with ZnF1-Myb specific for the R2 rDNA target site, which in several sub-lineages gained additional ZnF and/or Myb domains. Even considering only lineage D R2p with streamlined N-terminal ZnF1-Myb alone, several sub-lineages arose that maintained independent mobility, especially notable in Cnidaria and Tunicata (see Fig. 2d).

In comparison to lineage D, lineage A has few, if any, sub-lineages within our sampling. We propose that lineage A distinction from lineage D succeeded due to the gain of a unique combination of N-terminal ZnFs. Lineage A differs from lineage D in ZnF1-Myb positioning on upstream DNA (Additional file 1: Fig S1), raising the possibility that lineage D ZnF1 was replaced by 3 ZnFs from an R2-homologous protein with co-folded ZnF3:2. The TaGu and PlaMe ZnF3:2 co-fold [22] can be modelled in structural alignment with the ZnF2:1 of particular Utopia proteins (Additional file 9: Fig. S9), which raises the possibility that lineage A evolved from lineage D by capture of N-terminal domains from a Porifera R2-homologous protein. We speculate that the R2 lineage A co-folded ZnF3:2 provides an evolutionary advantage at least in part by enhancing and coordinating the recognition and relative positioning of template RNA and target-site DNA [22, 27]. The distinct signatures of lineage D and A RT-domain motifs (see Fig. 3e) suggest that many RT domain features co-evolved with changes in N-terminal domain architecture. One clear example is the change in RT motif 6a contacts to target site DNA, which are sandwiched between contacts made by ZnF1 and Myb and are different in lineage D BoMo versus lineage A TaGu and PlaMe [22, 27]. Investigation of R2 features that contribute to lineage A success is a high priority for optimisation of avian R2p utility for transgene insertion into human genomes [6].

The apparent patterns of R2 lineage D and A distribution (Fig. 5) suggest that R2s are not continuously maintained. This can be explained by differential retention of shared ancestral lineages without invoking horizontal transfer (HT) [30, 31]. While potential R2 HT has been suggested for *Bacillus* stick insects facilitated through their complex reproductive systems [32], we did not identify hallmarks of recent HT (e.g. unusually high sequence similarity between divergent species; Table S9 shows percentage identity matrix between R2p sequences). Although we do not exclude the possibility of HT, our R2 tree can be explained more simply by stochastic loss of lineages. In the future, other explorations could be used to investigate HT in early Metazoan evolution.

Even with our expanded set of annotated R2s, the nature of the R2-precursor non-LTR retrotransposon remains unclear. We hypothesise that R2 emerged from a non-LTR retrotransposon with some degree of target site specificity, which subsequently adapted to recognition of the 28S rDNA locus. Porifera R2-homologous retrotransposons are peculiar as they have an R2p domain architecture but have flanking sequences more like those of Utopia retrotransposons. Porifera R2-homologous proteins cluster near the root of the R2 phylogeny, with weak clustering within lineage D when the tree is rooted with a Group II intron protein or a human L1 ORF2 protein (Figs. 1b and SI Fig. 4.2–3). Inclusion of RT domains from the R2-homologous proteins in the PSSM analysis failed to place them as closely related to either lineage A or D R2p RT domains (Additional file 10: Fig. S10.5).

In one possible model of R2 origin from a Utopia retrotransposon, or a common progenitor of both R2 and Utopia, Myb domain acquisition occurred prior to the gain of specificity for 28S rDNA, reflected in the R2-homologous retrotransposons from Porifera. The current perspective is that Ctenophora are the sister group to all other animals, rather than the previous assignment of this role to Porifera [33]. Yet recent analyses place Porifera as the earliest-branching animal lineage [34], and so the placement of Ctenophora versus Porifera remains a debated issue in deep metazoan phylogeny. We propose two scenarios to accommodate this ambiguity. If Porifera is considered to be the sister outgroup to other animals, we speculate that the origin of evolutionarily successful rDNA-specific R2 occurred prior to the divergence of Ctenophora, with the establishment of lineage D. The Myb-domain-containing but not rDNA-specific R2-homologous proteins in Porifera could have persisted in early animal genomes long enough to contribute N-terminal domains to a precursor of lineage A, which gained its evolutionarily successful domain configuration after the divergence of Cnidaria.

If instead Ctenophora are the sister group to all other animals, we suggest a model for R2 evolution in which rDNA site-specificity was acquired early, before divergence of Ctenophora R2 and Porifera R2-homologous proteins. R2 use of its rDNA target site would be favourable for retrotransposon perpetuation, due to high rDNA copy number and sequence conservation at the R2 target site in particular. However, tandemly repeated gene arrays, including rDNA, undergo non-allelic homologous recombination, which can cause duplication and deletion of sequences [35]. Ribosomal DNA arrays are prone to copy number variation and, therefore, there could be potential loss of rDNA-embedded R2 in Porifera [36]. Whether there is a widespread R2 contribution to rDNA copy number maintenance, as demonstrated recently in *D. melanogaster* [37], remains to be explored in future work.

## Conclusions

Together, our expanded survey significantly broadens the known phylogenetic range and functional diversity of R2 retrotransposons across Metazoa. We show that R2s are more widespread than previously appreciated and appear to form two major lineages, A and D. These lineages are not defined solely by N-terminal ZnF architecture, but instead reflect a more complex history of domain gain, loss, and structural innovation, including lineage-specific features such as the co-folded ZnF3:2 in lineage A. Functional assays demonstrate that many newly identified R2 proteins retain endonuclease and TPRT activities, highlighting their ability to mobilise despite extensive structural and sequence divergence across Metazoan evolution. Collectively, these findings establish a comprehensive framework for understanding R2 evolution and provide a foundation for future studies to explore their biological properties and potential biotechnology applications.

## Methods

### R2 discovery pipeline

We conducted online BLASTN + and TBLASTN searches using reference R2 query sequences (Clade A, B, C, and D R2s were used initially, and then species-specific queries were used) against the genome assembly of any species of interest (sensitive search, word size = 7) [38]. If the top hit was a full-length R2 flanked by 28S rRNA, the sequence was annotated as an R2. If the top hits were truncated or appeared to be misassembled, and both long and short-read data were available, we downloaded the long and short reads from the SRA database (https://www.ncbi.nlm.nih.gov/sra). See Additional file 14 or GitHub (https://github.com/Noz98/R2-Retrotransposon-Workflow) for full parameters and workflow. If there were no significant hits, we did not investigate that species further.

Using long-read sequences, a local sensitive BLASTN + search was performed with reference R2 queries. We processed the short reads through FastQC, and adaptors were removed with fastp (http://www.bioinformatics.babraham.ac.uk/projects/fastqc) [39]. Long-read sequences containing R2s were error-corrected with short-reads using Ratatosk (default) [40]. We checked the quality of error correction with IGV [40, 41]. R2s were re-extracted from the error-corrected long reads using a local sensitive BLASTN +. Top hits with flanking 28S rRNA target sites were annotated as full-length R2s. If the sequences were truncated (i.e. have a truncated ORF), we annotated them as partial-length R2s. We translated R2 ORFs using ExPASY and NCBI ORF Finder [42] (https://www.ncbi.nlm.nih.gov/orffinder/). We used NCBI Conserved Domain Search to identify key domains [43]. We searched our curated R2 against the Repbase repeat masking function to confirm that we had correctly annotated our sequences [44]. Finally, we generated a percentage identity matrix based on nucleotide sequences to visualise R2 sequence similarities with MUSCLE [45]. R2 sequences from *B. mori, T. guttata*, *T. guttatus*, *T. castaneum*, *N. vitripennis*, *L. polyphemus*, *D. mercatorum* were used from previous studies [12, 17, 46]. We note that R2s from *C. tenuis* (kilifish) and *H. berthatutzae* (stingray) are unusually similar and at least one could be a contaminant of genome sequencing.

### Phylogenetic tree-building

We aligned R2 ORFs that were longer than 900 amino acids with MAFFT v7.490 (Auto model selection) and trimmed the alignment with ClipKIT [47, 48]. We manually checked each alignment generated with AliView [49]. We used IQTree v2.0.3 for tree reconstruction with 20 maximum likelihood trees and 1000 bootstraps [50]. ModelFinder was used to obtain the best-fit model for tree building (-m MFP) [50, 51]. We reduced the number of Amphibia R2s in all phylogenetic trees from 37 to 3 representative sequences. We did several iterations of tree building to test topology based on domain partitions (Partitioned analysis for multi-gene alignments) with MFP + MERGE: 1) complete R2 ORF, 2) RT, 3) ZnF-Myb, 4) RLE, and 5) Thumb-ZnF [50, 52]. To check our tree topology for reliability, we used RaxML [53] (Additional file 15: Fig. S15). We visualised and edited our tree using iTOL: Interactive Tree of Life and TVBOT [53–55]. We used a Group II intron protein from *T. vestitus* (cyanobacteria) as the outgroup (Fig. 1) and further compared trees using human long interspersed element 1 ORF2 protein [56] as an outgroup.

We used TimeTree to build a species tree representing the major classes of organisms with R2s [45, 57]. This included the following: Amphibia, Arthropoda, Cnidaria, Ctenophora, Echinodermata, Actinopterygii, Chondrichthyes, Mollusca, Platyhelminthes, Porifera, Reptilia (Aves, Rhynchocephalia, Squamata, Testudine) and Tunicates.

### Protein structure prediction and sequence comparison

For protein structure prediction, we used the AlphaFold3 web server (https://alphafoldserver.com/) [58, 59]. Predictions were done for both protein alone and protein plus target-site DNA oligonucleotides. We used ChimeraX [60] v1.8 for structural analysis and alignments, including the protein fold visualisations shown. To search for structural similarity to the lineage A co-folded ZnF3:2, we used PlaMe ZnF3:2 as a query to perform a Structure Similarity Search following the recommended pipelines by RCSB.org (https://www.rcsb.org/) to query for both structures within the Protein Data Bank (PDB) and those available in other public data resources (e.g. AlphaFold or RoseTTAFold predictions). We manually examined polymer entities that ‘matched’ the query, which in all cases lacked the R2p ZnF3:2 co-folding. Cofolded ZnFs that structurally matched lineage A cryo-EM structures were only detected when we individually predicted the protein structures of apparent full-length Utopia elements described previously (Additional file 9: Fig. S9) [12, 21].

We used the BLAST + position-specific scoring matrix to calculate relative similarity [61]. Each predicted R2p RT was incorporated into a PSI-BLAST database and queried using BoMo and TaGu RT sequences previously validated to be active R2p [6, 9]. The alignment bitscores were used to visualise the similarity of each R2 to the lineage D and A representatives in Fig. 3a-d (also see Additional file 10: Fig. S10.1–5). To infer site-specific rate of evolution for R2p amino acids (Fig. 3e), we used IQTree (LG + R6, –rate) [50] (IQTree; http://www.iqtree.org/doc/Advanced-Tutorial). We extracted the numerical value for the conservation of each residue and mapped it against the RT sequence. We then annotated the position of RT motifs (0 to 7) [25].

### Cell culture

RPE-1 hTERT cells were grown in DMEM/F12 (Gibco), and HEK293T cells were grown in DMEM (Gibco). All media was supplemented with 10% fetal bovine serum (FBS; Seradigm) and 100 μg/mL Primocin (InvivoGen). Cells were cultured at 37 °C under 5% CO2. All cells were tested for mycoplasma contamination, and human cell lines were validated by short tandem repeat profiling (Promega, catalogue no. B9510).

### Protein expression, purification, and detection for biochemical assays

HEK293T cells were transiently transfected with pcDNA3.1 plasmids with synthetic ORFs encoding proteins N-terminally tagged with a single FLAG peptide (Table S8) used for affinity purification as previously described [6]. In brief, cell pellets were resuspended in HLB (20 mM HEPES pH 8.0, 2 mM MgCl2, 200 μM EGTA, 10% glycerol, 1 mM DTT, 0.2% protease inhibitor cocktail (Sigma, catalogue no. P8340), 1 mM PMSF). After 5 min on ice, cells were lysed by three cycles of snap freezing in liquid nitrogen and thawing in a room-temperature water bath. Lysate was brought to 400 mM NaCl, gently vortexed, and placed on ice for an additional 5 min; then, NaCl was lowered to 200 mM and 0.2% NP-40 was added prior to centrifugation. Clarified extract was incubated with FLAG resin (Sigma, catalogue no. A2220) at 4 °C for 2 h. Resin was washed with the same buffer and protein eluted with 50 ng/μL 3xFLAG peptide (Sigma, catalogue no. F4799) at room temperature for 1 h. The slurry was aliquoted, snap-frozen, and stored at −80 °C. Immunoblots used anti-FLAG antibody (Sigma, catalogue no. F1804, 1:3000) and Alexa Fluor 680 goat anti-mouse secondary (Thermo Fisher, catalogue no. A21057, 1:2,000) detected by LI-COR Odyssey.

### RNA synthesis

R2 mRNAs and template RNAs were synthesised using T7 RNA polymerase as previously described [6, 28]. In brief, mRNAs were made using plasmid template linearised with BbsI-HF (NEB) for 4 h at 37 °C, purified, and transcribed with AG Clean Cap (TriLink, catalogue no. N-7113) per the manufacturer’s protocol, with complete substitution of uridine with N1-methylpseudouridine (TriLink, catalogue no. NC1443155). The mRNA UTR sequences are from the BioNTech COVID-19 vaccine mRNA as previously in this work, followed by a plasmid-encoded 30-nucleotide poly-adenosine tail [6]. Transcription reactions were incubated at 37 °C for 2 h, followed by the addition of 2 μL RNase-free DNase I (Thermo Fisher, catalogue no. FEREN0521). Product RNA was purified by desalting with an Illustra Probe-Quant G-50 Micro Column (Cytiva, catalogue no. 28903408) followed by phenol–chloroform-isoamyl alcohol (PCI; Thermo Fisher, catalogue no. BP1752I-100) purification and precipitation with a final concentration of 2.5 M LiCl. After washing with 70% ethanol 2–3 times, RNAs were resuspended in 1 mM sodium citrate (pH 6.5). Truncated R2 3′ UTRs were transcribed from PCR-amplified DNA template using uridine (NEB) or pseudouridine (TriLink, catalogue no. N-1019–10) with the HiScribe T7 Kit (NEB, catalogue no. E2040S) according to the manufacturer’s instructions for RNAs < 300 nt. Purification was the same as for the mRNA with the following exceptions: following RNA synthesis 1 μL DNase RQ1 (Promega, catalogue no. M610A) was added to remove PCR template; following the RNA PCI purification step precipitation was done with a final concentration of 0.3 M sodium acetate (pH 5.2) and 3 volumes of 100% ethanol; and RNAs were resuspended in water. RNA concentrations were determined by NanoDrop and integrity verified by denaturing urea-PAGE with direct staining using SYBR Gold (Thermo Fisher, catalogue no. S11494). Sequences for mRNA and template RNA transcription are provided in Table S8.

### TPRT assays with purified protein

IR700 labelled target site oligos were ordered from IDT (Table S8). Complementary strands with only one of the two strands labelled were annealed by heating to 95 °C and cooling by 1 °C per min. TPRT reactions were assembled in 20 μL with final concentrations of 25 mM Tris–HCl pH 7.5, 75 mM KCl, 5 mM MgCl2, 10 mM DTT, 2% PEG-6 K, 5 nM target-site duplex, 0.6 μM template RNA, 50 μM dNTPs and approximately 3–10 nM R2p then incubated at 37 °C for 30 min before heat inactivation at 70 °C for 5 min. RNaseA (Thermo Fisher, catalogue no. FEREN0531) was added to a final concentration of 0.5 mg/mL and incubated at 55 °C for 30 min before dilution with 80 μL of stop solution (50 mM Tris–HCl pH 7.5, 20 mM EDTA, 0.3% SDS) spiked with a 36-nucleotide synthetic loading control (LC) oligonucleotide. Nucleic acid was purified by PCI extraction and ethanol precipitation, resuspended in 5 μL of water, and supplemented with 5 μL of formamide loading dye (95% deionised formamide, 0.025% w/v bromophenol blue, 5 mM EDTA pH 8.0). The sample was heated to 95 °C for 3 min and then placed on ice before loading a 9% urea-PAGE gel. After electrophoresis, the gel was imaged by Typhoon Trio (Cytiva). The gel was then stained with SYBR Gold and imaged again to visualise the ladder and LC.

### RT of R2 3’UTR sequences in cells

PRINT was performed largely as previously described [28]. RPE-1 hTERT cells at 50% confluency were replated at 400,000 cells per well in twelve-well plates. Cells were reverse-transfected with mRNA and template 3′ UTR RNA using Lipofectamine MessengerMAX at ½ mass/volume ratio as per the manufacturer’s instructions. 0.5 μg total RNA mixture was transfected per well of a twelve-well plate, and mRNA:template molar ratio was 1:20. Cells were collected 20–24 h after transfection and frozen as cell pellets. Genomic DNA was isolated by treatment with RNase A and Proteinase K, extraction with PCI, and precipitation as previously described [6]. For PCR, 100 ng gDNA was used in a 25 μL reaction with Q5 DNA polymerase (NEB). PCR primer sequences are listed in Table S8. PCR was as follows: 98 °C, 3 min, (98 °C 10 s, 65 °C, 30 s, 72 °C, 30 s/1 kb) 5 times with annealing temperature decreasing by 1 °C per cycle, (98 °C 10 s, 60 °C, 30 s, 72 °C, 30 s/1 kb) 25 times; 72 °C, 2 min. PCR products were analysed on 1% agarose gels containing GelRed (Fisher Scientific, catalogue no. NC9594719) and imaged using the Bio-Rad gel doc XR +—imaging system.

## Supplementary Information

Additional file 1. Structural arrangements of R2 N-termini to classify folds of novel protein conformations.

Additional file 2. B. forskalii amino acid sequence and alignment to other Ctenophora R2s.

Additional file 3. Annotated nucleotide sequence of B. forskalii R2.

Additional file 4. R2 phylogeny rooted with an ORF2 protein from human non-LTR long interspersed element 1.

Additional file 5. Multiple sequence alignment of R2 ORFs with two zinc finger N-terminal architecture.

Additional file 6. Phylogenetic tree of site-specific R2s with past B and C clade R2s.

Additional file 7. Multiple sequence alignment of Ctenophora B N-terminal architecture sequences.

Additional file 8. Phylogenetic trees of R2s based on different protein domains.

Additional file 9. Cofolding ZnFs of R2 A-lineage and some Utopia.

Additional file 10. Position-specific scoring matrix plots extended.

Additional file 11. Site-specific conservation of individual residues in lineage D and A R2s.

Additional file 12. Multiple sequence alignment of R2 proteins selected for activity testing.

Additional file 13. Immunoblot related to Fig. 4b-c.

Additional file 14. Scripts used in the study.

Additional file 15. RAxML tree of R2s.

Additional file 16: Table S1. Long and short reads used for R2 discovery pipeline. Table S2. R2 sequences from genomes covering key major classes of animals. Table S3. Species with no R2s, partial matches, and contaminant sequences. Table S4. Nucleotide and amino acid sequences of Porifera non-specifc R2 homologues and Utopia non-LTR TEs. Table S5. Porifera non-site-specific R2 flanks top BLAST hits. Table S6. R2 sequences with no co-folded ZnF3:2. Table S7. Species with two co-inherited R2 lineages. Table S8. DNA and RNA sequences used in this study. Table S9. Percentage identity matrix of R2s.

## Data Availability

All data is available for download on Zenodo [62]. The accessions of the analysed data are available in the supplementary material when applicable.
