# Supplementary material for: Lineage-specific evolution, structural diversity, and activity of R2 retrotransposons in animals
Source: Genome Biol. 2026 Apr 14;27:174. doi: 10.1186/s13059-026-04073-3 (PMC13188248; doi:10.1186/s13059-026-04073-3)
Supplement: Supplementary file 1 — Additional file 1. Structural arrangements of R2 N-termini to classify folds of novel protein conformations. [file 13059_2026_4073_MOESM1_ESM.pdf]

Additional file 1

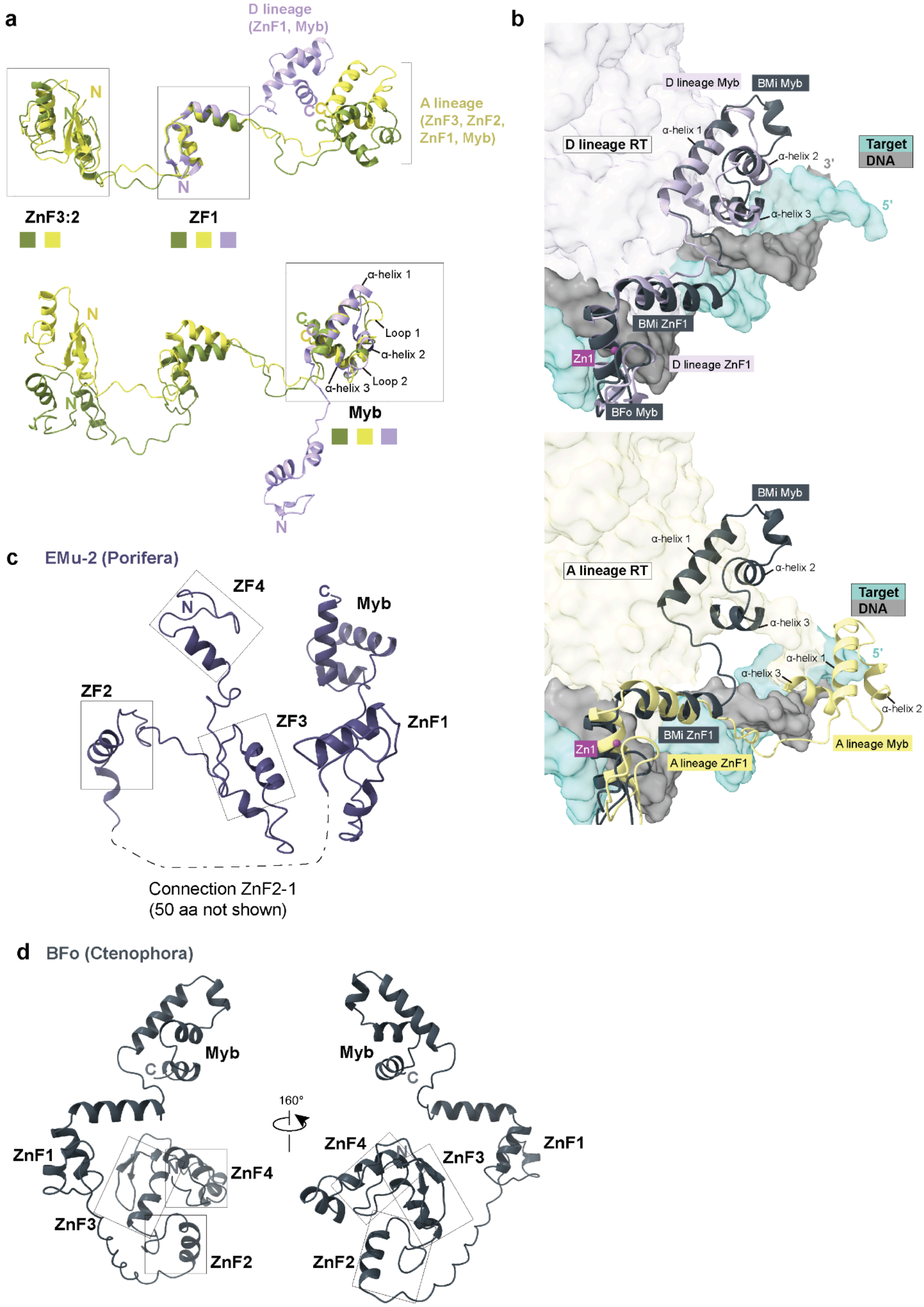

Figure S1: Structural arrangements of R2 N-termini to classify folds of novel protein conformations. (a) R2 structural references of the N-termini studied from R2 cryo-EM structures of complexes bound to the same DNA substrate, including A lineage (R2Tg - TaGu, R2Pm - PlaMe) by our group [23] and D lineage (R2Bm - BoMo) by others [28]. Three R2 N-termini are superimposed on each other and each box highlights the

structural regions that best align. Coloured squares below each domain represent the matching colours for each R2 reference (TaGu is green, PlaMe is yellow, and BoMo is purple). Top panel: ZnFs across elements can be aligned, with the exception of BoMo which only has ZnF1. Bottom panel: Myb domain requires a local alignment to confirm similar fold architecture containing three expected alpha helices connected by two loops. (b) Conformation of Ctenophore ZnF1-Myb bound to DNA relative to R2 lineages using BoMo (D lineage, top panel) and PlaMe (A lineage, bottom panel) as structural references. All ctenophores examined (n=4) with AlphaFold (AF) predictions, BMi as the representative shown, have a D-lineage-like Myb conformation, where Myb is spatially closer to the RT domain. (c) A structural representative (AF structure) of independently folded ZnFs where ZnF2-ZnF4 (boxed) do not co-fold. (d) Two orientations of an AF structure for BFo showing a distinct fold arrangement where ZnF2-ZnF4 (boxed) have shorter connections between them than model R2s in (a).
