# Supplementary material for: Lineage-specific evolution, structural diversity, and activity of R2 retrotransposons in animals
Source: Genome Biol. 2026 Apr 14;27:174. doi: 10.1186/s13059-026-04073-3 (PMC13188248; doi:10.1186/s13059-026-04073-3)
Supplement: Supplementary file 2 — Additional file 2. B. forskalii amino acid sequence and alignment to other Ctenophora R2s. [file 13059_2026_4073_MOESM2_ESM.pdf]

Additional file 2

```
>BFo_R2_shifted_from_R2_targetsite
MEEKNNETAGRTASSDKTSKSSASGSAARAAVTPAPSRACLFCGKAFKLRGLAGHTRRCESNPEWLNEKIHSSRWDFSGRSPVNCARCDLHMKATCYIGHFPDCIATNGCFRCGNQV
PVADYVEHFQVCVRTIDPAEPPTPQVVIPEVQKVTCPYCPAALQAKFSFKPGRGLSMHVKKKHPKEWNDYQLNRMELYPSQYIWREGDDELICQAIEEYQSSAPAVKKAFGINQFIQ
FRSFPHCTLDSITCHRKTSSFKEYAKDRAKRIADQQAVAESRAEESLPNEDGGHFEEVEILSEKEKSAIAAKPFGSEILEIHQHLVNRRWDTACSAAKLVFEELTCKFGKPNIPVHK
VKQGADPRPPGKKPTRKSKKRANRRRLKPSKEKRRESYAKVQKRWSKKRVDVIKSILDGKLEQVQGEKPTMEKQHDYWKSLFERPSPEDPGKVPGESSVQYELSAPLTKEEVANGLG
TAKEASTGPDGVPLSALKELGSLSLWVLYCALWMKSETPKGWRVSRTVLIDKTADECGKNPEDFRPISISSYFYRIYARSISSRLTRAVPISKRQRGFIRQDGVRDNIELFDNLVKD
AKRTLTPLSVAFMDIRKAFDSVGHASIQRALEWAGVPGRVRRVIEELYTDCYTEVGGGRIRVNRGVKQGDPLSSFLFNIVLEMALSRVPPGLGINYLGHQLFYMAFADDMILLARNA
HVLQRIVDMVSSELGLAGLEFNHAKCKVLSLVTDPRNKASMIDTDVEIAVNGDRIPPLGVLDTYRYLGIDVGVKGVPKFEPQKELKDLLQRLTKAPLKPHQRLYALRVHTMPRFQHK
LIFSKVTRNALSQMDSLVRKHVREWLKLPDDVTKAALYADVGSGGLGLICLERRVPLLKYSRIERLRDSDDELVRLLIDQEQVNKRVMQWKDRCKMSGKTYRSKEELRDLYRTQLNS
TVDGKGIRGDTRLGRLSMRSLVGPCIPLTPTRYIHAINVRLGTLQTSERKARGRVCNENGVLCDKERACHARKAVATLGHISQVCPVVHGLRVRRHDRVRDRVANQLQSRKKSVEKV
LVEKTIKLQDGTVLRPDIIVLTDTSIEVIDVQIKADMGIRRDLETDQTAKRAKYDRGDVQTSIERELSVVGMGRLYTVTALTITFRGQLPRHTVDLATRLKFKTLLPELVADVLADT
GSMFVVWHRTTGNAGKP
```

```
BFo        --------MEEKNNETAGRTASSDKTSKSSASGSAARAAVTPAPSRACLFCGKAFKLRGL
BMi        ------------------------------------------------------------
MLe        KLHRCCPLFQEKDSGGLNRSAS------------------------------------GV
PBa        TL----PFLKLNMNRLNNEKNS------------------------------------GA


BFo        AGHTRRCESNPEWLNEKIHSSRWDFSGRSPVNCARCDLHMKATCYIG-HFPDCIATNGCF
BMi        ------------------KNTMFNITPR---PEDSQVDRVTIDAESGLPNHAGANLLQCE
MLe        MSNTSHSKLNLK-MDNKLKTSLETPSGV---RADSIITRVRTSSNRG-EHSNGVTYPRCE
PBa        VRSTEFSV-----ADNRPSQSLRTTESH-----------------------------RCP
                                :                                     *

BFo        RCGNQ-VPVADYVEHFQVC----------VRTIDPAEPPTPQVVIPEVQKV-TCPYCPAA
BMi        WCDRLCKNKAGLTLHKRACKNNPAVGSSAGNTNDNRRINTP----PTMRSLFNCEYC---
MLe        ------QGVAPLDTHGGICDAPPQVTVPATETDKQKK----------------CEYC---
PBa        NCRKLCRSGNGLALHMKHC-------AKCYQNGDNRQEPVK----PRM----ECSIC---
                         *   *           .. .  .                *  *

BFo        LQAKFSFKPGRGLSMHVKKKHPKEWNDYQLNRMELY-PSQYIWREGDDELICQAIEEYQS
BMi        ---NTGYGTDRGLSAHISKKHIPEWNMIKMERFKADGPRQHVWREGDWEILCQGEEEHDR
MLe        ---EFTYLKPRQIGTHMRKRHPQEWNDIKRTKFLSE-KRQKRWLDEDFELLCIGQEEYLV
PBa        ---GLFFSGQRGVAIHKRKKHPAEWNETKRVEDTLS-RKKIRWTSGDRELLHLGMIEWEA
                 :   * :. *  *:*  ***  :  .       :  * . * *::  .  *
```

Figure S2: (Top) Translated R2 ORF from *B. forskalii*. (Bottom) Multiple sequence alignment of all Ctenophora (*Bolinopsis microptera, Pleurobrachia bachei, Mnemiopsis leidyi* (recurated from Kojima et al. 2016) and *Beroe forskalii.)* R2 N-terminal ZnFs. CXXC residues in the ZnFs are highlighted in green.
