# Supplementary material for: Lineage-specific evolution, structural diversity, and activity of R2 retrotransposons in animals
Source: Genome Biol. 2026 Apr 14;27:174. doi: 10.1186/s13059-026-04073-3 (PMC13188248; doi:10.1186/s13059-026-04073-3)
Supplement: Supplementary file 3 — Additional file 3. Annotated nucleotide sequence of B. forskalii R2. [file 13059_2026_4073_MOESM3_ESM.pdf]

Additional file 3

>WFMF01000099.1:20329-29541 Beroe forskalii isolate Bf201606 sca99

AATAGGACTTTGGTCTTATTTTGTTGGTTTCCGAGACCGAAGTAATGATTAATAGGGACAGTTGGGGGCATTCGTATTTCATTGTCAGAGGTGAAATTCTTGGATTTATGAAAGACGAACTTCTGCGAAAGCATTT
GCCAAGGATGTTTTCATTAATCAAGAACGAAAGTTGGAGGCTCGAAGACGATCAGATACCGTCCTAGTTCCAACCATAAACGATGCCGTCTGCGGATCGGAGGTCGCTAACGTAAGGCTCCTTCGGCACGCTATGA
GAAATCAAAGACTTCGGGTTCCGGGGGGAGTATGTTCGCAAGAATGAAACTTAAAGGAATTGACGGAAGGGCACCACCAGGAGTGGAACCTGCGGTTTAATTTGACTCAACACGGGAAAACTCACCAGGTCCAGAC
ATAGGAAGGATTGACAGATTGAGAGCTCTTTCTTGATTCTATGGGTGGTGGTGCATGGCCGTTCTTAGTTGGTGGAGTGATTTGTCTGGTTAATTCCGTTAACGAACGAGACCTTAACCTGCTAAATAGTGACACG
GTTATTTTAACCGTGGTTCACTTCTTAGAGGGACTATCGCTTTCAAAGCGATGGAAGTTTGAGGCAATAACAGGTCTGTGATGCCCTTAGATGTTCTGGGCCACACGCGCGTTACACTGATGAAGCCAGCGAGTAT
ATCGCCTTCACCGAAAGGTGCGGGTAATCTTGTGAAACTTTATCGTGCTGGGGATAGACCCTTGCAATTATTGGTCTTACTAATCGAACCATCTAGTAGCTGGTTCCCTCCGAAGTTTCCCTCAGGATAGCAGGAG
CTCATTCCCAGTTTTATCAGGTAAAGCGAATGATTAGAGGCCTTAGGGATGAAACATCCTTAACCTATTCTCAAACTTTAAATGGGTAAGATGTCCGACTTGCTTAATTGAAGCCGGGCCACGAATGGGAGCTCCT
AGTGGGCCATTTTTGGTAAGCAGAACTGGCGATGCGGGATGAACCGAACGTTTGGTTAAGGCGCCTAAATCGACGCTCATCAGATCCCACAAAAGGTGTTGGTTGATATAGACAGCAGGACGGTGGCCATGGAAGT
CGGAATCCGCTAAGGAGTGTGTAACAACTCACCTGCCGAATCAACCAGCCCTGAAAATGGATGGCGCTCAAGCGTCGTGCCTATACCAGACCGTTTGAGCAAATGCTAAGCTCGGACGAGTAGGAGGGCGTGGGGG
TCGTGAAGAAGCCTCTGGCGTGAGCCTGGGTCAAACGGCCTCTAGTGCAGATCTTGGTGGTAGTAGCAAATATTCAAATGAGAACTTTGAAGGCCGACGTGGAGAAGGGTTCCATGTGAACAGCAGTTGGACGTGG
GTTAGTCGATCCTAAGAGATAGGGAAACTCCGTGTTAAAGTGCCCGATCTACGACTCACGTCAATCCGGGCCGCCTATCGAAAGGGAATCGGGTTAATATTCCCGAACCGGAATGGGGGGATTCTGTCTTTCGGGA
CAGAAATGCGGTAACGCAAACGAACTGGGAGACGTCAGCCGGGAGCGGAAGCAGAGTTCTCTTTTCTGTTTAACAGACAGTCACCATGGAATGGGATTATCCCGAGATATGGTTCAGTGTCTGGTAAAGCACCGCG
CTTCTTGTGGTGTTTCCTGCCTTCCGGATGGCCCTTGAAAATCCCAGGGAGAGAATAAACTTCTCATCCGGTCGTACCCATAACCGCAGCAGGTCTCCAAGGTTAACAGCCTCTGGTCGATAGAGCAATGTAGGTA
AGGGAAGTCGGCAAAATAGATCCGTAACTTCGGGAAAAGGATTGGCTCTAAGGGTTGGGTCTGTCGGGCTGCAGCCCGAGACCTCGAAGCCTTGGGTGGACTGTCTAGAGTAAGGTTCGCCGAGCTCCGGATGGGC
CGTCCTCGGCATCGAGGAGGAAGGCTGCAGCTTCGTCTCCGGACATTTCGGCAGACGACTAACAACCAACTTAGAACTGGTACGGACAAGGGGAATCCGACTGTTTAATTAAAACAAAGCATTGCGATGGCCGGAA
ACGGTGTTGACGCAATGTGATTTCTGCCCAGTGCTCTGAATGTCAAAGTGAAGAAATTCAACCAAGCGCGGGT*CCTTAAAATTGGAATCGAAGAAGTGGGACATATGAGAAGGGAGAAT

Shift in insertion site

GAACGCTGACTTAGGCAAGAAAGTTGAGATGGAACGAAAGTAAAGAGTGTGACCCTCTTCTTCCCTCCAGCGAGTCACTGATACTTGTTCTTTAAGCCCTTGACGAAGTAAGATGAATACCTTTATACGTTATTGT
CATTTAGATGCGAGACAAGGCTGCGAGCAAGACAAGGCAAGCAATAGACCGTGGGTTCGTACCCAGTGACACCAGGGAAAAGAAAAAAAAAAAATGTCAATCTCGTGTAAGGTTATTTATTTTGGTACGGGCGGAG
AGATAGACGGCCAGGTCTACCCGTACAGGTCCGGCCAACGACTTGTCGTTCAACGGATTGCTTAGTCTAAGGAAGTCCCAATCCTGTCCTACAAGCGGAACCGCTGTTAGAGTGCGACATGGGGCACATGTGCAAC
CTCATGACAATCTCCGTCCTGAGATGGCTTCTCGGGTATGCGAAAACAGAGTGGAAGGGGCTAGTGAGCCTTGCTGGGCTCATCTCTTATTTTTAACAAGATGGAAGAAAAAAATAATGAGACTGCCGGCCGAACG
GCTAGCAGTGATAAAACCAGCAAGAGCTCGGCTTCAGGTTCAGCTGCACGCGCCGCTGTGACTCCTGCACCCAGTAGAGCTTGCCTCTTCTGTGGTAAGGCCTTTAAACTCAGAGGCCTGGCGGGACACACGCGTA
GATGCGAGTCGAATCCTGAATGGTTGAACGAGAAAATTCATAGCTCCAGGTGGGACTTCAGTGGTAGATCGCCCGTAAACTGCGCTCGTTGTGATCTTCATATGAAAGCCACATGTTATATCGGGCACTTTCCGGA
TTGCATCGCGACTAACGGATGCTTCCGATGTGGAAATCAAGTGCCCGTGGCTGATTATGTTGAGCACTTTCAAGTTTGTGTGAGGACGATTGATCCCGCTGAACCACCAACCCCACAGGTGGTTATACCTGAGGTA
CAGAAGGTGACGTGCCCTTATTGCCCCGCTGCTCTTCAAGCTAAATTTAGCTTTAAACCTGGGCGAGGCCTTTCAATGCATGTCAAGAAGAAGCACCCTAAAGAGTGGAATGATTACCAACTGAACAGGATGGAAC
TTTACCCCTCCCAGTACATCTGGCGGGAGGGTGACGATGAACTGATTTGCCAGGCGATCGAGGAGTATCAGTCCTCAGCTCCCGCGGTTAAAAAGGCGTTTGGCATCAATCAGTTTATCCAATTCAGATCCTTCCC
ACATTGCACTCTTGACAGCATAACGTGTCACAGGAAAACTTCTTCTTTTAAAGAATATGCGAAAGACCGAGCCAAACGCATAGCTGATCAACAGGCTGTGGCTGAGTCACGAGCGGAAGAGTCACTTCCCAACGAG
GATGGAGGCCACTTCGAAGAGGTAGAGATACTCTCTGAGAAGGAAAAATCGGCCATCGCTGCCAAACCTTTTGGCAGTGAAATTCTTGAGATCCATCAGCATCTTGTGAACAGGAGATGGGATACAGCATGTAGTG
CAGCTAAGTTAGTGTTTGAAGAGCTTACTTGTAAGTTCGGAAAGCCTAATATCCCGGTTCATAAAGTGAAACAAGGTGCGGATCCCAGGCCTCCAGGGAAAAAACCCACACGCAAGTCTAAAAAGAGGGCAAATAG
GCGACGACTCAAGCCCAGCAAGGAAAAGAGAAGGGAAAGTTATGCAAAGGTACAGAAGAGGTGGAGCAAGAAAAGGGTGGACGTCATCAAATCTATCTTGGACGGCAAATTGGAGCAAGTACAAGGAGAAAAACCA
ACGATGGAGAAGCAGCACGATTACTGGAAGAGTCTGTTTGAAAGGCCTAGTCCTGAAGATCCAGGTAAAGTGCCGGGGGAAAGTTCAGTACAATATGAATTGAGTGCTCCGCTGACGAAGGAAGAGGTAGCCAACG
GCTTAGGAACAGCCAAAGAGGCCTCGACCGGACCAGATGGGGTTCCTCTCTCTGCGTTAAAGGAACTTGGTTCCTTGAGTCTGTGGGTCCTGTACTGTGCGCTGTGGATGAAGTCGGAAACTCCGAAGGGTTGGAG
GGTTAGCCGGACGGTCTTAATAGACAAGACGGCGGACGAATGTGGGAAAAACCCGGAGGACTTCCGACCGATATCCATCAGTTCGTACTTTTACAGGATCTACGCGAGATCGATCAGCTCGCGCTTGACGCGAGCA
GTTCCAATCTCGAAAAGACAGCGTGGATTCATAAGGCAAGATGGTGTCAGAGACAATATCGAATTGTTCGATAATTTGGTCAAGGACGCCAAGCGAACCTTAACCCCCTTGAGCGTGGCCTTTATGGATATAAGGA
AGGCGTTTGACAGTGTTGGCCACGCGAGTATTCAGCGTGCCCTGGAATGGGCTGGTGTCCCTGGTCGGGTGAGGAGGGTCATCGAGGAACTATACACGGACTGCTATACGGAGGTTGGAGGGGGCAGGATTCGTGT
GAACAGAGGGGTGAAGCAGGGTGACCCGCTGAGCTCCTTCTTGTTCAATATAGTTCTCGAGATGGCACTCAGTAGAGTTCCACCAGGCCTGGGCATCAACTATCTGGGGCATCAACTGTTTTACATGGCCTTCGCT
GATGATATGATTCTTCTAGCTAGGAATGCCCACGTGCTACAAAGAATAGTGGACATGGTGTCAAGCGAATTAGGGTTAGCTGGTTTGGAATTTAACCACGCTAAGTGTAAAGTTCTCTCCCTGGTTACGGACCCCA
GGAATAAAGCGAGTATGATAGACACTGACGTCGAGATAGCGGTAAACGGTGATCGTATCCCACCCCTCGGGGTGTTGGATACCTATCGTTATCTAGGGATAGACGTCGGAGTGAAGGGAGTTCCCAAATTCGAACC
GCAGAAGGAACTCAAAGACTTGCTTCAGAGACTCACGAAAGCACCGTTGAAACCACATCAGCGGCTCTATGCCCTGAGAGTTCACACCATGCCTCGATTCCAGCACAAGCTTATCTTTTCGAAGGTAACTAGGAAC
GCTCTAAGCCAAATGGACTCATTAGTCAGGAAGCACGTAAGAGAGTGGCTCAAATTACCCGACGATGTCACCAAGGCGGCGCTGTACGCCGACGTTGGGTCTGGAGGATTGGGCCTCATCTGCCTCGAACGAAGGG
TACCCCTCCTGAAATATTCGAGGATAGAAAGATTACGCGATTCTGATGACGAACTAGTCCGATTGTTAATCGACCAAGAACAGGTGAACAAGCGAGTGATGCAATGGAAAGATCGTTGTAAGATGAGTGGCAAGAC
CTATCGTAGTAAAGAGGAGCTCAGGGATCTGTATAGAACCCAATTGAACTCCACAGTCGATGGGAAGGGTATTAGGGGAGATACCCGGCTAGGCAGACTATCGATGCGGTCGCTCGTTGGACCCTGCATCCCACTC
ACTCCAACTAGATACATCCATGCTATAAACGTTCGTCTTGGAACCCTACAGACTTCCGAGAGAAAGGCCAGAGGAAGGGTCTGCAACGAGAACGGCGTCCTATGTGACAAAGAGAGGGCATGTCACGCTAGGAAAG
CTGTTGCAACTCTAGGTCATATATCTCAAGTATGCCCTGTAGTACACGGTCTCAGAGTGAGAAGGCACGACCGAGTAAGAGACCGTGTTGCCAACCAACTGCAGAGCAGAAAAAAGTCTGTTGAGAAAGTACTGGT
TGAGAAGACGATAAAACTCCAGGATGGTACTGTGCTGAGACCGGACATCATTGTTCTCACTGACACTTCTATTGAGGTTATTGATGTCCAAATAAAGGCTGATATGGGAATCCGGAGGGACCTCGAGACCGACCAG
ACCGCAAAGAGAGCGAAGTACGACAGGGGCGACGTACAAACATCGATAGAACGTGAATTGTCGGTGGTTGGGATGGGGAGGCTGTATACAGTTACGGCCTTGACAATCACGTTCAGAGGGCAGCTGCCAAGACACA
CAGTTGATCTGGCTACGCGTCTTAAATTCAAGACGCTATTGCCAGAACTAGTGGCTGACGTCTTGGCAGACACGGGTAGTATGTTTGTGGTCTGGCACAGGACGACGGGAAACGCAGGAAAACCGTAGATGCCTTT
GTCTTAGGGATAGAAAAAGTCCCCCCTCTGCTACATCATCAGCGAGGCCAGGGACGCCAATAGTCACAGTAGGCTGGTTGTTTTACCATCAACGTAAAAGAGAACGTGTCAGTATCTTCCCACCTACTGCATAAAT
GGAACTCGATTCGACCAGGATGAGTTTGACTTGCACTACGAACGCAGGGTCACAGCTTAGTAGTGAGTTTGAACGCGATGATGACGGTGCTATGCACTGCTGATGATCTGAAGTCGCTGCTATTGCTCGAAGCAAA
CGCAGGCCACGAACATCACAGATCGTCGTATAAATGTCGTCCAGCGCAGCTTGCCCTGAAAGCGAGCTGCGGCGTCATCTGAGGAAGCCATGGCGACGGGGATGAAGCGGATGAACCATAAACATCGTCTTCGGAC
ACAAAAAAAGTAGAAAAATGCTGATCAGTACGTTCGTGGCTGATACGGCTTCCTGCGGATCCGTCACAGCCATGGTGGAATGGATGACACTGCCCTAGAAAAATCAGGGGCTCACCGCTCAGTACGGCGAGTCTTC
CACAGAGGGTGAAGCCTCAGAAGGGAGTCTCGACTCGTCCTCTCAATCCATTGGGCGAGATAGGAGTTGGCCAAACCTGACGAGAGCAGGCCGATTCCCGAGGGTACGAGGCCTCCCCCCGGGGAGGTTCTCAAAA
ATCGACGGTGAGTGACGACGCGATGAGCGTACGGACGCTATCAGTGGCCGAAAGTAGAAACTCTTCCCGATATTTTCCGTAGGTCGCAAGGCCGGTATCGGGGGAAAGCATGAATTCTTACGATTTCACAGCGAAA
GAGT*CTCTCTTAAGG*TAGCCAAATGCCTCGTCATCTAATTAGTGACGCGCATGAATGGATTAACGAGATTCCCACTGTCCCT

B. forskalii shifted and normal R2 insertion site

ATCTACTATCTAGCGAAACCACAGCCAAGGGAACGGGCTTGGCATAATCAGCGGGGAAAGAAGACCCTGTTGAGCTTGACTCTAGTCTGACTTTGTGAAAAGACATGAAGGGTGTAGCATAAGTGGGAGCGTAAGC
GACATTGAAATACCACTACTTTCATCGTTTTTTTACTTATTCGGTGAAGCGGAAGCGAGGTGCAAGCCTCACCTTCTAGATTTAAGCCGCACCTTTCGGAGCGGTGATCCGAGCCGGAGACACAGTCAGGTGGGGA
GTTTGGCTGGGGCGGCACATCTGTCAAACGATAACGCAGGTGTCCTAAGGTGAGCTCAAAGAGAACGGAAATCTCTTGTAGAACAAAAGGGTAAAAGCTCACTTGATTTTGATTTTCAGTATGAATACAAACTGCG
AAAGCATG

Figure S3: Nucleotide sequence of *Beroe forskalii* R2 annotated to show likely start codon (red), coding sequence (green) and stop codon (red). R2 target sites are in bold and underlined. The normal and shifted insertion sites are denoted by asterisks (black and red, respectively). 5' rDNA is in blue.
