# Supplementary material for: Lineage-specific evolution, structural diversity, and activity of R2 retrotransposons in animals
Source: Genome Biol. 2026 Apr 14;27:174. doi: 10.1186/s13059-026-04073-3 (PMC13188248; doi:10.1186/s13059-026-04073-3)
Supplement: Supplementary file 4 — Additional file 4. R2 phylogeny rooted with an ORF2 protein from human non-LTR long interspersed element 1. [file 13059_2026_4073_MOESM4_ESM.pdf]

Additional file 4

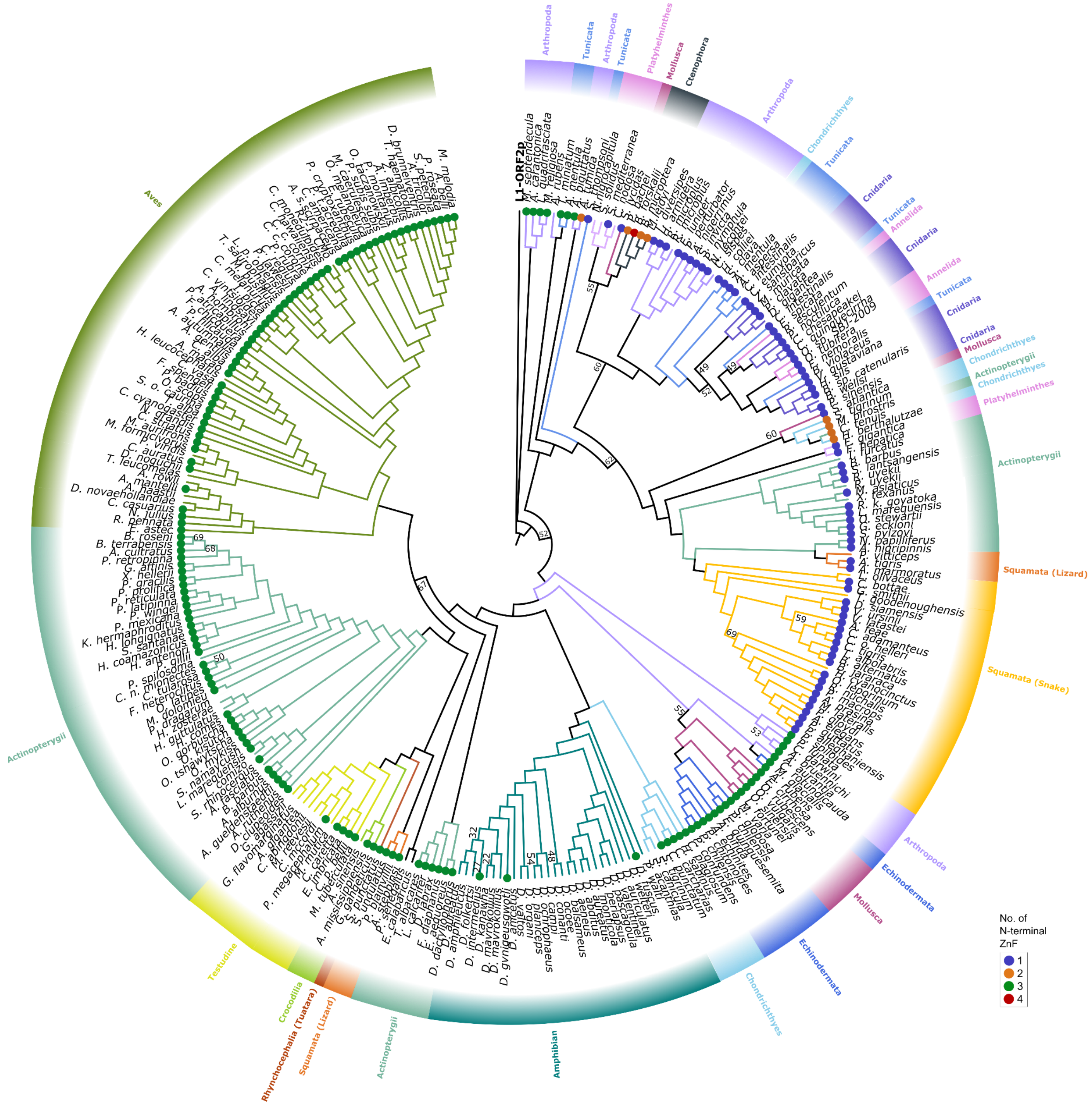

Figure S4.1: Topology only (branch length ignored) phylogenetic tree of all site-specific R2s with a protein coding region rooted with human L1 (MFP model finder, 1000 bootstraps, support values under 70 shown). Coloured circles indicate the number of N-terminal zinc fingers. R2s without a coloured circle indicate the element has a partial ORF.

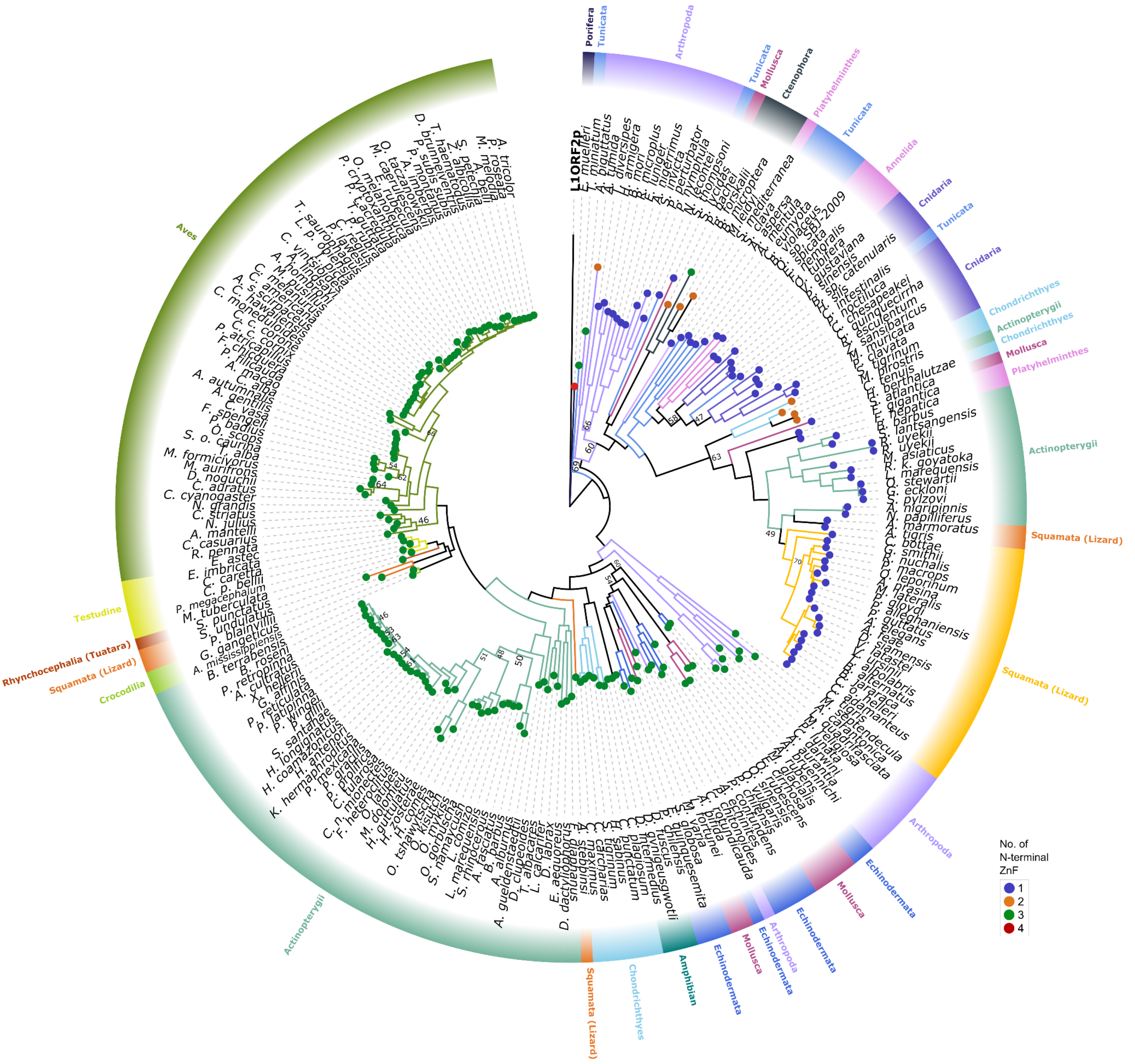

Figure S4.2: Phylogenetic tree of site-specific and site-non-specific R2-homologous RT domains rooted with human L1 (MFP model finder, 1000 bootstraps, support values below 70 are shown). Coloured circles indicate the number of N-terminal zinc fingers.

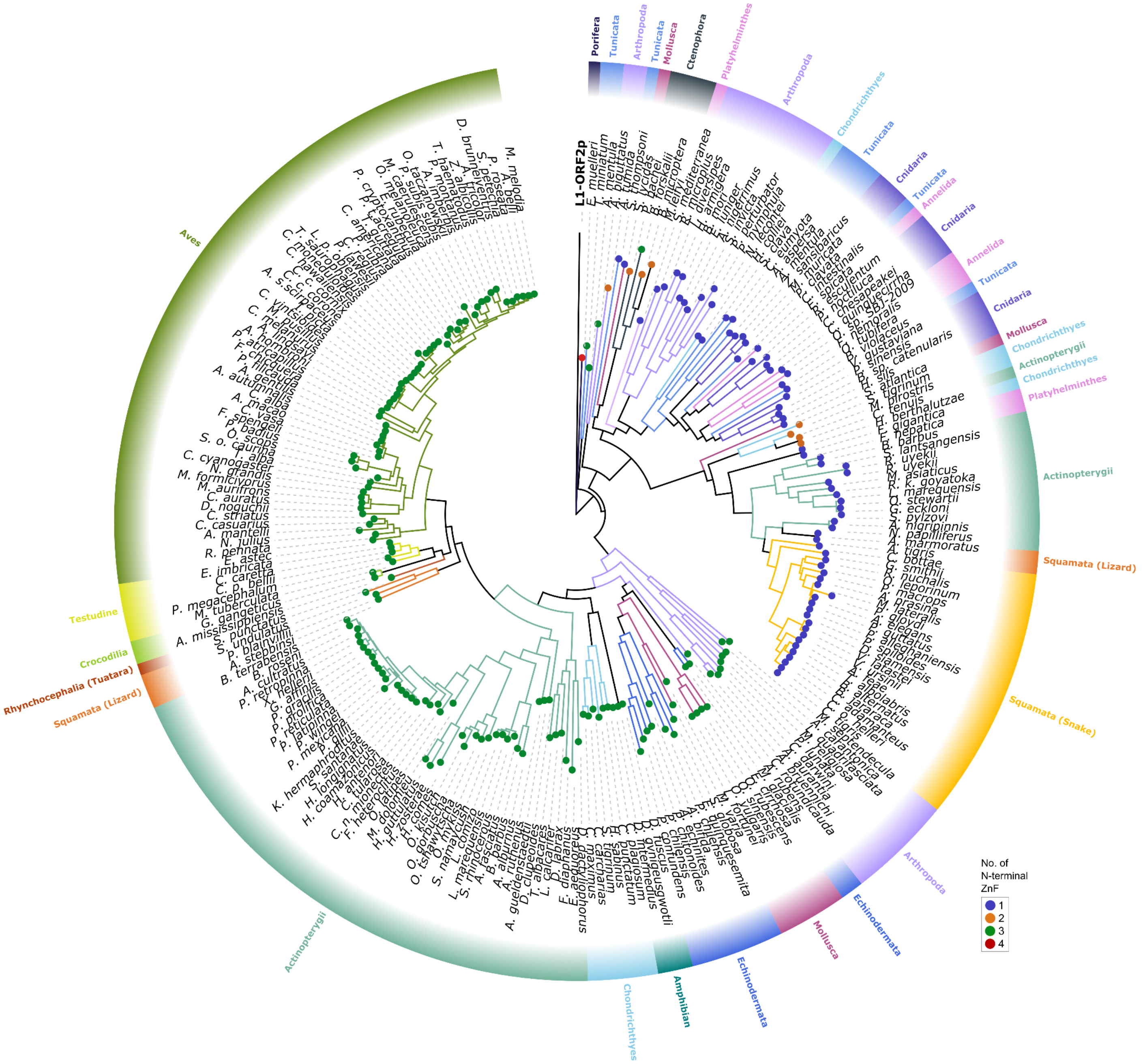

Figure S4.3: Phylogenetic tree of site-specific and site-non-specific R2-homologous trimmed ORFs (>900 amino acids) rooted with human L1 (MFP model finder, 2000 bootstraps, all nodes supported with support values over 90). Coloured circles indicate the number of N-terminal zinc fingers.
