# Supplementary material for: Lineage-specific evolution, structural diversity, and activity of R2 retrotransposons in animals
Source: Genome Biol. 2026 Apr 14;27:174. doi: 10.1186/s13059-026-04073-3 (PMC13188248; doi:10.1186/s13059-026-04073-3)
Supplement: Supplementary file 5 — Additional file 5. Multiple sequence alignment of R2 ORFs with two zinc finger N-terminal architecture. [file 13059_2026_4073_MOESM5_ESM.pdf]

Additional file 5

```
R2-1_BMi    VDRVTIDAESGLPNHAGANLLQCEWCDRLCKNKAGLTLHKRACKNNPAVGSSAGNTNDNR
R2-1_MLe    ITRVRTSSNRG-EHSNGVTYPRCE------QGVAPLDTHGGICDAPPQVTVPATETDKQ-
R2-1_PBa    --------------------HRCPNCRKLCRSGNGLALHMKHC-------AKCYQNGDNR
R2-1_STig   MPEATASAH---SSQRGTAMFVCEHCGKEYRSRSGLSGHRRMH-------FAEGETRAV-
R2-1_MBi    ------------ESQIG---YVCPDCGRAFRSRSGMSNHRRTH-------AAAGAVGGR-
R2-1_HBer   ------------KSQTG---FPCPDCGRVYRSRSGMSNHRRTH-------LAQEGGDDG-
R2-1_CTe    ------------KSQTG---FPCPDCGRVYRSRSGMSNHRRTH-------LAQEGGDDG-
R2-1_ATu    ------------DNSTLNIDRRCGLCGVTFNTKSKLKSH-----------ILSRSCSDR-
                                               :  *

R2-1_BMi    RINTPP---TMRSLFNCEYCNTGYGTDRGLSAHISKKHIPEWN---------------MI
R2-1_MLe    --------------KKCEYCEFTYLKPRQIGTHMRKRHPQEWN---------------DI
R2-1_PBa    QEPVKP-----R--MECSICGLFFSGQRGVAIHKRKKHPAEWN---------------ET
R2-1_STig   --PI----------WKCDICQAAFGTKIGLSQHQRRQHAEVDN---------------QR
R2-1_MBi    --------------FSCGICSESFLSKAGLAQHTRHRHPVEHN---------------IR
R2-1_HBer   --------------FRCDICSAVFRTKAGLGQHTRRQHPVQHN---------------IR
R2-1_CTe    --------------FRCDICSAVFRTKAGLGQHTRRQHPVQHN---------------IR
R2-1_ATu    --------------SACKFCGRSFNTFAGVRQHERRVHPLEYASDLQSVIGKASESVIME
                            *  *   :     :  *  : *
```

Figure S5: Multiple sequence alignment of R2 ORFs with two ZnF N-terminal architecture: Ctenophora: *Bolinopsis microptera*, *Pleurobrachia bachei*, *Mnemiopsis leidyi* (re-curated, first found Kojima 2016). Chondrichthyes: *Mobula birostris, Stegostoma tigrinum, Hypanus berthalutzae*. Actinopterygii: *Cynodonichthys tenuis.* R2-1_MLe has one ZnF because of an internal truncation. CXXC residues in the ZnFs are highlighted in green.
