# Supplementary material for: Lineage-specific evolution, structural diversity, and activity of R2 retrotransposons in animals
Source: Genome Biol. 2026 Apr 14;27:174. doi: 10.1186/s13059-026-04073-3 (PMC13188248; doi:10.1186/s13059-026-04073-3)
Supplement: Supplementary file 6 — Additional file 6. Phylogenetic tree of site-specific R2s with past B and C clade R2s. [file 13059_2026_4073_MOESM6_ESM.pdf]

Additional file 6

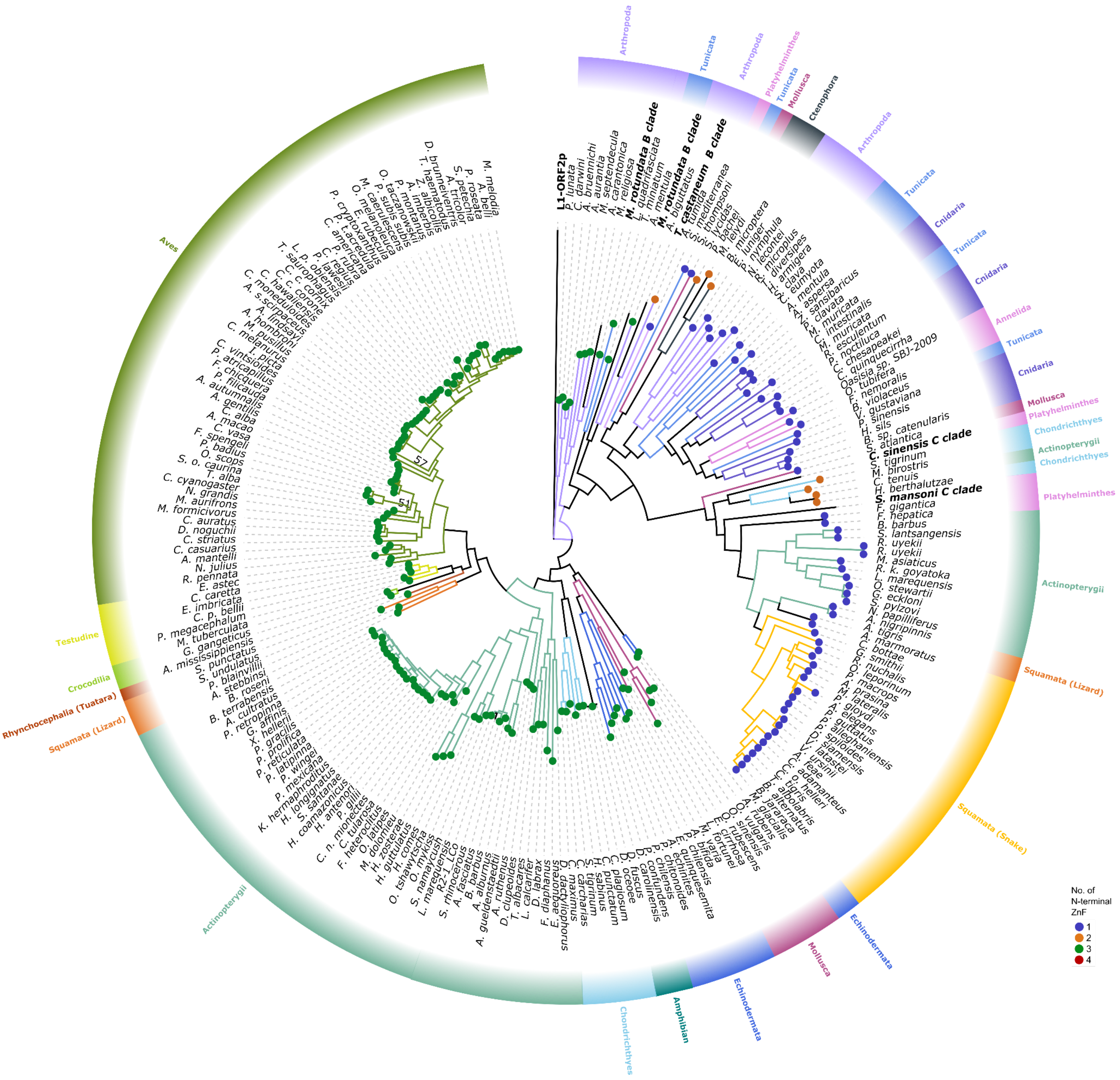

Figure S6: Phylogenetic tree of site-specific R2s constructed with IQTree (R2 ORF, MFP model finder, 1000 replicates with human L1 as an outgroup. Bootstrap values under 60 are shown at nodes. The phylogenetic tree also includes R2s with two N-terminus ZnF R2s in bold text from Kojima et al. 2016.
