# Supplementary material for: Lineage-specific evolution, structural diversity, and activity of R2 retrotransposons in animals
Source: Genome Biol. 2026 Apr 14;27:174. doi: 10.1186/s13059-026-04073-3 (PMC13188248; doi:10.1186/s13059-026-04073-3)
Supplement: Supplementary file 7 — Additional file 7. Multiple sequence alignment of Ctenophora B N-terminal architecture sequences. [file 13059_2026_4073_MOESM7_ESM.pdf]

Additional file 7

```
R2-1_BMi   ----------------------------------------KNTMFNITPRPEDSQVDRV
R2-1_MLe   KLHRCCPLFQEKDSGGLNRSASGVMSNTSHSKLNLKMDNKLKTSLETPSGVRADSIITRV
R2-1_PBa   TL----PFLKLNMNRLNNEKNSGAVRSTEFS----VADNRPSQSLRTTESH---------
                                                    . :: .

R2-1_BMi   TIDAESGLPNHAGANLLQCEWCDRLCKNKAGLTLHKRACKNNPAVGSSAGNTNDNRRINT
R2-1_MLe   RTSSNRG-EHSNGVTYPRCE------QGVAPLDTHGGICDAPPQVTVPATETDKQKK---
R2-1_PBa   -----------------RCPNCRKLCRSGNGLALHMKHC-------AKCYQNGDNRQEPV
                            :*       :.   *  *   *         . :...:::
R2-1_BMi   PPTMRSLFNCEYCNTGYGTDRGLSAHISKKHIPEWNMIKMERFKADGPRQHVWREGDWEI
R2-1_MLe   ---------CEYCEFTYLKPRQIGTHMRKRHPQEWNDIKRTKFLSE-KRQKRWLDEDFEL
R2-1_PBa   KPRME----CSICGLFFSGQRGVAIHKRKKHPAEWNETKRVEDTLS-RKKIRWTSGDREL
                    *. *   :   * :. *  *:*  ***  *  .   .  ::  * . * *:
R2-1_BMi   LCQGEEEHDRLPSTAKNKLGVNMFIRASYFPQLTIQAISCQRKSPNFHRY--KNDRNLQD
R2-1_MLe   LCIGQEEYLVLSSIGKQGKGINQYIQTKYFPTLSTDAIKSQRKSRRFSEYSEKRSRELQP
R2-1_PBa   LHLGMIEWEASAKTHSQ----EDWVRDHKFPERTINSIKCQIRSKLFRRYVAARPRDEAP
           *  *  *    ..  .:    : :::   **  : ::*..* :*  * .*   . *:
R2-1_BMi   PVTEPQPELEPIPEIEIPAQASNPTNNPLEFTPLAEDIADEIRGKPLGPEILNVYRLLAQ
R2-1_MLe   CNTSSDPE-------ELPNEAVT-ENSPLSFDPLDRDVVKKISSKDHGDQILLVQEHLIN
R2-1_PBa   ---APTPE-AAIQEDEC-------AGSILC---LKDAAIRWLEKEPDGEEYLAIYERLQD
               . **       *         .. *    *       :  :  * : * : . * :
R2-1_BMi   AQFNEANDKSRFIFDELAKDFTRLSTVPKQPTTSKNKNKKKVGKKRISQRPEKKRLSPSK
R2-1_MLe   GRYQEANTLAKAIFEKLSGKFPNLKTGDHRP--GKQQTARKVGKKRV--RGSGKKLSPSK
R2-1_PBa   GRLGEAGERSRRKFDELASKYT--------PLAKSQKPAKGRSKPVDLKSKTNGKLSPAK
           .:  **.  ::  *::*: .:.        *   .::  :  .*          :***:*
R2-1_BMi   AKRKELYAIVQKKWH-TKKRSSVIDTILRDSLGKV---REPAHTPEQLAEFWKGLFSRES
R2-1_MLe   QNRRELYAIVQKQWR-TKKRSKVINQILTGNLNKE---QSYTHTPDQLAQFWSTLFGRVS
R2-1_PBa   RRRRAKYGQCQKEWHNNNQRSGLIRSILQGKFGSKEVLKDPRETEETI-DFWSGIFNRDS
            .*:  *.  **:*: .::** :*  ** ..:..    :.  .* : : :**. :*.* *
R2-1_BMi   PPDNRPIPNPRTEIPQLDNPILVSEVTDSLKRATEKATGLDGVPLKHLREIGATALTILY
R2-1_MLe   PRDDRPINHRRSVIPELDKPLSVEEVEAALKGAKDAATGIDGVPISHLKHLGSAALTILY
R2-1_PBa   TPDSREIENARETIGALDNMITVEEVAKALKGKSERARGPDGVPHKCLKELGATKLAILY
           . *.* * : *  *  **: : *.**  :**  .: * * **** . *:.:*:: *:***
R2-1_BMi   NGLYCKQLIPTSWKEARTVLIPKCDVPSSPGEYRPITISSYYYRIYTSTIGRRLSDSVGL
R2-1_MLe   NGLYVTGSIPDPWKRARTILIPKSNPPASPGDYRPISISSYFYRIYTSAISKRLASAVSL
R2-1_PBa   NGVFCTSEVPDSWREARTVLIPKKEEPKGPADYRPITIGSYFYRAYTSVLGGRISDVVRP
           **:: .  :* .*:.***:**** : * .*.:****:*.**:** ***.:. *::. *
R2-1_BMi   SNRQKGFIKADGIRDNLILLETIIEDSKKTSSPLHMTFMDVKKAFDSVSHHSIRRALEWA
R2-1_MLe   DDRQKGFIKEDGIRDNLSLIDTLINETKAGSKSLFMTFMDVKKAFDSVSHYAIARSLEWA
R2-1_PBa   SQRQKGFVRSDGIRDNLCLLDGLIYNSKNRVQPLHMSFMDVRKAFDSVSHFSIQRMLRWS
           .:*****:: ******* *:: :* ::*   ..*.*:****:********.:* * *.*:
```

```
R2-1_BMi   GVPLGLRNVIADLYKDCSTRIGLSDIKVTRGVKQGDPLSSILFNLVVEMALSKIPDRLGI
R2-1_MLe   GVPDGMRSVIADLYQDCTTDICGRSVKVTRGVKQGDPLSSTLFNLVIEMVMSNVPERLGI
R2-1_PBa   GIPPLLRSVINDLYIGATTSVLGKTVPVKVGVKQGDPLSSILFNLVLDMALDGLTDELGV
           *:*  :*.** *** ..:* :    : *. ********** *****::*.:. :.:.**:

R2-1_BMi   GYLGHRLFYMAFADDLVILSRSQLTNQTLVDRVTEQLGLVGLELHPNKCKSVAVRADAKR
R2-1_MLe   QFQGHRLFYLAFADDLVLLTRGPTANQKLVSLVHEQLARVGLELHPGKCKSIAIMADPKR
R2-1_PBa   SYLGERLCWMAFADDLVILAPSRATLQELISNITDRLKRVGLVMNGDKCKSLSICADGKR
            : *.** ::*******:*:  . : * *:. : ::*  *** :: .****::: ** **
R2-1_BMi   KTTFVDSTQTIRVNGSEIPALNSEGWYKYLGIKVSSSGMPQGGYTDEIKLLLERVTRAPL
R2-1_MLe   KTTFVDQGSSVLIGGEPVSSLGPQEWYKYLGIKLGSGGMPQGIYRDQLADLLAKTDSAPL
R2-1_PBa   KRTYVDTSQKLYIEGNMLDSMSITDYYTYLGIGNGARGVKKENLHSEWKTLLERTDKAPL
           * *:**  ..: : *. : ::.   :*.****  .: *: :    .:   ** :.  ***
R2-1_BMi   KPHQRMFILRTHILPRFNHRMMFEKVACKTLTEIDILVREYVRRWLKLPKDVPVAAFYTD
R2-1_MLe   KPQQRLYILRSHILPKFNHRLMFERVTCQTLEGLDKLIRTHVRKWLKLPKDTPGPAFYAD
R2-1_PBa   KPHQKLYVVKKHAYPTLQHKCSFQYASKKCLTELDKLTRRYVRKWMWLPQDTTIEAFYAS
           **:*::::::.*  * ::*:  *: .: : *  :* * * :**:*: **:*..  ***:.
R2-1_BMi   VPSGGLGLLSLRTRIPLLKRQRTERMAESSDPIIRLLVHQEPSKTRLTIGKKRCRIFGKN
R2-1_MLe   KGSGGLGLITLRYRVPLLKLRRHKKMADSPDPVIRLIPNAEPTISLLARWTKMCSLYGKQ
R2-1_PBa   VEDGGLQLPSFRYEVPLNKFRRLCKMRTSEDPLVRKLANAEPAKTKIDNAKKLCVVDGNS
             .*** * ::* .:** * :*  :*  * **::* : : **: : :   .* * : *:.
R2-1_BMi   YHSKSQLASITREKFWSTCDGKGLRTVVPINTSKSSFKLLSDDRTSLKAAQYLGAISVRL
R2-1_MLe   YQHKSELSKIIRDKYWTMCDGKGLRTEVPPDTAKKTLSLLFEDRTPLKPGQLIGAIGVRL
R2-1_PBa   IENKSQLKSRVRDAYWKSYDGRGLRTE-PQQVKRGNFGQLQGAVTTLSTRNLVGAWNIRL
            . **:* .  *: :*.  **:****  * :. : .:  *    *.*.. : :** .:**
R2-1_BMi   NCLGTPLRNNRGGMKPAI---HNLCDKCPGQKFASLGHISQTCPATHGLRVKRHDKVVTR
R2-1_MLe   NTLGTPARNNRAKGYSPE---ANICDKCPGNRQATLGHISQTCPATHGRRVKRHDKIVNR
R2-1_PBa   NTTQTPARKNRAGGAGGAGDSASTCDKCPNGRLATLGHISQSCPETHGSRTKRHDRVVDH
           *   ** *:**.          . *****. : *:******:** *** *.****::* :
R2-1_BMi   LAKHFKGKENTLTVLVEPQLKYGNLPMQKPDLVINTGSTVEIIDIQIKADQGIPRDEDID
R2-1_MLe   IAKALKERGSVKNILTEPHLRHDKLPLRKPDLIVHTEKSVEIIDVQVVADQGISRHEDED
R2-1_PBa   LQSALLNAQGVTSVLKEPEIRPKGKSYCKPDLVVAMAERVVVVDVQITSDGGIEDLEGV-
           : . :    .. .:* **.::    .  ****::   . * ::*:*: :* **   *.
R2-1_BMi   ETVKKDKYDTQECRRAAYLALGVTPGSLPCNVNAFTLTWRGNPAPHSYKLARRLGFTSIM
R2-1_MLe   QQKKIVKYDVDGYKRAAYKMLGIDYGSIPCNVSAFTITWRGNLAPHSLKLASRLQFSPVL
R2-1_PBa   AKRKTDKYGSPEILKATLLHLGL-PSDTPISVHAFSITWRGNTLRHSLETASTLGVTHIL
              *  **.     :*:   **:  .. * .* **::*****   ** : *  * .: ::
R2-1_BMi   KYLIADALVDTWGMFVVWNCTS------------
R2-1_MLe   KYIVADSLVDTWGAFLIWGKTS------------
R2-1_PBa   PRVTTDVLVDTYRMFLGWRASGRRCVPNKKSTLK
             : :* ****:  *: *  :.
```

Figure S7: Multiple sequence alignment of Ctenophora B N-terminal architecture sequences: *Bolinopsis microptera*, *Pleurobrachia bachei*, *Mnemiopsis leidyi* (re-curated, first found Kojima 2016). CXXC residues in the ZnFs are highlighted in green.
