# Supplementary material for: Lineage-specific evolution, structural diversity, and activity of R2 retrotransposons in animals
Source: Genome Biol. 2026 Apr 14;27:174. doi: 10.1186/s13059-026-04073-3 (PMC13188248; doi:10.1186/s13059-026-04073-3)
Supplement: Supplementary file 8 — Additional file 8. Phylogenetic trees of R2s based on different protein domains. [file 13059_2026_4073_MOESM8_ESM.pdf]

Additional file 8

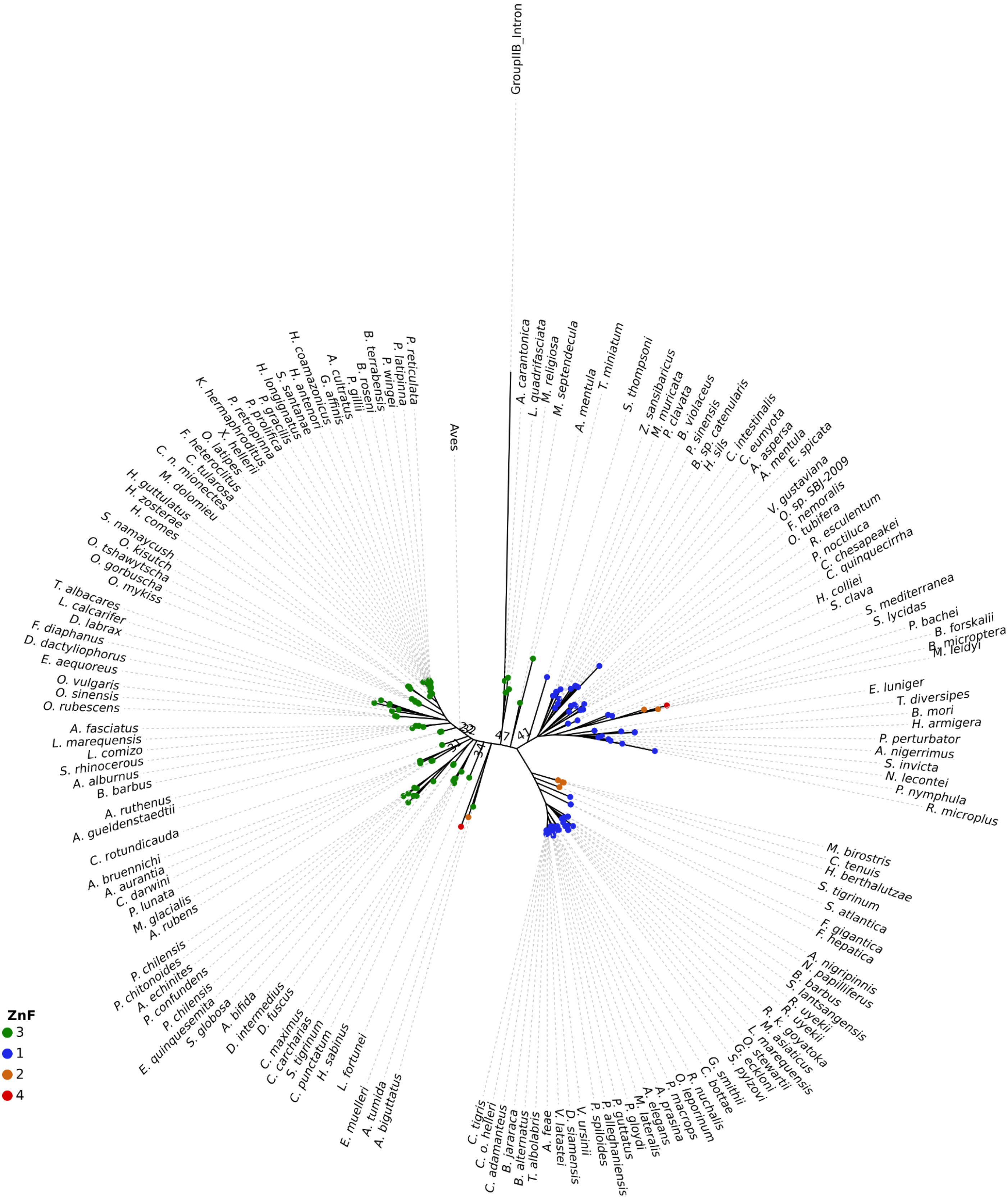

Figure S8.1: Phylogenetic tree of only R2 RLE domains constructed with IQTree (MFP model finder, 1000 replicates). The number of N-terminal ZnFs is annotated at each node. Bootstraps under 50 are shown. Aves branch is collapsed.

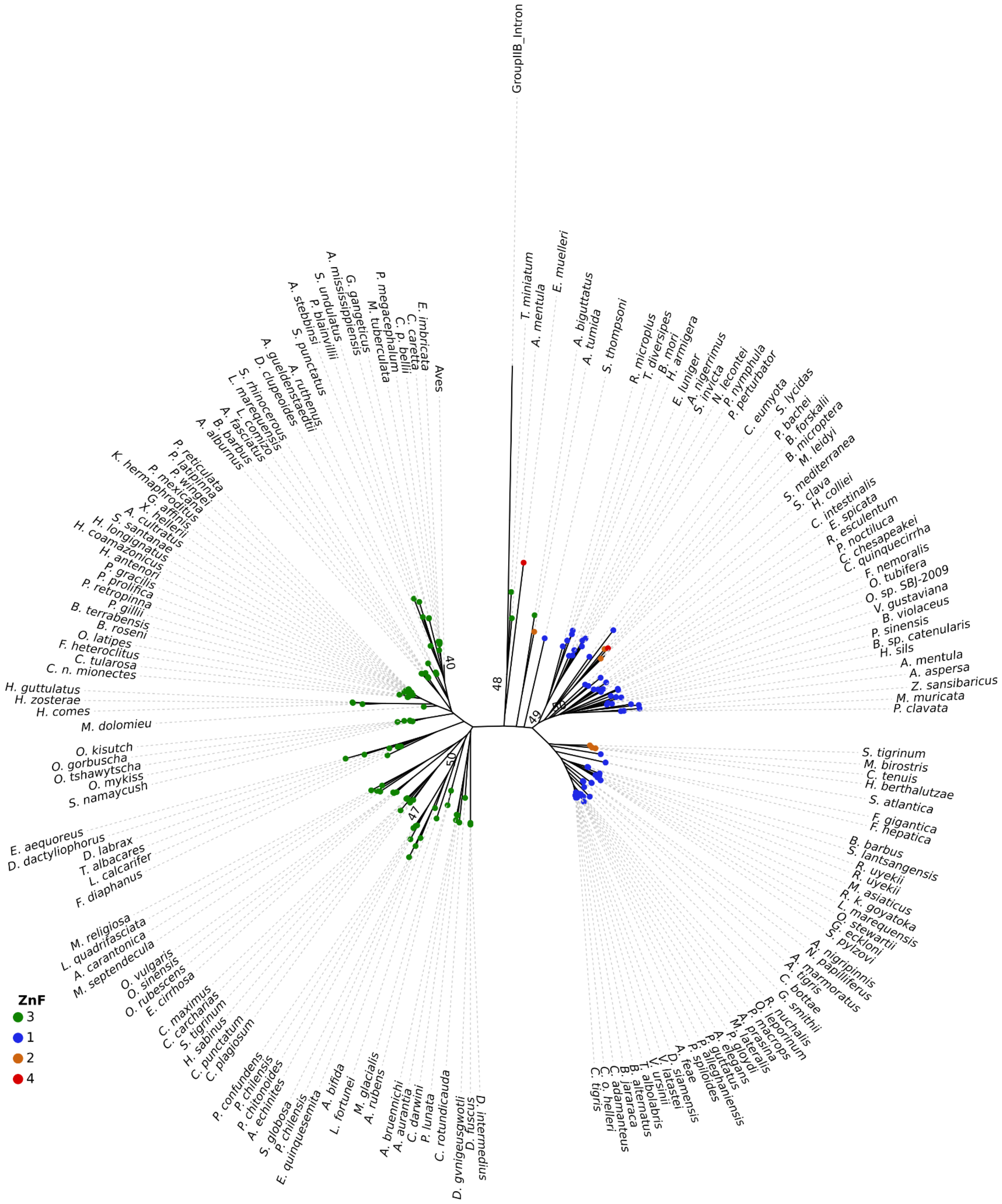

Figure S8.2: Phylogenetic tree of only R2 Thumb and ZnK domains constructed with IQTree (MFP model finder, 1000 replicates). The number of N-terminal ZnFs is annotated at each node. Bootstraps under 50 are shown. Aves branch is collapsed.

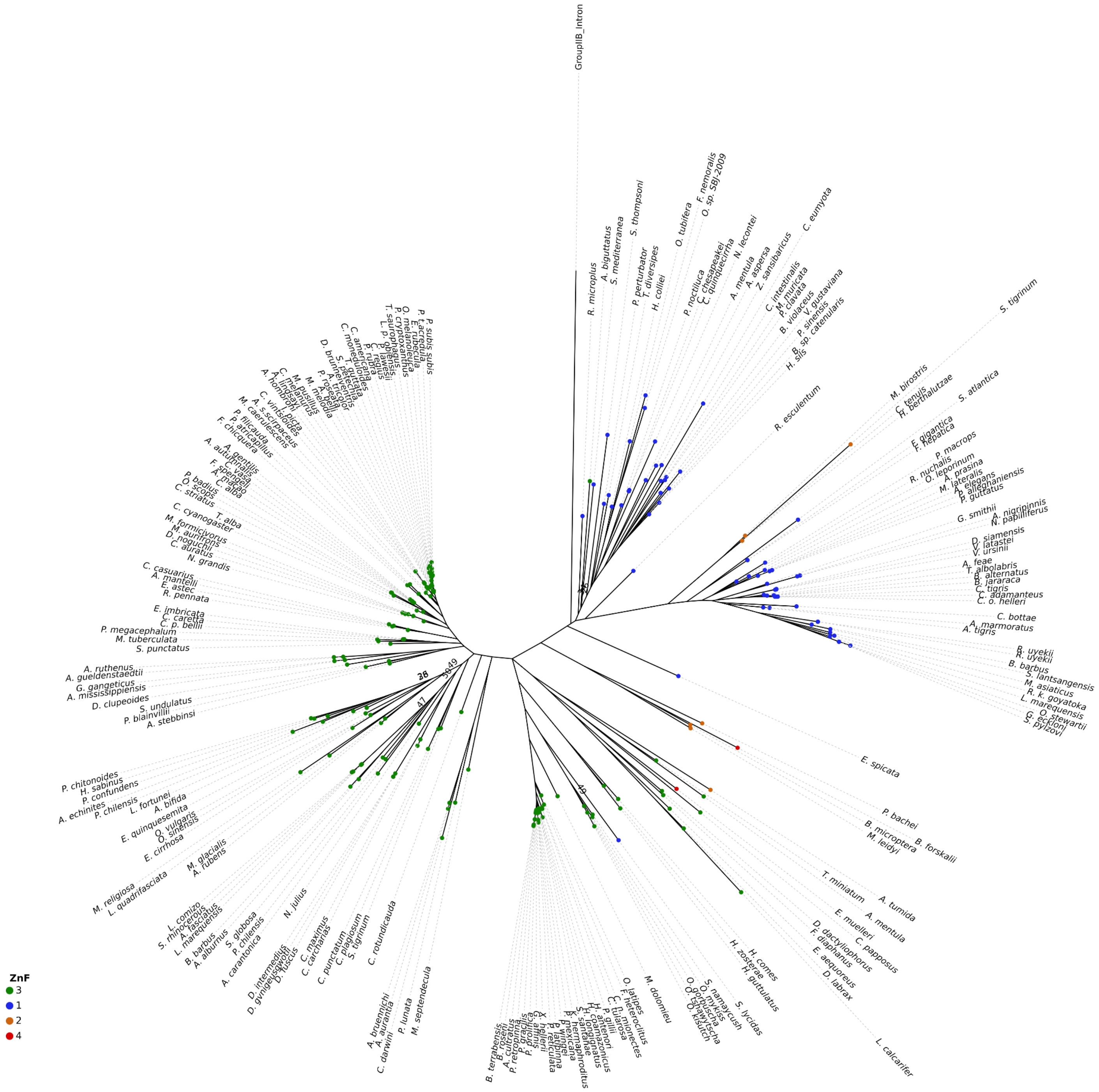

Figure S8.3: Phylogenetic tree of only R2 ZnF-Myb domains constructed with IQTree (MFP model finder, 1000 replicates). The number of N-terminal ZnFs is annotated at each node. Bootstraps under 50 are shown.
