# Supplementary material for: Lineage-specific evolution, structural diversity, and activity of R2 retrotransposons in animals
Source: Genome Biol. 2026 Apr 14;27:174. doi: 10.1186/s13059-026-04073-3 (PMC13188248; doi:10.1186/s13059-026-04073-3)
Supplement: Supplementary file 9 — Additional file 9. Cofolding ZnFs of R2 A-lineage and some Utopia. [file 13059_2026_4073_MOESM9_ESM.pdf]

Additional file 9

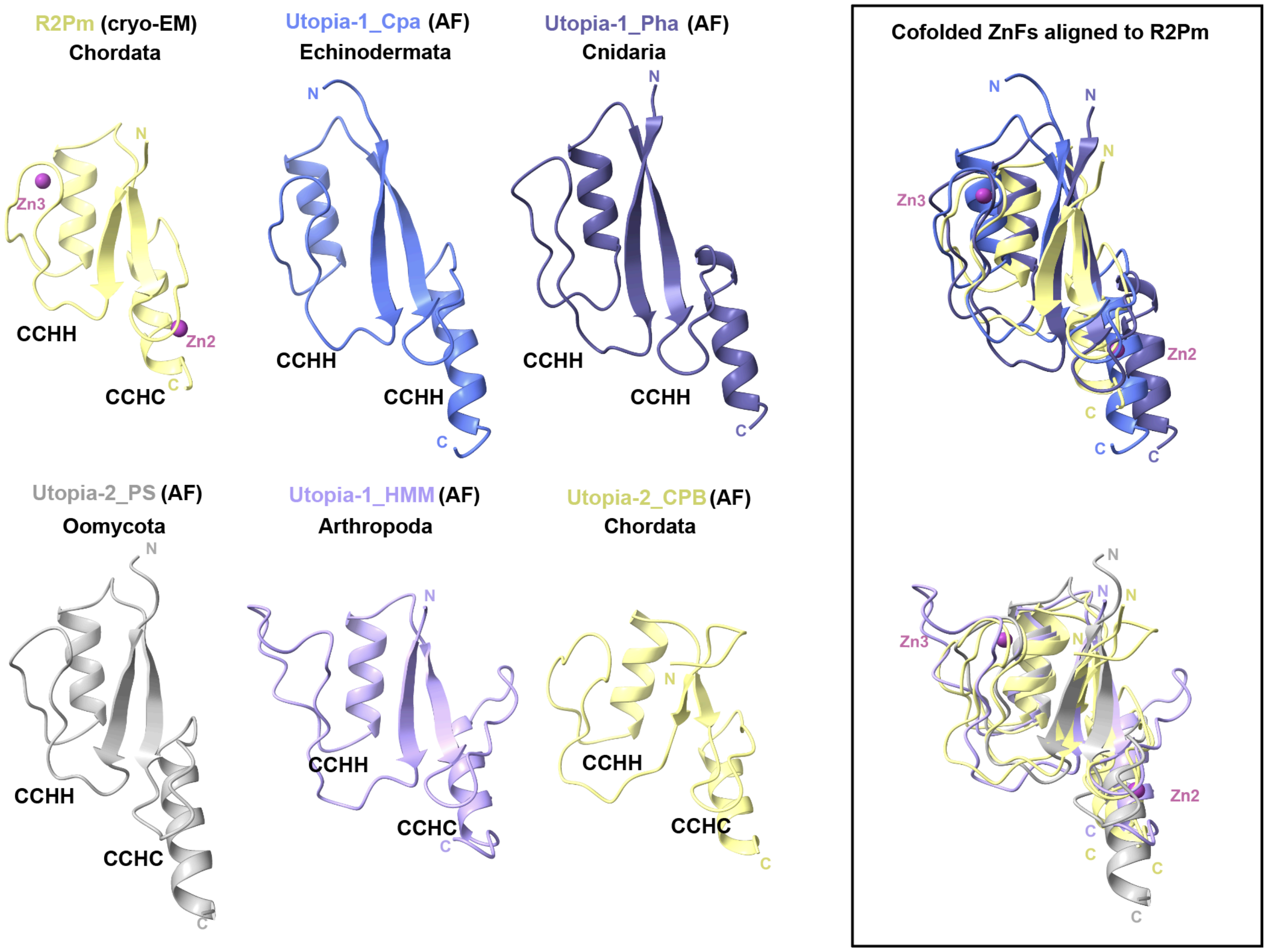

Figure S9: Cofolding ZnFs are a feature shared among R2 A-lineage and expected for some Utopia. Protein segments that encode for cofolding ZnFs are displayed, which correspond to ZnF3:2 in the experimental structure of PlaMe (R2Pm) and ZnF2:1 of all Utopia elements indicated (AlphaFold models, AF). Individual protein folds are coloured according to the phyla discussed in the main text. Structural alignments for each row relative to R2Pm ZnF3:2 fold are made on the right (boxed), where the top panel includes three structures and the bottom alignment a total of four. The names of the host species not provided in the main text include those annotated by us, *Crossaster papposus* (Cpa), *Phenganax stokvisi* (Pha), and those previously annotated [12, 21] *Phytophthora sojae* (PS), *Heliconius melpomene* (HMM), *Chrysemys picta bellii* (CPB).
