# Supplementary material for: Lineage-specific evolution, structural diversity, and activity of R2 retrotransposons in animals
Source: Genome Biol. 2026 Apr 14;27:174. doi: 10.1186/s13059-026-04073-3 (PMC13188248; doi:10.1186/s13059-026-04073-3)
Supplement: Supplementary file 10 — Additional file 10. Position-specific scoring matrix plots extended. [file 13059_2026_4073_MOESM10_ESM.pdf]

Additional file 10

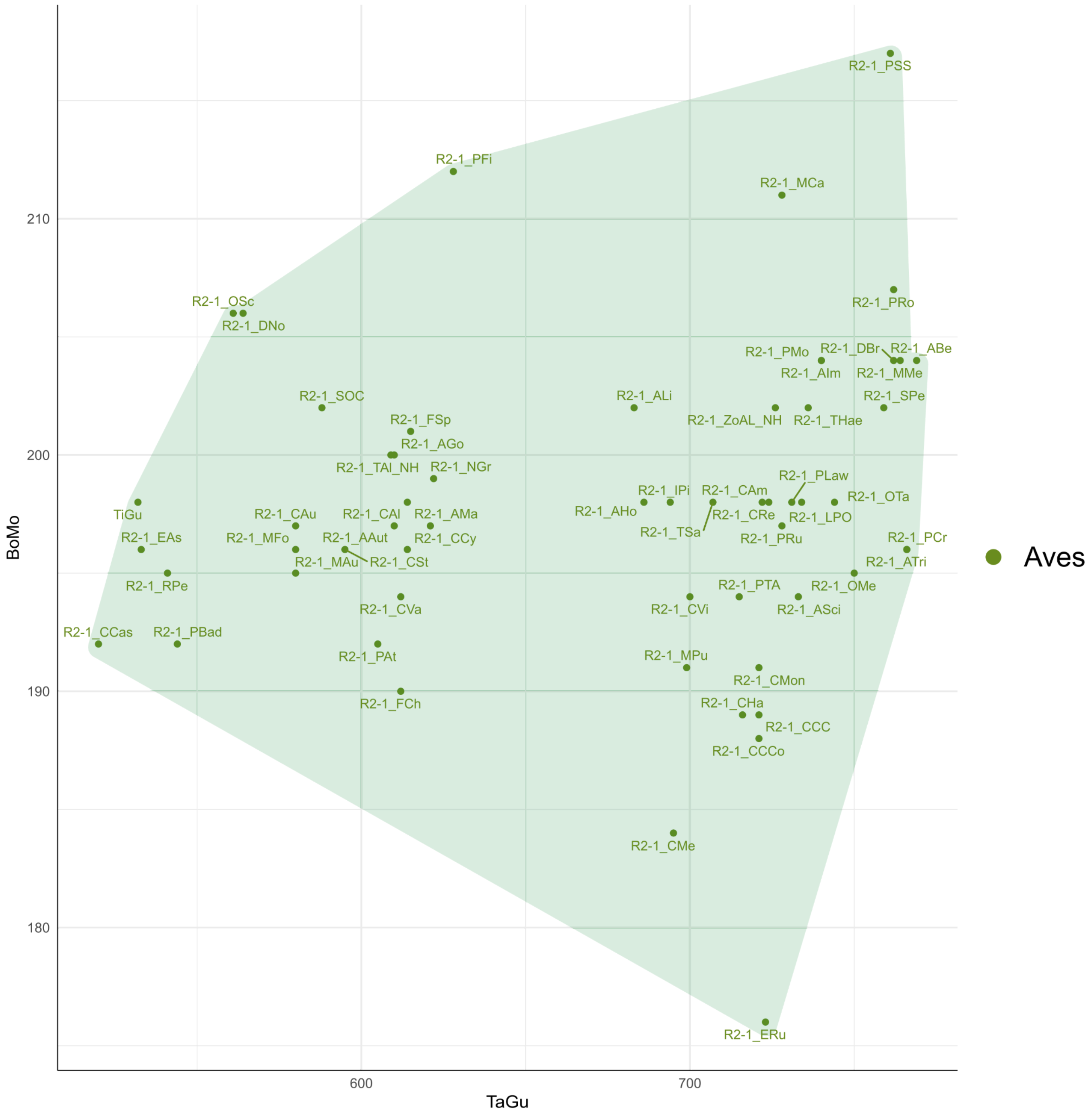

Figure S10.1: Position-specific scoring matrix scores of Aves R2 RTs compared to BoMo (*B. mori*) (lineage D R2) and TaGu (*T. gutatta*) (lineage A R2) RTs. Green shading indicates the R2 has 3 N-terminal ZnFs.

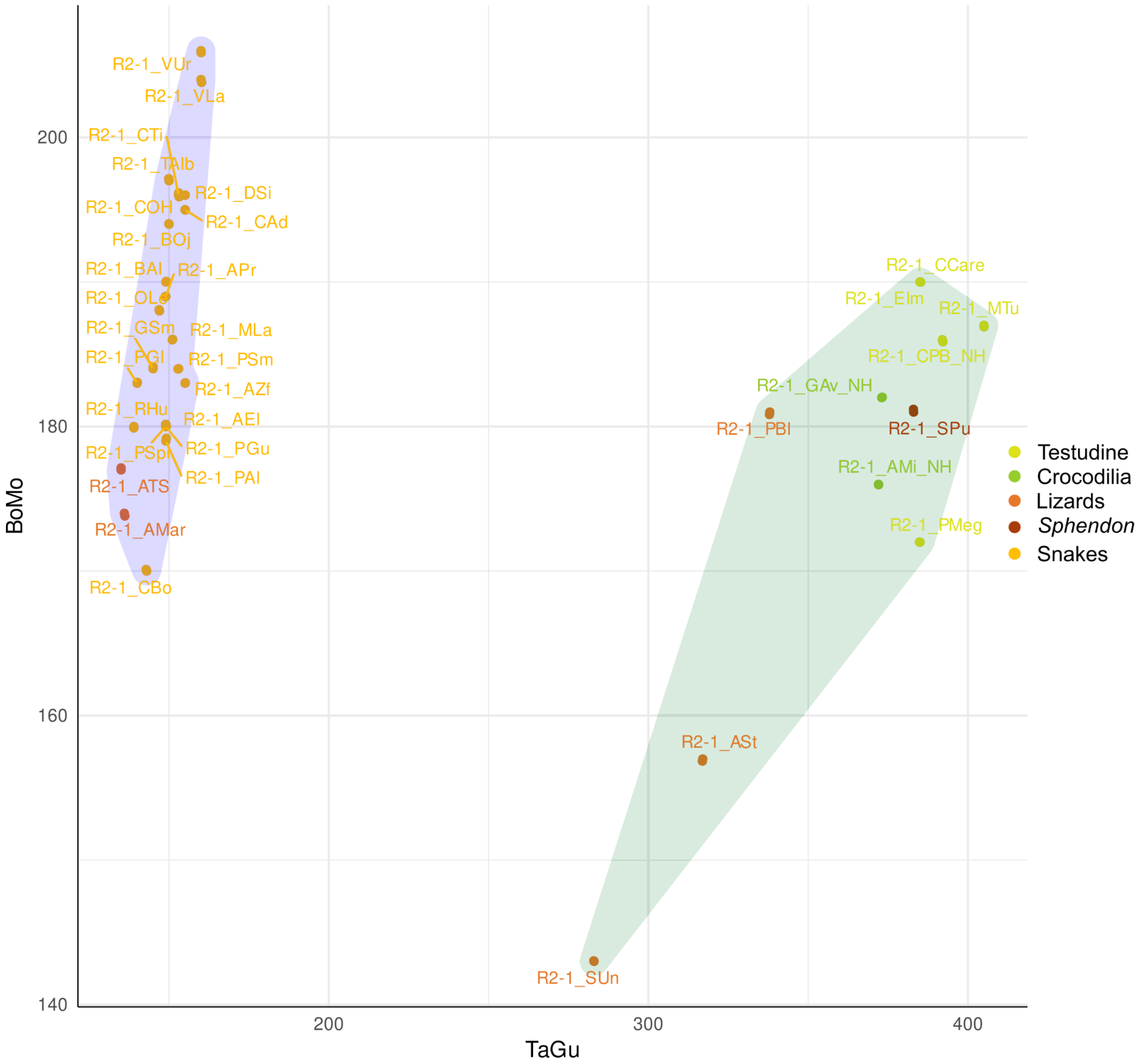

Figure S10.2: Position-specific scoring matrix scores of reptilian (excluding aves) R2 RTs compared to BoMo (*B. mori*) (lineage D R2) and TaGu (*T. gutatta*) (lineage A R2) RTs. Green shading indicates the R2 has 3 N-terminal ZnFs, purple has 1 N-terminal ZnF.

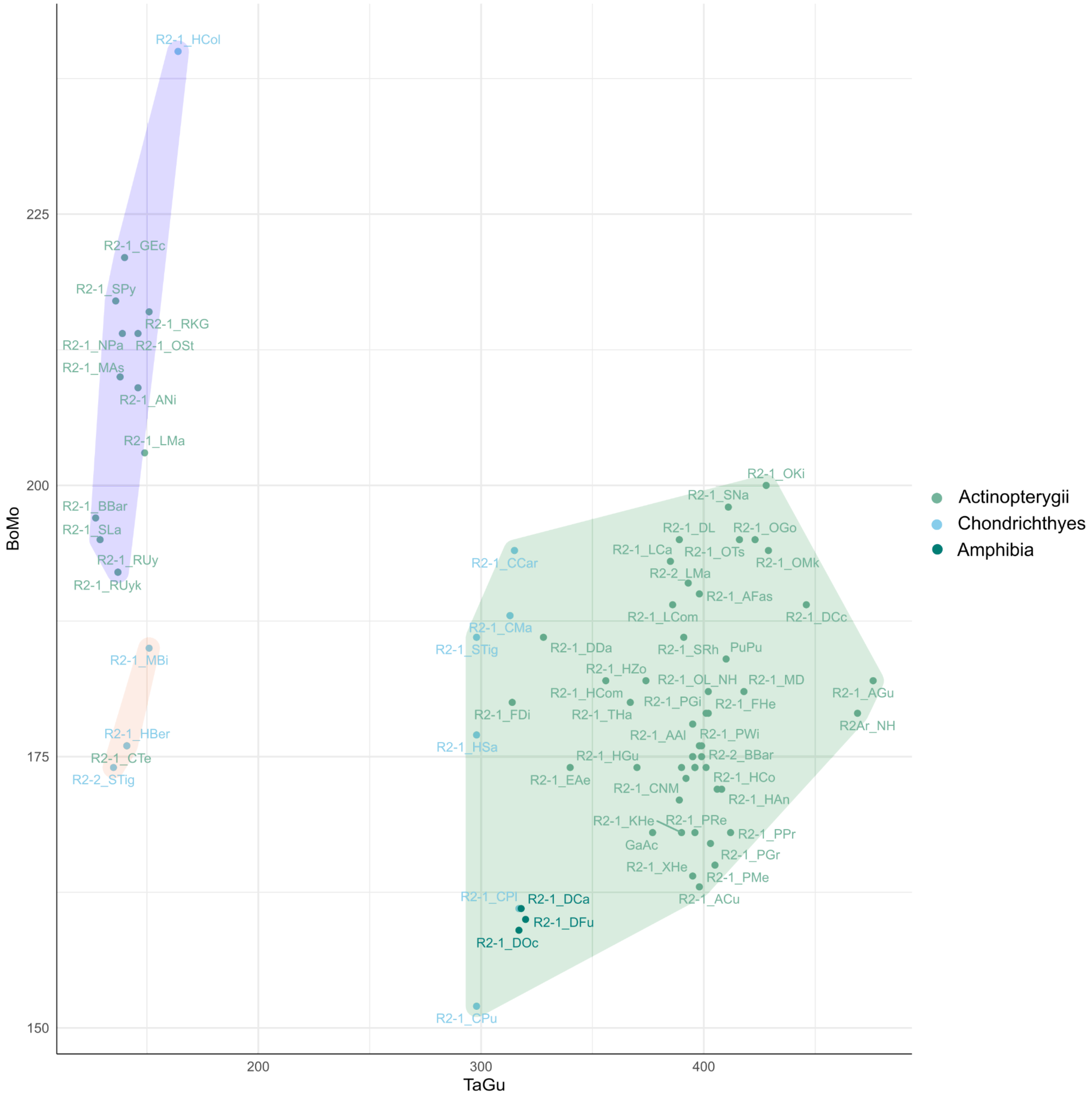

Figure S10.3: Position-specific scoring matrix scores of Actinopterygii, Chondrichthyes, and Amphibia R2 RTs compared to BoMo (*B. mori*) (lineage D R2) and TaGu (*T. gutatta*) (lineage A R2) RTs. Green shading indicates the R2 has 3 N-terminal ZnFs, purple has 1 N-terminal ZnF, and orange has 2 N-terminal ZnFs.

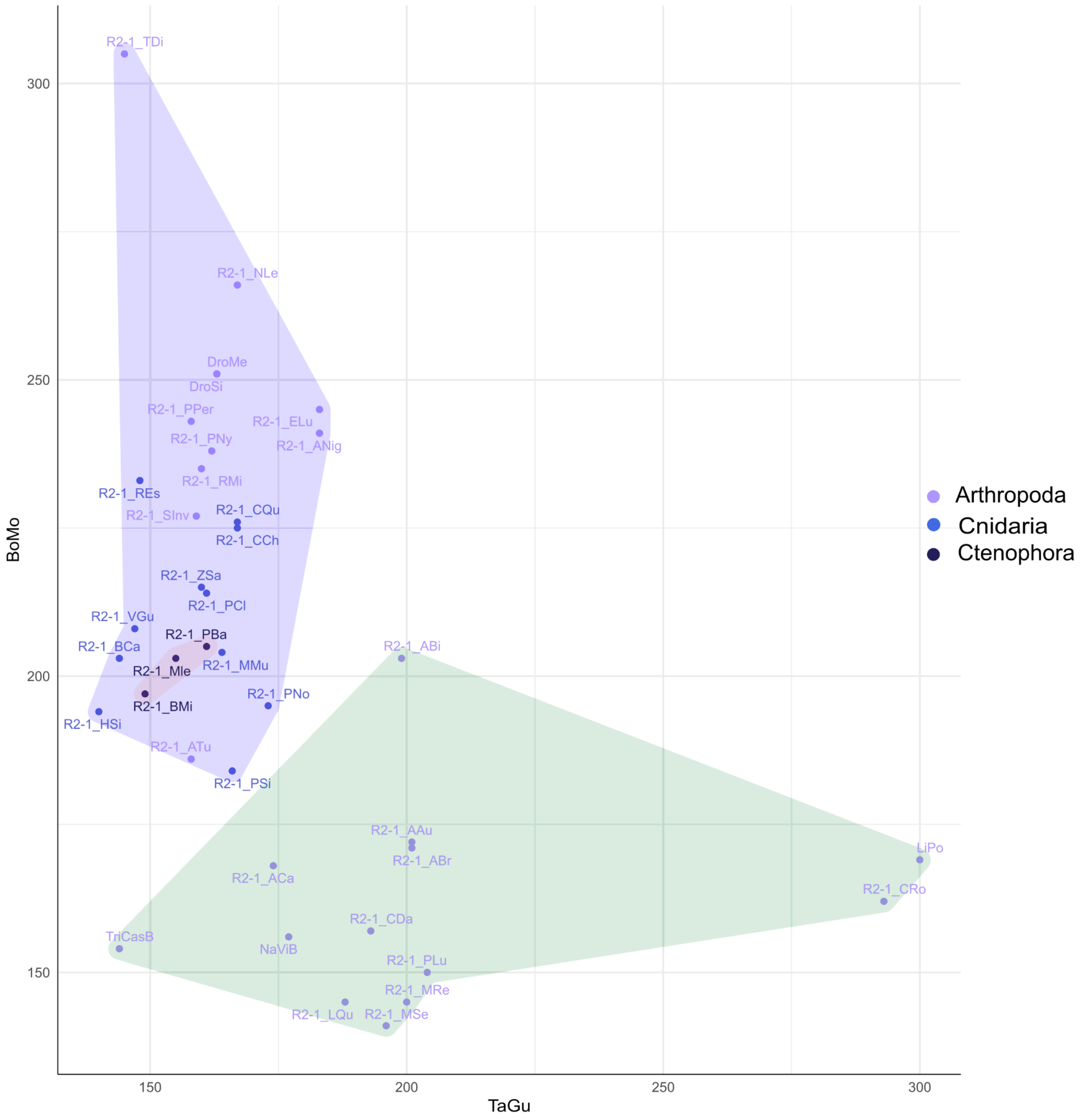

Figure S10.4: Position-specific scoring matrix scores of Ctenophora, Cnidaria, and Arthropoda R2 RTs compared to BoMo (*B. mori*) (lineage D R2) and TaGu (*T. gutatta*) (lineage A R2) RTs. Green shading indicates the R2 has 3 N-terminal ZnFs, purple has 1 N-terminal ZnF, and orange has 2 N-terminal ZnFs.

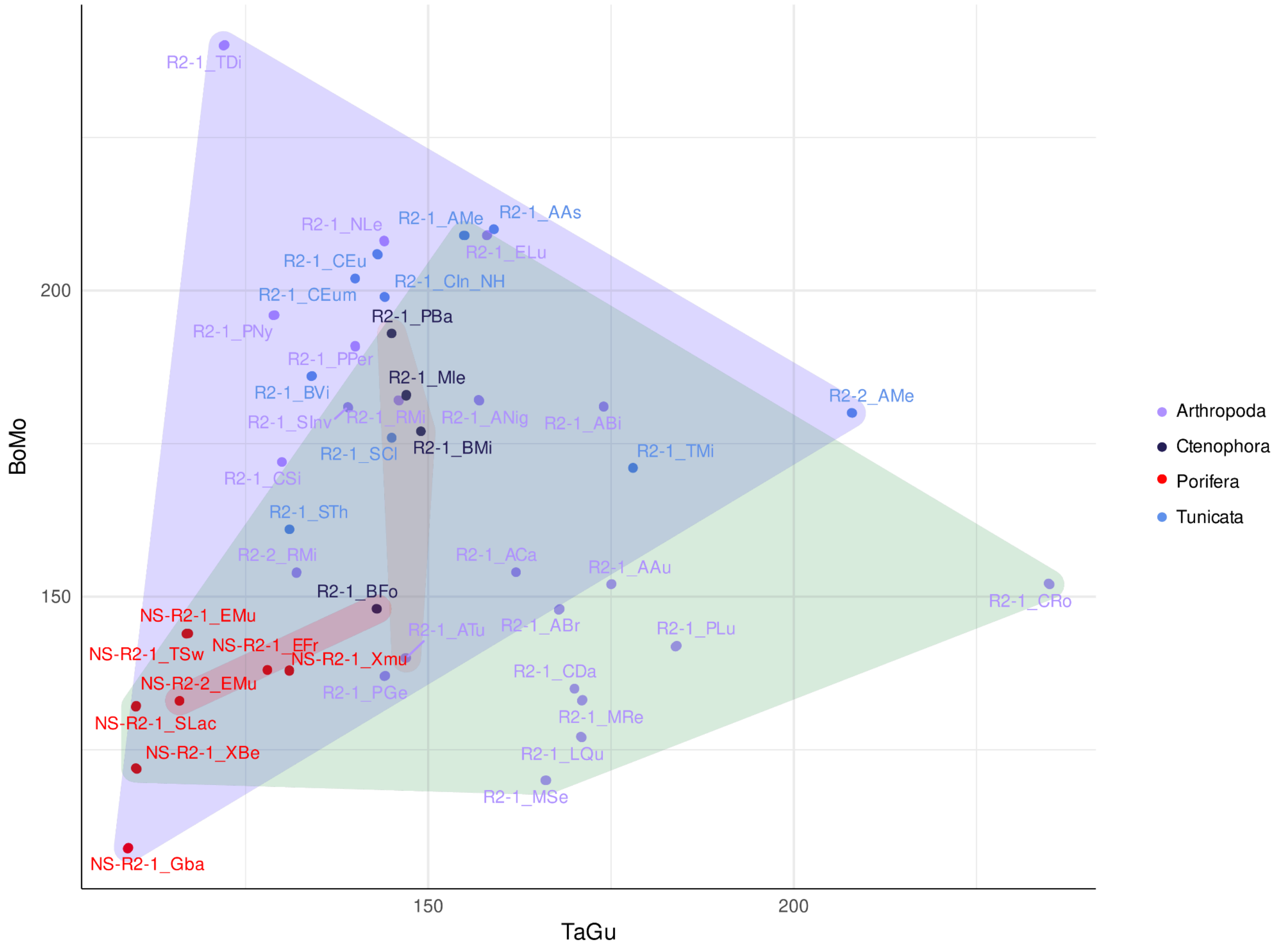

Figure S10.5: Position-specific scoring matrix scores of Ctenophora, Porifera, Tunicata and Arthropoda R2 RTs compared to BoMo (*B. mori*) (lineage D R2) and TaGu (*T. gutatta*) (lineage A R2) RTs. Red shading indicates the R2 has 4 N-terminal ZnFs, green has 3 N-terminal ZnFs, purple has 1 N-terminal ZnF, and orange has 2 N-terminal ZnFs.
