# Supplementary material for: Lineage-specific evolution, structural diversity, and activity of R2 retrotransposons in animals
Source: Genome Biol. 2026 Apr 14;27:174. doi: 10.1186/s13059-026-04073-3 (PMC13188248; doi:10.1186/s13059-026-04073-3)
Supplement: Supplementary file 11 — Additional file 11. Site-specific conservation of individual residues in lineage D and A R2s. [file 13059_2026_4073_MOESM11_ESM.pdf]

Additional file 11

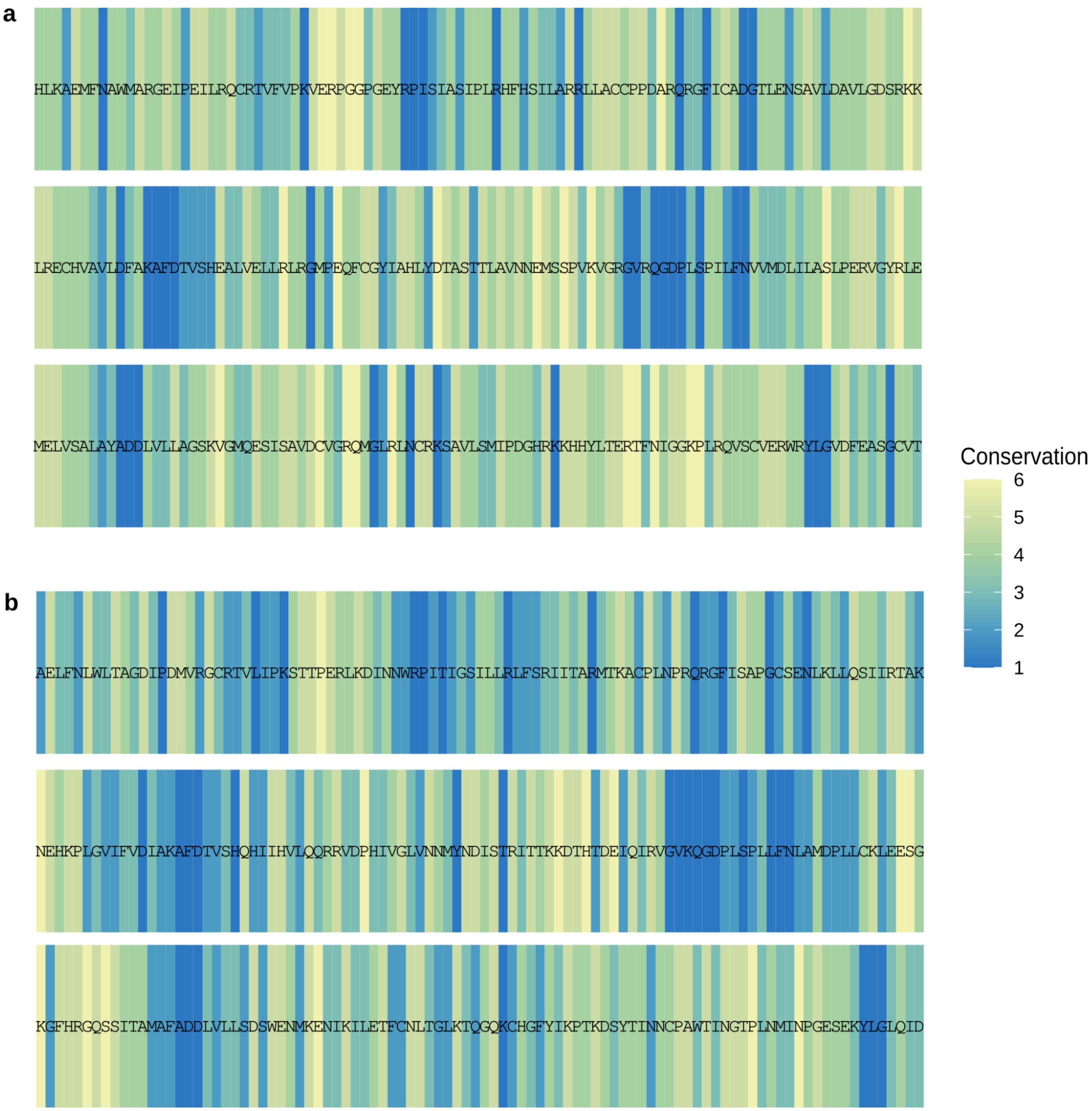

Figure S11: Site-specific conservation of individual residues in lineage D and A R2s superimposed with RT residues from (a) *B. mori* (lineage D) and (b) *Z. albicollis* (lineage A, reconstructed from short-reads). Conservation is shown as high (1-3) to low (4-6).
