# Supplementary material for: Lineage-specific evolution, structural diversity, and activity of R2 retrotransposons in animals
Source: Genome Biol. 2026 Apr 14;27:174. doi: 10.1186/s13059-026-04073-3 (PMC13188248; doi:10.1186/s13059-026-04073-3)
Supplement: Supplementary file 12 — Additional file 12. Multiple sequence alignment of R2 proteins selected for activity testing. [file 13059_2026_4073_MOESM12_ESM.pdf]

# Additional file 12

```
LaMa       ------------------------------------------------------------
NePa       ------------------------------------------------------------
CroHe      ------------------------------------------------------------
DeFu       EPSSSKKHPVH-TNATMALPD--AYEVPREQKQLKADIKKKIIYWSYPSPFRCNLCGDIM
AgTri      --------------AGEGTPEALPPVPGENGGQSPDNIVKVTVPDKNPP---CPCCGTRV
GaGa       ----PESAPVQRTEADTTLPS--ASAPAGEGEPPSTRVFLVRLPDSNPP---CPICGDHV
SPun       RPIHPEEESAICVTANESTPQIEYNIIERETDVIPSSSAIVQLPQTNPA---CPFCADRI
PlaMe      EPI--------------------INGTQQK--------TIIQLPNDNPA---CPFCGDHV
PBla       ----------------------------KRERKLDKDEPTIWLPDNKPE---CPYCKEVV
                                                               ****
```

```
LaMa       -------------------------------------------------------DNITC
NePa       -------------------------------------------------------ERVPC
CroHe      -------------------------------------------------------PKHSC
DeFu       STIKELKRHISCKHKTFNLVVFCSKCDKEDA-FHNIACHFAKCNKKIIED-----ATFMC
AgTri      NSVVSLLEHLKGSHGKKRVRFRCAKCGKENVNYHSVICHFPKCKGAEIEK--VPAGEWIC
GaGa       GKPSALAVHLVESHAWADVQYQCTHCEKVSSNKHSILCHIPRCQGRVTDR---SDRNWAC
SPun       TTPALLKKHVKGYHGGKLLQFQCSKCGRLNENQHSIITHLPKCKGNSIDP---ALGEFHC
PlaMe      GKPSALNVHLKRNHGGREVEFQCSMCNKADPKAHSILCHIPKCKGKVTEE---PTGDWAC
PBla       GKPTALQKHLTGVHGNQAVTFGCMKCQRKDPRVHAILVHIPKCKGPNIPDARQAQNKHRC
                                 ****                                 *
```

```
LaMa       RDCGRAFPSLRSLGMHRHHQHPEEVNEERLELIKKK--------RKQWSKEEDEQLMSTA
NePa       -FCGLTFATKRGLSMHQHHRHPTELNAARLTALPSR--------RGVWTSGEDEALIRLA
CroHe      -PCGSEFGTKRGLAMHRFHCHPAEVNAERLTTLPTK--------KLVWTQEEMDGLLRHA
DeFu       RHCDERFTTKSGVSQHARHRHPKAVNDIRLKIAMPA------KRSKLWTLEETLLLMDLE
AgTri      EVCGRDFTTKIGLGQHKRLAHPLIRNQERIVASQPKETSNRGAHKRCWTKEEEELLIKLE
GaGa       VQCPASFNKKVGLSQHKRHVHPVTRNAERVAGSLSRAGLRPRTRRGCWSVEEEETLTRLD
SPun       EICNNNFLTKSGLSQHKRIRHPLVRNEERIMASQPKENSLRGKHKSCWSNDEVEQLKTLF
PlaMe      ETCNKQFNTKSGLSQHKRIAHPAIRNQERIAASQPKPNSQRGKHNSCWTVEEEQLLAAFN
PBla       EDCGNQYDTQSGLSQHTRHKHPETRNKQRIEEKEKEKGGKRGTHKSCWTQEETEQLALLW
           ***   : .  .:. *    **   *  *:                 *:  *   *
```

```
LaMa       DMEWKVGMMKKDHLAAIRIKFSHRTLDSIKKRLQHLGWAPPPAGQQPQNTPLPARSRPPR
NePa       NDIWPSVTLKKHLYNALMPHFPGRSAEALKKRLQLLQWLP--------------------
CroHe      NGAWKEGMLKTQIFTSLMPHFPGRSAEAIKKRLQSLKWVP-------------ARDNPER
DeFu       ATYKN----SKKINMDIQKHFENKTSIQIGEKRRELREKT-------------AKMEKEA
AgTri      AQFEG----NKNINKLIAEHITTKTAKQISDKRRLLPKKL-------------PEVTDKE
GaGa       ALFRG----ARNINQLIAAEMGTKLPKQISDKRRQLGLCP-------------EQATSGG
SPun       DEFGK----YKDVNKRIANLLPSKTAKQIADKRRLLNLYI-------------KSTKTPN
PlaMe      NMFWG----KKNINILISDHIHMKTAKQISEKRRLLGLNK-------------NATVTTT
PBla       EKYEG----HANINKLIQQELHTKTAKQIGEKRRNILEKR-------------NNKPEIG
                      .    :   :  :    : .: : :
```

```
LaMa       RIARASSPPLT---SALAGPLPPSPIGSPPPQAKRRGALWTNREDSELEEHARRLWKPNM
NePa       --------PL----TPSHETLNASAL---SP-----------------------------
CroHe      -----NSQPL----HATSGNVAPATLPTPDP-----------------------------
DeFu       -----NL-------HIDSDDEIESINKSEDE-----------------------------
AgTri      -----PGVCLRTRRAAGSVGTEPGTSQQSQA-----------------------------
GaGa       -----DA-------ESTSVVEEESVTPEVET-----------------------------
SPun       -----PI-------EPET--QEVTIPENRES-----------------------------
PlaMe      -----NP-------LPVS----STCHLKIRT-----------------------------
PBla       -----EE-------IPKT----KTQSINRQT-----------------------------
```

```
LaMa       LKKSLAEALSCIIKGRSAEAIRKRLQKLGISSATMTSREDIDSEAMRDKDIHQTTPCVQP
NePa       ------------------------------------KALD--------------------
CroHe      ------------------------------------SRVD--------------------
DeFu       ------------------------------------EHTN--------------------
AgTri      ------------------------------------GILD--------------------
GaGa       ------------------------------------QSPL--------------------
SPun       ------------------------------------SAPV--------------------
PlaMe      ------------------------------------DSPN--------------------
PBla       ------------------------------------------------------------
```

```
LaMa       EEDGGYETPTSKKEEWWRTLLTKALETL-NEARIESDKL-------------------KL
NePa       ED-------LI---RWKGRMLDAISPNL-SEPRLGTEEL-------------------LS
CroHe      DQGANIEDPAA---SWRRALLAAAADHF-KEPKLGAGTL-------------------KQ
DeFu       VPQSDVQI------RNTRISTHKKSPQI-KDHKDLVERITEIKDCMKNPEYYDCGIGLME
AgTri      NGPGKCHL------PGRPAAGEKTMEKL-RRHPDKDNGR-------------------QK
GaGa       KPPGKI----------RKVLAQRARRWL-KKGQDLSDKV-------------------RE
SPun       --KGLC-----------EALQSKARKNLEKESAMKFE---------------------KD
PlaMe      TTTGLK-----------DTYMCKINENIVNQGQIKFD---------------------SE
PBla       EKEGLA-----------NKFKQTAITLI-QEHRIKNESI-------------------AR
                                      :
```

```
LaMa       MAKGMLEG---TMTREEAAEQLECIT----------------------------------
NePa       LVCNLRDG---SLSSEELNARLEAHA----------------------------------
CroHe      ITQDLLNG---KISSGECRVLMGEHA----------------------------------
DeFu       VITQYIT-----ENISTIKPSASVRPTTDDPAAPNDATE-IEV-----------------
AgTri      TSGQRREGLLQAHYQKTIKEGLSAGAINNFPGAFKQLMDGREMRTMINQTAQDCFGCLES
GaGa       VLGAWVEG---QPGIRARVESVSLDVLTSFLGAPSG------------------------
SPun       FLTNWLDN---MQNVRHLLEETTVDALCNFLPEPKN------------------------
PlaMe      VISAWMAG---DSNIRSLVESTSLDILSTFLMETPK------------------------
PBla       ALHTWLQE---NNNTRQTVEKATEEILNNFPRTAG-------------------------
```

```
LaMa        -------QEFAPLKWKPREKRTGHR--KEPRSNKQIRRARYAHIQRLYKLKRKDAAHSIL
NePa        -------ARHFPHLWRPSTKRTQSI--NKP-SARQIRRANYAAIQRLYQTQRKDAASSVL
CroHe       -------QHWFPHTWKPSSARPEPK--ARQWKTREIRRANYAAVQKLYKLRRRDAAQAVF
DeFu        -------PTNVPQNAEPTTASSSHKRNSGHKKRYAKRKGSYKEYQTMFLKDKKKIARIVC
AgTri       ISQIRTAMRGKNSGKKATMEQPARKF-QKWMKDRAIKKGNFLRFQRLFHLDRGKLAKIIL
GaGa        -------PRGAPDKKRP--EKGGST--TSWMSRRAVKRGVFLKYQRLFGTKRKLLADIIL
SPun        -------ITKPKKDRRP-VKDRKQA--KSWMKKRAKKRGLYQHYQSLFLKNKTRLASIIL
PlaMe       -------PRKKGNNKIT-NKKSGKK--KKWMEKRAVKKGFYKRYQHLFETDRCKLASIIL
PBla        -------IHNIPKRKKTKKPRGPYR--GKWRQKRRIQKLTYKQLQELYDKDRAAAAAYIL
                            .              .    ::  :   * ::   :   *  :

LaMa        DGKWREAYREKEREVEGVEEHWAQVFETQAK-DGWPQSM--LPPDLSNIKWGLLEPVTAM
NePa        NGSWRSAYKNRTSITDDLDSYWSNIFLQPSHEDPT------PADPPSTPHWSILDPITAT
CroHe       SGSWRNAYRGRAPQPDKMDQYWAEVFETASATDVT------PPGSPSEIYWSVVGKITPS
DeFu        DGAEDMSCQ---IEATEIFDYYKNILENENTKNPKFELSNIVPSEEDFDHLN--RPITEF
AgTri       DDIECLSCD---ISPSEIYSVFKARWETPGNFAGLGDFK--ITGKADNNAFK--DLITAK
GaGa        DGADRAQCV---LPLEEVLRAYRGKWEVESSFEGLGRFG--VRRDADNFAFK--ALITPE
SPun        DGTEKFECE---IDPKIVYEAYKNKWESATEFKGLSNFH--SFDVTDNSKFY--TRISGK
PlaMe       DGTERLQCQ---IPLTEILETYKSKWETLTPFEGLGQFK--SHAVADNTAFE--ILLSAK
PBla        DGEKKTNCD---LPIQEVYQTYKDKWETKTEFKGLQNFH--PWGGTNNDILS--NLITGE
            ..               :   :                        .         ::

LaMa        EVEVALRSM-NNTSAGMDKLSAQEVLTWDL---PSLAGLLNVILATERLPSTLATARVTL
NePa        EVTSALSSM-KSSATGLDRLSASQLLSWDA---NSIGAYFNILLVSGLVPTHLTMARITY
CroHe       EVANALKGM-RNSAPGLDRITAEELLTWDH---PSLAAYYNLMLAAGGPPEHLACSRVTF
DeFu        EITWHLENSNMNKATGPDNVGLKELFGLHARDHTILTDLFNIWLQTSKVPECIKRNRSIL
AgTri       EIERNVQEMSKGSAPGPDGITLGDIVKMDP-GYSRTAELFNLWLTSGEIPDMVRGCRSVL
GaGa        EVVKHMMAMASKSAPGPDKLTLRDLRRADP-EGDALAELFSLWLITGTVPDGLKECRSVL
SPun        EVQKNIKEMSRKTAPGPDGITVEDLENVDP-DGEILAALFNLWMAVGIIPDTIKECRSLL
PlaMe       EIMKNIKEMNKNSAPGPDKVSLRDLLLADP-ECNALEKLFNTWLITGIIPNSIKECRSLL
PBla        EVKEHLQAMCNKTAAGPDNISVKDLKQVLH-VEQKLAELFNLWLITGQIPNTVKKSRSIL
            *:   :      .:.* * :   ::               .  :     *  :   *

LaMa        IPKVEEPS---GPNDYRPIAISSVIARALHKVLSKRMREQFEFSPLQYAFLQRDGCLEAS
NePa        VPKTDSPS---GPSEYRPISVTSVLLRAMHKILARRMLATLDFSDLQLAFLQRDGTLDAS
CroHe       IPKVENPQ---TPGDYRPISVASTVLRAFHKILAWRLRDNLQLSPFQHGFLQRDGCLEAT
DeFu        IPKNVPLDSLGNIGNWRPITIGTALMKLFTKLLTKRLSTFVSIHERQKGFINARGCLENL
AgTri       IPKSTKPERLKDINNWRPITIGSILLRLFSRIVTARLSKACPLNPRQRGFIRAAGCSENL
GaGa        IPKTVDREKLGQLGNWRPITIGSIVLRLFSRVLTARLAAACPINPRQRGFIAAPGCAENL
SPun        IPKTSDPEKLKDIGNWRPLTIGSIIIRLFSRILTIRLAKACPLNARQRGFIDSPGCSENL
PlaMe       IPKTADPEALKELGNWRPLTIGSIVLRLFSRIITNRLAKACPINARQRGFIATPGCSENL
PBla        IPKTSDETEKKKVGNWRPLTISSVVLRLFSKIMTSRITKACPLNNRQRGFIAASGCSENL
            :**          .::**::: : : : : :::: *:     :   * .*:   *  :

LaMa        ALLHAVLRTAHERTKPLAAAFLDVSKAFDTVSHNAILGAAEKAGTPPPILRYLNQLYKNA
NePa        TILHTILRKVHTELKPLSMMFLDVSKAFDSISHHTLIRVATTSGLPAPLLSYLRHLYQTS
CroHe       ALLHTILRKVHNSRKSCAMLFLDVAKAFDTVSHQTLFRVAVELGLPPPLVNYLKCLYSRS
DeFu        NILKNSIKGARKNKDSLAVIFIDISKAFDSVGHKHILNSLKRLHVPLGYRQLIKDLYTNS
AgTri       KLLQTIIRTAKSEHKPLGVVFVDIAKAFDTVSHQHILHALQQRGVDPHIIGLVNNMYKDI
GaGa        KVLELLLRKRKRDRQPLGVVFVDLARAFDSVSHDHISWVLKAKGVDEHIVNLIEDSYQKV
SPun        KSLQSIIEYSKKEKQQFGVVFIDIAKAFDSVSHDHIIWVLKERKVDEHIIKIIQDSYTKV
PlaMe       KILHTIVKQAKTSKKSLGVVFVDIAKAFDSVSHDHIMWVLQERGLDQHIVNIIEDSYKKI
PBla        WLLHNIIKRAKDKKKELGVVLVDIAKAFDTVSHDHIRWVLRERGMDKHIIQLIMSAYDNA
              *.  :.  :   .  .  ::*:::***::.*. :                :   *

LaMa        KLQL------GSTTTQCSRGVRQGDPISPILFILVMGEVLEEALPD-VGVRW----GERQ
NePa        KIRL------GTHDSSCGRGVRQGDPLSPILFILAIEDILHRVLPE-AGFDL----AATR
CroHe       TVRL------ADKATKCGRGVRQGDPLSPLLFIMVMDDIVRKTLPE-VGFDL----DGQR
DeFu        TTTFKGKNNINTDEIHMKSGVKQGDSLSPLLFNIAMDPLICDLQYKGCGFSFNNIQGTRQ
AgTri       STYVTTKRDTHTDKIQIRVGVKQGDPLSPLLFNLAMDPLLCKLEESGKGFHR----GESS
GaGa        TTRVQVFNG-VTPPISIKTGVKQGDPMSPLLFNIAMDPLIAKLETDGQGVKV----GSAS
SPun        STRLKVSKT-LTDSISLKVGVKQGDPMSPLLFNLAMDPLINALEEQGEGVQV----EDMK
PlaMe       HTRMEVGTE-RTPPIEIKVGVKQGDPMSPLLFNLAIDPLITALEKANTGFSY----GKNK
PBla        TTSIKLKEG-NTPDIHIRSGVKQGDPLSPLLFNLAMDPLITELETEGHGVED----ENWT
               .               **:***.:**:** :.:  ::        *.

LaMa        IDSIAYADDLILLAESPRELQRKLDGVCRGLQKAGMALNNKKSVTMTILKDGRRKTLALA
NePa        IGSIAYADDLILLAERPERLQEKLNILLSAFHDAGLIINSSKSHGLTIAKDGKKKLLVLL
CroHe       VDSLAYADDLVLLAEKSPRLQDKLHLLSEALRKAGMSLNARKSRGLTITKVSKRKQMVIT
DeFu        TTALAFADDIAVLSNSWKGMQANLQIIEKFSKATGLKLNVKKTHGFLISHF-GDKIVVNK
AgTri       ITAMAFADDLVLLSDSWENMQTNIKILETFCDLTGLKTQGEKCHGFYIKPT-KDSYTINN
GaGa        LTTLAFADDLVLLSDSWEGMLKNISILEDFCNLTGLRVQPKKCQGFFLNPT-CDSFTVNN
SPun        LKTMAFADDLVLLSNSFEGMSKNIKILEQFCQTTGLQVQPKKCQGFLITPT-KDSYIINN
PlaMe       ITSLAFADDLVMLSDTWEGMNKNIQILETFCNLSGLKVQAKKCYGFFLSPT-HDSYTINK
PBla        ITTLAFADDIAVMSDSCKEMKQNLQIIEAFCNLTGLKVQTTKSYGFALQPT-KDSYIVNN
              ::*:***: ::::    :  ::  :      :*:  :  *   : :      .

LaMa        PHQYTTDNGQVPCMGLSDSQRYLGIQFT-WKGRVTPK-QTSELGRMLLEITSAPLKPYQR
NePa        PHEYRTGSSTIKPIGPTNVITYLGLRFN-WKGQLLPR-HTATLSTMLAEVSQAPLKPYQR
CroHe       PTTYECEGEPIKPMGTDDSVRYLGLHFN-WKGRIVPK-HTGKLDSLLKELTKAPLKPYQR
DeFu        CKPWLFERSKIEFIHPGESERYLGLNFDPHIGCNTPNALNLKLQSWAKKLDDLPLKPTQK
AgTri       CAAWTINGTPLNMINPGESEKYLGLQFDPWTGLAKTN-LTTKLEFWLERIDQAPLKPLQK
GaGa        CEAWKIAGREITMLGPGESTRYLGLNVGPWVGIDKPD-LGTQLSSWLERIGTAPLKPMQK
SPun        CKKWSIEGTEVNMIQPGQREKYLGAKIDPWTIFAEIN-FEEKIEDWLCKLEVAPLKPSQK
PlaMe       CDAWKIDKDSLNMIQPGESEKYLGLKVDPWIGFSKPV-LAEKLTIWLKRLTEAPLKPSQK
PBla        IKPWTIKDQPIQMVQPAHTTRYLGIQVGPWKGMEKPN-MIADTKKWLQNITKAPLKPTQK
               :      :  :   .   *** ..                     .:   **** *:

LaMa        IELVRDFLVPRLLHELVLGCAHRNTIARMDRMIRRETRTWLRLPKDTSLGFLHSPVKSGG
NePa        LEVLRNYLIPKLTHELVLGRAHRNTLKKIDVLIRAAIRQWLRFPKDTPTAYFHARIQDGG
CroHe       LQLLKFHAVPKFTHELVLGHAHRNTVKKLDCLTRAAVRKWLRLPQDTPLGYLHANVKDGG
DeFu        IKIFCQYIVPKLSYSLEMTGTGANTLVALDQTIKATVKHYLHLPHYINDGLLYSRKKNGG
AgTri       LDILKTYTIPRLTYLADHSEIKAGALEALDQKIRTAVKDWLHLPPCTCDAILYSSTKDGG
GaGa        LSLLVQYAIPRLNYQADYAGIGRVALEALDSMNRRKVKEWFHLPACTSDGLLHSRHRDGG
SPun        LEILNIHTIPRIIYLADHTNCNITKLKNLDNMIRKRLKDWLHLPASTCHGLFYSKNRDGG
PlaMe       LTMLNIYTIPRIIYLADHTDTKKTLLSSLDDNIRTVVKGWLHLPPDTCNGFIYTKTRDGG
PBla        LEILRTYTIPRLIYHNEQTGTGKTKLKELDNLIRAGIKSWLHLAHDTCTNLIYTKTKDGG
            : :.  . :*:: :           :  :*   :   : ::::.       :::  :.**
```

```
LaMa         LGIPCLGTTVPLLQKRRFEKLLSSDCPIKQTLTELPSFMTTLRRVNL-------------
NePa         LGIPALTTQMPFLQHHRFTKLLSSASPSIQSICQEPAFVQAYARVLT-------------
CroHe        LGIPCFSTSIPLLQKKRFEKIATNPATIFQIMQRQDSFRTQGRRLDK-------------
DeFu         LSIPKLCTSIGINKVNNLLYLRESTDVCITNSFLYAGIV-------------------IN
AgTri        LGVTKLAGLIPSVQARRLHRIAQSSDETMKEFLEKDQMEQLYRKLWVQAGGEREEIPSIW
GaGa         LGLPRLAKAIPEAQVRRLIRVATSSDEVTRKVSYACGISDEVERLWLARGGDMSSVPRFE
SPun         LGIPRLERLIPSVQARSLHRISQSSDYKIMNIAFSCGLESEFEKKWVQAGGAKETRPNLK
PlaMe        LGVTRLASLIPSIQARRLHRIATSEDETIRNIAMANNIEEEFQNLWVTAGGKKEEIPRIT
PBla         LGITRLETTLPHQRCNTLLKIINSPDLTTRTIARALGIEEQFSKWWKKAGGSEDNKPKIY
             *.:. :   :   : . :  :  .             :

LaMa         -----PCRVRNEIV--------------------------CSSKEAQVEWEKIWRTSAD
NePa         -----P---TLPIF--------------------------ESKSAIKQHWADQLLQSID
CroHe        -----PCRLNSTVV--------------------------TSKTEVREEWGNMLSNSVD
DeFu         DVIDEIIKTHH------LHILHA-ECLNMSLELTASY----NWRESEFNRWKDM---VVQ
AgTri        E----ALPTSEQSNRG--DTTSEWEAPILKPKFPKPC----DWRKKEFEKWTKL---VSQ
GaGa         D----PEAPRSPGVQGPCEAAQEIPSVVRKLAIPRPS----NWRAEEHSKWAQL---SCQ
SPun         DILPPPPEPKY------YEKLNEWEKPKLKIKFPLPL----NYRKEEFREWAKL---KCQ
PlaMe        D----PVSIDYRLPRRILELLNEWEKPAPKKMYPIPC----NWREAEMAHWKNL---PCQ
PBla         P-----------------QPQNTTQKPTEKEEIPVTTKIQPTHKKKEAEEWAKL---PCQ
                                                              .* .      :

LaMa         GRSGVNEDVDPASYTWVGKPRRVFPRIHLRGIQLRGGTLNTKARASRGRETAPSDIACRG
NePa         GRDLAQTDIDVASHWWIQHPENVFPRLHLRGLQLRGGLLSTKTRNNRGPTRQ-ANVSCVG
CroHe        GRELRHPEVDKASHEWLLRPDRVFPRLHMRGIQLRGGLLPTKARSGRGNNRQVTSKTCRG
DeFu         GDGVQYYQNNACSNYWLMKPKNMKENQYIAALKLRANIPDTRETLTRGR-NR-QFSNCRY
AgTri        GRGISSFKNDSISNDWLQYYRRIPHRKLITALQLRANVYPTREFLSRGREEN-STKACRH
GaGa         GEGVELFRNDPVSNGWINGRGRLAERLRIVALKLRSNVYPTREFLGRGM-AG-TNVGCRH
SPun         GSGIQHFQDDIISNNWLRINKGITEKQFILALKLRANVFPTREFLGRGT-QN-ANRACRH
PlaMe        GSGIEHFDNDTISNDWLQFHRGFSERQFLMGLKIRANVYPTREYQGRGR-TN-KNVNCRN
PBla         GGAVENFINDSISNDWIRYKKDMTEKEFITAIKLRTNTYPTREFLARGK-EN-YERKCRK
             *        :  *  *:     .  .  : .:::* .   *:    **         *

LaMa         ACHARETLNHILQICEVTHDARCARHNRIAKLLEKMLRRRVDRTWIEPIIP-TQKSFIKP
NePa         ACRMPESINHILQVCEKTHDARCARHNRVMKKVNQLLRAKGLKSWMEPIIP-TSTTFMKP
CroHe        ACQNVETLNHILQVCDVTHDARCARHNRVLHRLERLLHKKGLRTTLEPVIP-AGSSFIKP
DeFu         CMDVGETISHISGSCPKTKDIRIRRHNKILELLIERAKRCGWIAYREPHLRNPNGELRKP
AgTri        CNVENETCAHIIGQCPVTKDARIKRHNHICDMLSEEARKKDWVVFKEPYIRDTTKELYKP
GaGa         CTHPRETLGHILGICPSVQEARILRHNKLCKILAAEGKKCEWTVYYEPHLRNAAGELRKP
SPun         CKAKFETCSHILGQCPTLQEARIKRHNKLCTLLKEEAKKLEWEVFDEPHLHNRAGELRKP
PlaMe        CTASYESLSHILGQCPAVQGARIRRHNKLCSMLKREAKELKWVVYEEPHLHTTEKELRKP
PBla         CPAKYESLSHILGQCPGLQNARIWRHNAIGKLLANQAKKKKWTVHQEPHLRNKDNQLRKP
             .    *:  **   *   :  *  *** :   :    :        ** :      : **

LaMa         DLLVDTGKRIIILDVTVVAEPR---MEQSHQLKSSKYGSPDNTAAIVAWTG--TKVPIKH
NePa         DILVESAGKLIVMDVSIVAGHR---LPETWNLKTSKYGSNQATQDLQRWHN--SLISIKH
CroHe        DLIIETDTQTYVLDVSVVAGYR---MTESWDIKVEKYGRPDRVEAIRKWAAIPATKRVEH
DeFu         DLILTKNNTAHVVDVSIRYETSAGSLESAFEQKVSYYS--ELMSAIQQHTK---CSQVAF
AgTri        DLIFVKDGHAFVVDVTVRYETTTTSLEEAAAEKVNKYK--HLETEVRNLTN---AKDVVF
GaGa         DLIFVRDGTALVVDVTVRYEKDSASLSAAAADKAAKYL--GLNAQIQELTG---AEHVTY
SPun         DVILVKDKKALVVDVTVRFEYKEDGLRKAAKEKASYYS--DLTEPIIELTK---AKKVNY
PlaMe        DLIFVKEEMALVVDVTVRFEYKEKVFEDAAAEKVRHYK--DLTSQIKELTG---AKEIEY
PBla         DYIFTKERTAMVVDITVRWEGKMGGLEEAAQEKIKYYE--ELRDTIQKEWG---VKDISF
             * :.       ::*:::        :  :   *   *        :           : .

LaMa         VPVVLSSRGLLYGPSGRGLRTMGLTSRDLCD----LCLLAVAGSLKCYDAYTRGT-----
NePa         VPVIISARGLLFNKSGQGLRDLGLTRRDISD----ICLLSIIGSLKSYDLYKRGTLDDFR
CroHe        LPVIVSNRGLCYQPSGDGLRALGLTSRDISD----LCLLAISGSLRCYDLYMRGTRAV--
DeFu         HGFIVGCRGTWPTCNNNLLDKLGIQYPNPFK--KNICFTTLMDTLKMFQIFMDN------
AgTri        MGFPLGARGKWYTGNFELLNTLGLSKSRQVRVAKTLSTVALLSSVDIVHMFASRARRVQ-
GaGa         FGFPIGARGKWHADNGEVLSELGLSRSRKERVARLLSWRALLGSVDMVNIFASKHRQETL
SPun         FGLPIGARGKWPPENNNLLRELGMEEGRVKRLARLFSLRTLLYSTNMLHMFENIGKGQKA
PlaMe        FGFPLGARGKWPEINEKVLTALGMPDYQQKRTAKRFSKRTLLYSIDVINTFENIGKNNK-
PBla         HGFVCGARGKWPDQNFGILTKLGIESHRLDGLAKLFSRRTVQYSCRMLHTFERDKKNQTG
               .  . **     .   *  :*:           :.  ::  :    . :

LaMa         ------------
NePa         RTKPR---SGIG
CroHe        ------------
DeFu         ------------
AgTri        --------DSG-
GaGa         LDDAPACVEHVA
SPun         Y-----FVNSNL
PlaMe        --------NNVP
PBla         HVDC--------
```

**Figure S12:** Multiple sequence alignment of R2 proteins selected for activity testing: LaMa (*L. marequensis*, Largescale yellowfish), NePa (*N. papilliferus*, killifish), DeFu (*D. fucus*, Northern dusky salamander), CroHe (*C. o. helleri*, Southern Pacific Rattlesnake), PBla (*P. blainvillii,* Blainville's Horned Lizard), SPun (*S. punctatus*, Tuatara), GaGa (*G. gangeticus*, Gharial crocodile), AgTri (*A. tricolor*, Tricoloured blackbird) and PlaMe (*P. megacephalum*, Big-headed turtle). Myb2 domain highlighted in orange. N-terminal ZnFs annotated with red asterisk.
