# Supplementary material for: Lineage-specific evolution, structural diversity, and activity of R2 retrotransposons in animals
Source: Genome Biol. 2026 Apr 14;27:174. doi: 10.1186/s13059-026-04073-3 (PMC13188248; doi:10.1186/s13059-026-04073-3)
Supplement: Supplementary file 13 — Additional file 13. Immunoblot related to Fig. 4b-c. [file 13059_2026_4073_MOESM13_ESM.pdf]

Additional file 13

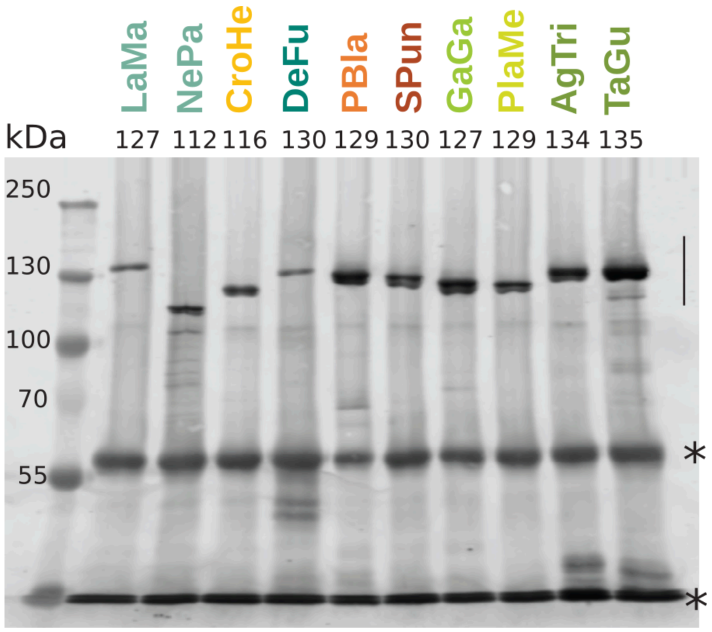

Figure S13: Related to Fig. 4b-c. Immunoblot of affinity purified R2 proteins resolved from FLAG affinity by 8% SDS-PAGE. Asterisks denote background bands from flag antibody purification beads. LaMa (*L. marequensis*, Largescale yellowfish), NePa (*N. papilliferus*, killifish), CroHe (*C. o. helleri*, Southern Pacific Rattlesnake), DeFu (*D. fucus*, Northern dusky salamander), PBla (*P. blainvillii*, Blainville's Horned Lizard), SPun (*S. punctatus*, Tuatara), GaGa (*G. gangeticus*, Gharial crocodile), and PlaMe (*P. megacephalum*, Big-headed turtle), AgTri (*A. tricolor*, Tricolored blackbird), and TaGu (*T. guttata*, Zebra finch)
