# Supplementary material for: Lineage-specific evolution, structural diversity, and activity of R2 retrotransposons in animals
Source: Genome Biol. 2026 Apr 14;27:174. doi: 10.1186/s13059-026-04073-3 (PMC13188248; doi:10.1186/s13059-026-04073-3)
Supplement: Supplementary file 14 — Additional file 14. Scripts used in the study. [file 13059_2026_4073_MOESM14_ESM.pdf]

Additional file 14 - Supplementary Methods

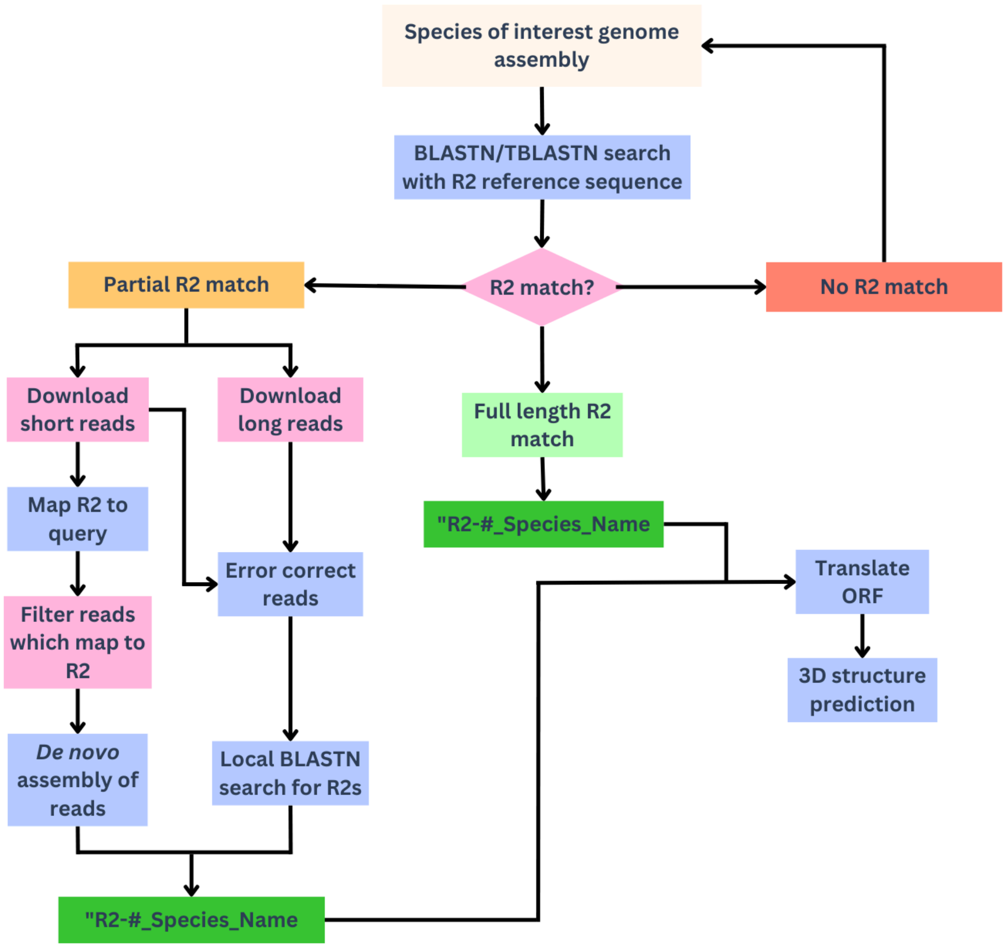

Figure S12: Overview of the R2 discovery pipeline. A sensitive online BLASTN and TBLASTN search was used to find the presence of R2s in the species of interest. Based on the outcome of the search, the R2 was either categorised as full-length or flagged for follow-up using the truncated/misassembled pipeline. Species were removed from further analysis if there is no significant match to R2s. For a truncated or misassembled R2, a subset of long reads and short reads were downloaded from the SRA database. Long reads were made into a BLASTN database to find R2-containing reads. R2-containing reads were error-corrected with short reads using Ratatosk. The error-corrected R2-containing long reads were made into a BLASTN database. Using a subset of R2 query sequences, a sensitive local BLASTN search was performed. Sequences that exceed 4200 nucleotides and contain the required 28s rRNA flanking target sites were selected for ORF translation. Finally, the R2 sequences that fulfil all the requirements (28S rRNA flanks, ORFs, domains) were categorised as full-length R2s.

Downloading long reads from SRA database and sensitive BLASTN search

```
#!/bin/bash
#configure to your HPC requirements
#Required tools
#BLAST+, SRA-Toolkit, seqtk, fastqc

#Download long reads
wget https://sra-LONG-READS
#Convert SRR to fastq
fastq-dump SRR-LONG-READS

#Convert fastq to fasta
seqtk seq -a lONG-READS.fastq > lONG-READS.fasta

#Run fastqc on data
#fastqc lONG-READS.fasta
#Concatanate fasta if there are multiple
#cat *lONG-READS.fasta  > LONG-READS-COMBINED.fasta

#Make blast database using long reads
makeblastdb -in lONG-READS.fasta -dbtype nucl -out LONG-READS_db
#Sensitive blast search using R2 query sequences
blastn -query R2-QuerySeqs.fasta -db LONG-READS_db -word_size 7 -outfmt "6 qseqid
sseqid sseq pident length mismatch gapopen qstart qend sstart send evalue bitscore"
> LONG-READS_best_matches

#Extract best hits as fasta file
awk '{print ">"$2"\n"$3}' LONG-READS_best_matches > seqs.fasta
```

Downloading short reads from SRA database and de novo assembly of R2s

```
#!/bin/bash
#configure to your HPC requirements

#Required tools
#BWA, SAMtools, SPAdes, SRA-Toolkit, fastqc

#Download short reads
wget https://sra-SHORT-READS

#Convert SRR to fastq
fastq-dump --split-e SHORT-READS

#Run fastqc on data
#fastqc SHORT-READS_1.fastq SHORT-READS_2.fastq

#Index R2 query sequence
bwa index R2-QuerySeqs.fasta

#Map short read sequences to query file
bwa mem R2-QuerySeqs.fasta SHORT-READS_1.fastq SHORT-READS_2.fastq > aln.sam

#Convert sam to bam
samtools sort -O BAM -o aln.bam aln.sam

#Index bam file
samtools index aln.bam

#Extract mapped reads
samtools view -F 4 -b -o mapped.bam aln.bam

#Convert mapped reads to fastq file
samtools fastq mapped.bam > mapped.fastq

#Make spades directories
mkdir spades_denovo
makdir spades_ts

#De novo assembly of R2 mapped reads with Spades denovo and with trusted contigs
options
spades.py --12 mapped.fastq -o spades_denovo
spades.py --12 mapped.fastq --trusted-contigs R2-QuerySeqs.fasta -o spades_ts
```

Downloading long and short reads from SRA database and error correction prior sensitive BLASTN search

```
#!/bin/bash
#configure to your HPC requirements

#Required tools
#BLAST+, BWA, SAMtools, SPAdes, SRA-Toolkit, fastqc

#Download short reads
wget https://sra-SHORT-READS

#Download long reads
wget https://sra-long-READS

#Convert SRR to fastq
fastq-dump --split-e SHORT-READS
fastq-dump SRR-LONG-READS

#Run fastqc on data
fastqc SHORT-READS_1.fastq SHORT-READS_2.fastq
fastqc LONG-READS.fasta

#Concatanate long reads
cat *.fastq > LONG-READS.fastq

#Create a list file containing short read headers named short_reads.lst

#Error correct long reads with short reads with Ratatosk
Ratatosk correct -v -c 32 -s short_reads.lst -l LONG-READS.fastq -o
corrected_LONG-READS

#Convert fastq to fasta
seqtk seq -a lONG-READS.fastq > lONG-READS.fasta

#Make blast database using error-corrected long reads
makeblastdb -in lONG-READS.fasta -dbtype nucl -out LONG-READS_db
#Sensitive blast search using R2 query sequences
blastn -query R2-QuerySeqs.fasta -db LONG-READS_db -word_size 7 -outfmt "6 qseqid
sseqid sseq pident length mismatch gapopen qstart qend sstart send evalue bitscore"
> LONG-READS_best_matches

#Extract best hits as fasta file
awk '{print ">"$2"\n"$3}' LONG-READS_best_matches > seqs.fasta
```

Plotting the evolutionary rate of individual residues

```
# Load libraries
library(ggplot2)
#Create MSA of aligned RT sequences
#Use IQTree to run site-specific algorithm
http://www.iqtree.org/doc/Advanced-Tutorial
#Extract the output file with site rates mapped to individual positions in the MSA

# Define sequence
sequence <- unlist(strsplit("YOUR-RT-SEQ-OF-INTEREST-FROM-MSA", NULL))

# Read site rates from the CSV file
numbers <- read.csv("~/site-rates.csv")

# Set this to TRUE if you want to filter out dashes in MSA, or FALSE if you want to
keep
filter_dashes <- TRUE

# Create a data frame mapping positions, residues, and numbers
mapping <- data.frame(
  Position = seq_along(sequence),
  Residue = sequence,
  Number = numbers$Number
)

# Apply the dash filter conditionally
if (filter_dashes) {
  mapping <- mapping %>%
    filter(Residue != "-") %>%
    mutate(Position = seq_along(Residue))
}

# Split residue plot into three rows
mapping <- mapping %>%
  mutate(Group = ceiling(seq_along(Residue) / (n() / 3)))

# Custom colours
subdued_palette <- c("#2F7AC6", "#4D9DC6", "#7EC0B8", "#A7D2A0", "#D1E0A8",
"#F2F4B0")

# Plot using ggplot2
ggplot(mapping, aes(x = Position, y = 1, fill = Number)) +
  geom_tile() +
  geom_text(aes(label = Residue), family = "Courier", size = 3, color = "black") +
  scale_fill_gradientn(colors = subdued_palette) +  # Use custom subdued gradient
  scale_x_continuous(breaks = mapping$Position, labels = mapping$Residue) +
  theme_minimal() +
  theme(
    axis.title.y = element_blank(),
```

```r
    axis.text.y = element_blank(),
    axis.ticks.y = element_blank(),
    panel.grid = element_blank(),
    axis.text.x = element_text(family = "Courier", size = 5, vjust = 1),
    axis.ticks.x = element_line(),
    axis.title.x = element_text(vjust = -0.5)
  ) +
  facet_wrap(~ Group, nrow = 3, scales = "free_x") +
  labs(title = "Protein Sequence Mapping", x = "Residue", fill = "Number")

ggsave(file = "~/sites.svg")

print(mapping)
```

Plotting PSSM charts

```
# Load libraries
if (!requireNamespace("ggrepel", quietly = TRUE) | !requireNamespace("ggforce",
quietly = TRUE)) {
  install.packages(c("ggrepel", "ggforce"))
}
library(dplyr)
library(ggplot2)
library(ggrepel)
library(ggforce)

#Run PSSM using PSIblast for RTs of interest using 1) BoMo then 2) TaGu RTs as the
query
#Create csv: R2 IDs, corresponding bitscores, common species group, number of
N-terminal ZnFs
#For example:
#ID_Code, BoMo, TaGu, ZnF_No, Common.Group
#R2-1_OL, 150, 400, 3, Fish (Bony)

# Load data
df <- read.csv("~/PSSMscores.csv")
str(df)

# Validate columns
required_cols <- c("BoMo", "TaGu", "Common.Group", "ID_Code", "ZnF_No")
if (!all(required_cols %in% colnames(df))) stop("Required columns are missing.")

# Prepare the data
df_filtered <- df %>%
  select( BoMo = BoMo,  TaGu = TaGu, ZnF_No, Common.Group, ID_Code) %>%
#Enter common group you want to plot, e.g. "Fish (Bony)"
  filter(Common.Group %in% c("Common.Group of interest"))
if (nrow(df_filtered) == 0) stop("The filtered data frame is empty.")

#Filter any R2s from the plot if needed
#Filter the data frame to remove rows where 'label' is in 'labels_to_remove'
df_filtered <- df_filtered %>%
  filter(!Code %in% c("R2-1_Example"))

# Variables for axes
x_var <- "TaGu"
y_var <- "BoMo"
# Create the PSSM plot
ggplot(df_filtered, aes_string(x = x_var, y = y_var, color = "Common.Group")) +
  geom_point(show.legend = FALSE) +  # Remove legend for points
  #geom_point(position = position_jitter(width = 0.2, height = 0.2), show.legend =
FALSE) +  # Remove legend for jittered points
  # Draw hulls based on ZnF_No, ignoring Common.Group: this groups and colour codes
R2s based on the number of N-terminal zinc fingers and not common species group
```

```
  geom_mark_hull(aes(group = ZnF_No, fill = ZnF_No),
                 concavity = 10, expand = unit(2.5, "mm"),
                 alpha = 0.15, colour = "black", size = 0.0, show.legend = FALSE) +
# Remove legend for hulls
# Colour code for each species group
  labs(x = x_var, y = y_var) +  # No legend for color and fill
  geom_text_repel(aes(label = Code), size = 3) +
  theme_minimal() +
  theme(
    panel.grid.major = element_line(),
    panel.grid.minor = element_line(),
    axis.line = element_line(color = "black", size = 0.3)  # Keep axis lines
  ) +
  scale_color_manual(values = c(
    "Fish (Bony)" = "#76B49E", "Tunicata" = "#6495EC", "Arthropoda" = "#B196FF",
    "R/Bird" = "#6B8E22", "Echinodermata" = "#4169E1", "R/Snake" = "#FFBF00",
    "R/Lizard" = "#E97929", "R/Crocodile/Alligator" = "#9ACD31", "Cnidaria" =
"#5661D6",
    "Ctenophora" = "#271F5B", "Fish (Cartilaginous)" = "#87CEEB", "R/Turtle" =
"#E0E41D",
    "Amphibian" = "#008080", "Mollusca" = "#B65090", "Annelida" = "#E08BE0",
    "Platyhelminthes" = "#DFB0FF", "R/Lizard (Like)" = "#AE3E0D", "Porifera" = "red"
  )) +
  scale_fill_manual(values = c("A" = "#0B8731", "D" = "#2B10FF", "B" = "#FC8D62",
"C" = "#FC8D62"))

ggsave(filename = "fishesPSSM.svg", width = 10, height = 10, path = "~/your-path")
```
