# Supplementary material for: Lineage-specific evolution, structural diversity, and activity of R2 retrotransposons in animals
Source: Genome Biol. 2026 Apr 14;27:174. doi: 10.1186/s13059-026-04073-3 (PMC13188248; doi:10.1186/s13059-026-04073-3)
Supplement: Supplementary file 15 — Additional file 15. RAxML tree of R2s. [file 13059_2026_4073_MOESM15_ESM.pdf]

Additional file 15

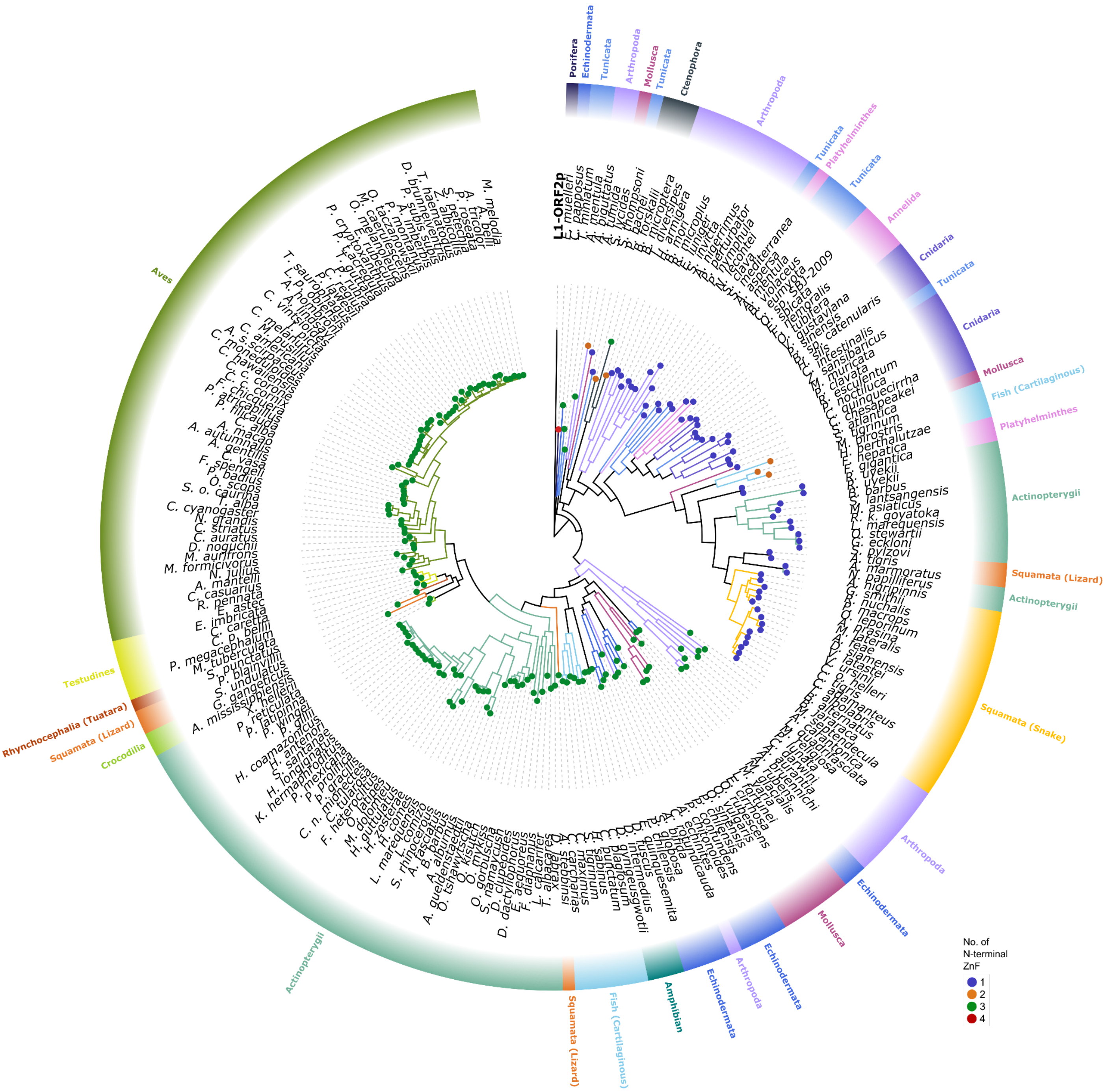

Figure S15: Phylogenetic tree of R2s constructed with RaxML rooted with human L1 ORF2 (R2 trimmed amino acid sequence, model JTT, 1000 replicates). Bootstrap values under 80 are shown at nodes.
